# Heterologous expression and transcript analysis of gibberellin biosynthetic genes of grasses reveals novel functionality in the *GA3ox* family

**DOI:** 10.1186/s12870-015-0520-7

**Published:** 2015-06-05

**Authors:** Stephen Pearce, Alison K Huttly, Ian M Prosser, Yi-dan Li, Simon P Vaughan, Barbora Gallova, Archana Patil, Jane A Coghill, Jorge Dubcovsky, Peter Hedden, Andrew L Phillips

**Affiliations:** Department of Plant Sciences, University of California, Davis, CA 95616 USA; Department of Plant Biology and Crop Science, Rothamsted Research, Harpenden, AL5 2JQ UK; Biotechnology Research Centre, Jilin Academy of Agricultural Sciences, Changchun, 130033 China; University of Bristol Transcriptomics Facility, School of Biological Sciences, Bristol, BS8 1UG UK; Howard Hughes Medical Institute, Chevy Chase, MD 20815 USA

**Keywords:** Gibberellin, Wheat, Biosynthesis, Signalling, Gene sequences, De novo assembly, Transcriptomics, Heterologous expression, GA 1-oxidase

## Abstract

**Background:**

The gibberellin (GA) pathway plays a central role in the regulation of plant development, with the 2-oxoglutarate-dependent dioxygenases (2-ODDs: GA20ox, GA3ox, GA2ox) that catalyse the later steps in the biosynthetic pathway of particularly importance in regulating bioactive GA levels. Although GA has important impacts on crop yield and quality, our understanding of the regulation of GA biosynthesis during wheat and barley development remains limited. In this study we identified or assembled genes encoding the GA 2-ODDs of wheat, barley and *Brachypodium distachyon* and characterised the wheat genes by heterologous expression and transcript analysis.

**Results:**

The wheat, barley and Brachypodium genomes each contain orthologous copies of the *GA20ox*, *GA3ox* and *GA2ox* genes identified in rice, with the exception of *OsGA3ox1* and *OsGA2ox5* which are absent in these species. Some additional paralogs of 2-ODD genes were identified: notably, a novel gene in the wheat B genome related to *GA3ox2* was shown to encode a GA 1-oxidase, named as TaGA1ox-B1. This enzyme is likely to be responsible for the abundant 1β-hydroxylated GAs present in developing wheat grains. We also identified a related gene in barley, located in a syntenic position to *TaGA1ox-B1*, that encodes a GA 3,18-dihydroxylase which similarly accounts for the accumulation of unusual GAs in barley grains. Transcript analysis showed that some paralogs of the different classes of 2-ODD were expressed mainly in a single tissue or at specific developmental stages. In particular, *TaGA20ox3*, *TaGA1ox1*, *TaGA3ox3* and *TaGA2ox7* were predominantly expressed in developing grain. More detailed analysis of grain-specific gene expression showed that while the transcripts of biosynthetic genes were most abundant in the endosperm, genes encoding inactivation and signalling components were more highly expressed in the seed coat and pericarp.

**Conclusions:**

The comprehensive expression and functional characterisation of the multigene families encoding the 2-ODD enzymes of the GA pathway in wheat and barley will provide the basis for a better understanding of GA-regulated development in these species. This analysis revealed the existence of a novel, endosperm-specific GA 1-oxidase in wheat and a related GA 3,18-dihydroxylase enzyme in barley that may play important roles during grain expansion and development.

**Electronic supplementary material:**

The online version of this article (doi:10.1186/s12870-015-0520-7) contains supplementary material, which is available to authorized users.

## Background

Gibberellins (GAs) are a group of plant secondary products based on the diterpenoid *ent*-gibberellane skeleton; a small subset of bioactive GAs such as GA_4_ and GA_1_ act as plant hormones and participate in a wide range of developmental processes. Although classically involved in the promotion of growth processes such as germination and stem elongation, GA signalling has also been shown to be important in root elongation [1], lateral root formation [2], skotomorphogenesis [3], cambial activity [4], leaf expansion [5], trichome development [6], floral induction [7], anther and pollen development (reviewed in Plackett et al.[8]), fruit growth [9] and seed development [10]. Furthermore, GAs mediate environmental effects on growth and development through modulation of both biosynthetic and signalling components [11]. The central components of GA signalling, GRAS-domain proteins containing an N-terminal “DELLA” motif that repress growth, also act as nodes in the interactions with several other plant hormones, including jasmonate [12], brassinosteroids [13] and strigolactones [14]. In short, GAs play a central role in plant development and environmental responses, with impacts on crop yield and quality.

The importance of GA signalling in determining plant stature is clear from evidence in both wild and crop species showing phenotypic effects of genetic variation in GA biosynthetic and signalling genes. In wheat (*Triticum aestivum* L*.*), semi-dwarfing alleles of the *Rht* DELLA genes were key to increasing yield during the Green Revolution as not only did the shorter stature protect against lodging under high fertiliser application rates, but also enhanced harvest index by reducing straw biomass and, in many genetic backgrounds, increasing grain numbers per ear [15]. In rice (*Oryza sativa* L.), a similar height phenotype was conferred by loss-of-function mutations in a key GA biosynthetic gene, *OsGA20ox2* [16], and there is evidence that semi-dwarfing of barley (*Hordeum vulgare* L.) by the *sdw1/denso* gene is associated with reduced expression of the orthologous *HvGA20ox2* gene [17].

Although *Rht* semi-dwarfing alleles are widespread in modern wheat varieties, the involvement of Rht in all GA responses results in pleiotropic effects on many other traits. For example, even mild alleles such as *Rht-B1b* and *Rht-D1b* impart reduced leaf area [18]. These alleles also have a strong effect on coleoptile elongation which prevents deeper sowing under dry conditions [19]. Better targeting of the dwarfing effect to stem tissues might therefore confer significant advantages. In contrast to Rht, the enzymes acting during the latter stages of GA biosynthesis are encoded by multiple paralogs with overlapping domains of expression and, in Arabidopsis, mutations in individual genes have more localised effects [20-22]. This suggests that the characterisation of the GA biosynthetic genes of wheat has the potential to identify targets for the development of novel semi-dwarfing alleles with fewer undesirable pleiotropic effects than the current *Rht* alleles.

GAs are also thought to play a critical role in wheat grain development: endogenous GA levels are very high in developing grain and increase during grain expansion [10] and wheat lines containing *Rht* alleles have smaller grains [15]. Despite grain size being an important component of wheat yield and quality, little is known regarding the spatial or temporal regulation of GA biosynthesis and signalling in the grain. A fuller understanding of the role of GA during grain development is required to engineer improvements in this trait in modern wheat varieties.

**Figure 1:**
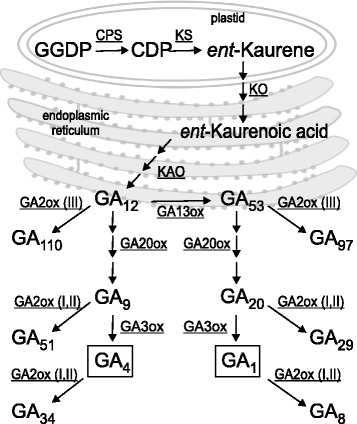
Principal reactions of the GA biosynthetic pathway in plants. Enzymes are underlined; numerals after *GA2ox* genes indicate the class of enzyme as defined by Lee & Zeevaart (2005). The bioactive GAs are boxed

The GA biosynthetic pathway has been extensively characterized in both rice and Arabidopsis (reviewed by Yamaguchi, 2008 [23]), and the early genes in the pathway, from copalyl diphosphate synthase (CPS) to *ent*-kaurenoic acid oxidase (KAO) that produce the GA precursors GA_12_ and GA_53_ have also been identified and characterised in wheat [24-26]. The final steps in GA biosynthesis and inactivation are catalysed by soluble 2-oxoglutarate-dependent dioxygenases (2-ODDs) (Fig. 1). GA 20-oxidase catalyses the multi-step oxidation of GA_12_ and GA_53_ to form the C_19_ skeleton, while GA 3-oxidase produces the final bioactive products, GA_4_ and GA_1_. A third class of 2-ODD, GA 2-oxidase, is involved in inactivation, with two sub-classes of enzyme that act against either bioactive GAs (GA_4_, GA_1_) and their immediate C_19_ precursors (GA_9_, GA_20_) [27] or against C_20_-GAs earlier in the pathway (e.g., GA_12_, GA_53_) [28]. However, our knowledge of the size, structure and function of the gene families encoding these enzymes in wheat is limited.

Most of the evidence points to dynamic regulation of GA biosynthesis through regulation of the 2-ODD genes which, in contrast to the terpene cyclase and cytochrome P450 genes earlier in the pathway [24], are encoded by small multigene families [23]. Although a small number of the 2-ODDs involved in the GA pathway of wheat have been identified previously [24, 29], this study represents the first comprehensive analysis of the paralogous and homoeologous genes encoding the enzymes in this crop species. In this report we identify and characterise the biochemical function of GA-biosynthetic 2-ODD genes in wheat and demonstrate novel biochemical functions. We also catalogue the orthologous genes, where data is available, in durum wheat, barley and *Brachypodium distachyon*. Finally, we identify tissue-specific patterns of expression in wheat that suggest specialized roles in plant development for the different paralogs.

## Results

### Identification of wheat genes involved in GA metabolism

Our strategy to identify the wheat complement of genes of the 2-ODD class from the GA biosynthetic pathway, previously catalogued in rice [30, 31] is outlined in Additional file 1 (Figure S1) and involved first identifying orthologous genes from Brachypodium by BLASTP search at www.phytozome.org (Table 1 and Additional file 2) using the rice peptide sequences as queries. Coding sequences from the Brachypodium genes were then used to BLAST partially-assembled genomic survey sequence from the International Wheat Genome Sequencing Consortium (IWGSC), generated by Illumina sequencing of DNA from individual wheat chromosome arms [32]. We were thus able to identify high-quality contigs containing wheat orthologs of many of the rice and Brachypodium GA 2-ODD genes.

**Table 1:** Rice, Brachypodium and bread wheat genes encoding 2-ODDs from the GA pathway

Target	Rice gene	Brachypodium gene	*T. aestivum* chromosome arm assembly		
			A	B	D
**GA20ox1**	*Os03g63970*	*Bradi1g00950*	FL (4AL)	FL (5BL)	FL (5DL)
**GA20ox2**	*Os01g66100*	*Bradi2g57030*	Partial^a^ (3A)	FL^a^(3B)	FL^a^ (3D)
**GA20ox3**	*Os07g07420*	*Bradi1g56200*	Partial^a^ (3A)	FL (3B)	FL^a^ (3D)
**GA20ox4**	*Os05g34854*	*Bradi2g24980*	FL^a^ (1AL)	FL^a,b^ (1BL)	FL^a,b^ (1DL)
**GA3ox1**	*Os05g08540*	*-*	-	-	-
**GA3ox2**	*Os01g08220*	*Bradi2g04840(a) Bradi4g23570(b)*	Partial^a,c^ (3A)	FL (3B)	FL^a^ (3D)
**GA3ox3**	*-*	*-*	FL^a^ (2AL)	FL^a^ (2BL)	FL^a,d^ (2DL)
**GA1ox1**	*-*	*-*	-	FL (2BL)	-
**GA2ox1**	*Os05g06670*	*Bradi2g34840*	FL^a^ (1AS)	FL (1BS)	FL (1DS)
**GA2ox2**	*Os01g22910*	*Bradi2g12440*	-	Partial (7BL)	FL^a^ (7D)
**GA2ox3**	*Os01g55240*	*Bradi2g50280*	Partial^e^ (3AL)	FL (3B)	FL^a^ (3D)
**GA2ox4**	*Os05g43880*	*Bradi2g19900*	FL^a^ (1AL)	FL^a^ (1B)	FL^a^ (5BL)
**GA2ox5**	*Os07g01340*	*Bradi1g59570*	-	-	-
**GA2ox6**	*Os04g44150*	*Bradi5g16040*	FL^a^ (2AL)	FL (2BL)	FL^a^ (2DL)
**GA2ox7**	*Os01g11150*	*Bradi2g06670*	Partial (3AS)	FL^a^ (3B)	FL^a^ (3DS)
**GA2ox8**	*Os05g48700*	*Bradi2g16730(a) Bradi2g16750(b)*	FL^a^ (1AL)	Partial (1BL)	FL^a,b^ (5BL)
**GA2ox9**	*Os02g41954*	*Bradi3g49390*	FL (6AL)	Partial (6BL)	FL^a,b^ (6DL)
**GA2ox10**	*Os05g11810*	*Bradi2g32580*	FL^a^ (1AS)	FL (1BS)	FL (1DS)
**GA2ox11**	*-*	*-*	FL (4AS)	FL (4BL)	FL^a^ (4DL)
**GA2ox12**	*-*	*-*	-	FL (4BL)	-
**GA2ox13**	*-*	*-*	-	FL (4BL)	-

Notes: FL - full length; ^a^Generated by reassembly of IWGSC chromosome arm reads; ^b^Missing data in intron; ^c^FL cDNA from cv. Maris Huntsman [29]; ^d^7 bp insertion in exon 2; ^e^FL cDNA from cv. Avalon (Prosser & Phillips, unpublished). Accession numbers for the wheat genes are in Additional file 2.

Genes absent from, or incomplete in, the wheat genomic survey data were initially assembled from shotgun genomic reads of wheat cv. Chinese Spring [33] located at http://www.cerealsdb.uk.net. The raw 454 reads were identified by BLASTN with the Brachypodium CDS sequences and assembled at high stringency; for most targets this resulted in the identification of genomic contigs covering the bulk of the coding region of the gene; however, the low genome coverage of the raw data, approximately 5x, [33] coupled with the relatively high error rate of 454 sequencing and the hexaploid genome of wheat resulted in contigs that contained ambiguous bases and, in many cases, chimeric sequences derived from more than one homoeolog. Hence, these consensus sequences were used in BLAST searches of the unassembled Illumina data from individual chromosome arms [32], followed by mapping the Illumina reads to the 454 assemblies to generate homoeolog-specific sequences, as annotated in Table 1 and, in greater detail, in Additional file 2. Thus, across the *GA20ox*, *GA3ox* and *GA2ox* gene families, we were able to identify or assemble at least one complete homoeolog, and often all three, for most of the target 2-ODD genes, as described in detail below. In addition, we identified or assembled wheat sequences encoding the GA 13-hydroxylase (GA13ox) biosynthetic enzymes and for the GID1 and GID2 signalling components.

The genomic survey data from bread wheat cv. Chinese Spring was complemented with assembled RNA-seq data from tetraploid durum wheat (*T. turgidum* L. subsp. *durum* Desf.) cv. Kronos and the diploid progenitor *T. urartu* [34]. In addition, we identified, where possible, likely orthologs of each gene in the genomic survey sequences of *T. urartu* [35] and *Aegilops tauschii* [36], the diploid progenitors of the A and D genomes, respectively, of bread wheat (Additional file 2). Homoeolog-specific sequences from the tetraploid species were assigned to the A or B genomes by BLAST to the bread wheat chromosome arm-specific genomic survey data above (Table 1). Finally, we identified likely orthologs of each of the wheat genes in barley (Additional file 2), within the recent draft genome sequence of this species [37].

### Structure and biochemical function of wheat GA 2-ODD genes

**Figure 2:**
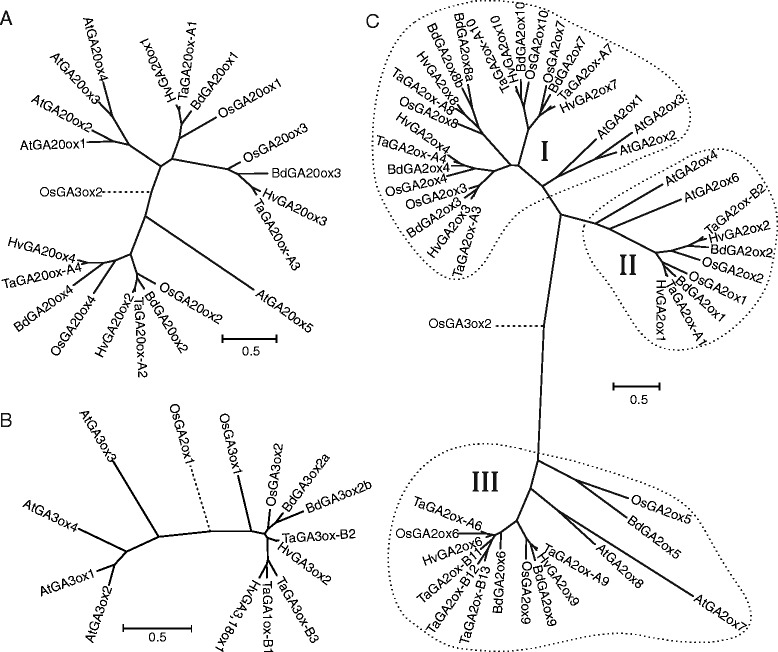
Phylogenetic relationships between gibberellin 2-ODD amino acid sequences from bread wheat (Ta), barley (Hv), rice (Os), Brachypodium (Bd) and *Arabidopsis thaliana* (At). Dotted lines indicate sequences used as outgroups for the rooted tree. Scale bars indicate number of amino acid substitutions per site. A: GA20ox; B: GA3ox (including TaGA1ox1 and HvGA3,18ox1); C: GA2ox. Clades labelled I, II and III in panel C reflect classes of GA2ox as defined by Lee and Zeevaart (2005)

We identified likely Brachypodium orthologs for each of the four *GA20ox* genes, two *GA3ox* genes and ten *GA2ox* genes previously described in rice [30, 31]. The only exception was *GA3ox1*, which appears to be absent from Brachypodium, as shown in Table 1 and in the phylogenetic analysis presented in Fig. 2B; however, this species contains two genes related to *GA3ox2*, as discussed below. Brachypodium also contains a tandem duplication of *GA2ox8*. As neither hexaploid bread wheat, nor its tetraploid or diploid progenitors, possesses a fully-sequenced genome, we cannot be unequivocal about the number of GA 2-ODD genes present in wheat. However, with the exception of *GA2ox5*, we identified in bread wheat at least one homolog of each Brachypodium gene, and usually complete or partial sequence evidence of homoeologs on each of the three chromosomes. Thus we identified in bread wheat four homoeologous sets of *GA20ox* genes, at least two sets of *GA3ox* genes and at least nine sets of *GA2ox* genes, as detailed below.

### GA 20-oxidases

Phylogenetic analysis of the 2-ODD genes showed that the grass *GA20ox* genes fall into four paralogous clades (Fig. 2A) each including one of the four rice *GA20ox* genes, *OsGA20ox1* through *OsGA20ox4*. The assignment of the wheat *GA20ox1*, *GA20ox2* and *GA20ox4* genes to the corresponding rice groups is supported by syntenic relationships between rice and wheat chromosomes [38]. In contrast, *GA20ox3* which was expected to be found on wheat chromosome group 2 based on its position in rice and Brachypodium, was found on the three homoeologs of chromosome group 3 [39]. We also identified full-length or partial sequences for all *GA20ox* paralogs in the tetraploid and diploid wheat species and full-length coding sequences from barley (Additional file 2). As previously reported [20], phylogenetic analysis indicates that the four paralogs of *GA20ox* in grass species are not directly orthologous to any of the five paralogs identified in Arabidopsis: four of the five Arabidopsis genes lie in a single clade of the tree (Fig. 2A), suggesting that the expansion in paralogs of *GA20ox* occurred after the separation of the monocot and eudicot lineages.

We have previously reported the biochemical function of all three homoeologs of *TaGA20ox1* by heterologous expression in the pET3d vector [29]. In this study, we present a similar characterization for *TaGA20ox2*, *TaGA20ox3* and *TaGA20ox4*. We expressed the coding regions of one representative homoeolog of each of these three wheat paralogs as fusion proteins in *E. coli* (see Methods) and demonstrated their ability to carry out the series of sequential oxidations of GA_12_ to GA_9_ (Additional file 1: Figure S2). These results demonstrate that all four wheat paralogs encode fully active GA 20-oxidases (Fig. 1).

### GA 3-oxidases

**Figure 3:**
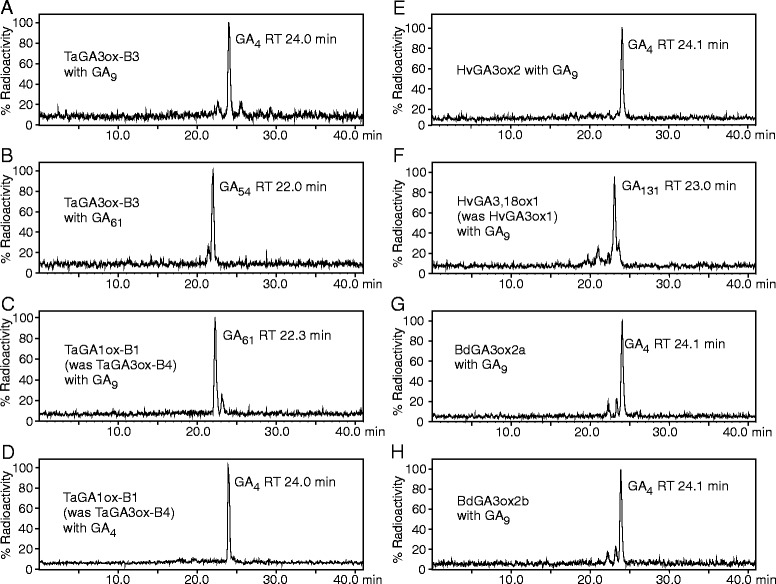
HPLC separation of incubations of GA3ox-like enzymes from wheat, barley and Brachypodium with [1-^14^C]-GA substrates. A,B: TaGA3ox-B3; C,D: TaGA1ox-B1 (was TaGA3ox-B4); E: HvGA3ox2; F: HvGA3,18ox1 (was HvGA3ox1); G: BdGA3ox2a; H: BdGA3ox2b. X-axis shows HPLC retention time in while the Y-axis is scaled such that the height of the largest peak of radioactivity is 100 %.

Phylogenetic relationships between rice, Brachypodium, barley and wheat are less clear in the *GA3ox* gene family. Neither Brachypodium, barley nor either of the wheat polyploid species or their progenitors appear to possess a true ortholog of *OsGA3ox1* (Fig. 2B). However, Brachypodium and wheat possess likely orthologs of *OsGA3ox2*, with three homoeologs in bread wheat as described previously [29]. Similar sequences were also found for the A genome in *T. durum* (Table 1), in *T. urartu, Ae. tauschii* (as a partial genomic sequence), and barley (Additional file 2). Brachypodium contains a second sequence, *Bradi4g23570*, related to *OsGA3ox2*, but the predicted coding regions of the genes from several accessions of this species at www.brachypodium.org contain a frame shift in exon 2 due to the apparent insertion of a G residue at nucleotide 765. However, PCR amplification and sequencing of this region from *B. distachyon* Bd21 genomic DNA clearly showed the inserted base present in the database sequence to be an artefact. Removal of G765 from the database sequence resulted in a complete open reading frame most closely related to *BdGA3ox2a* (*Bradi2g04840*) (Fig. 2B) and *Bradi4g23570* was therefore assigned as *BdGA3ox2b*. Heterologous expression of synthetic coding sequences of *BdGA3ox2a* and *BdGA3ox2b* in *E. coli* followed by incubation of bacterial lysates of these cultures with radiolabelled substrates showed that both Brachypodium genes encode GA 3-oxidase enzymes, converting GA_9_ to GA_4_ (Fig. 3G,H).

Syntenic relationships and phylogenetic analysis (Fig. 2B) support the assignment of wheat *GA3ox2* genes on the group 3 chromosomes as orthologous to rice *GA3ox2*. However, in bread wheat we identified four further related sequences encoding potential GA 3-oxidases, all located on the long arms of the group 2 chromosomes, some of which were also found in the tetraploid and diploid species (Additional file 2). Phylogenetic analysis and the location on chromosomes 2AL, 2BL and 2DL suggested that the three most closely-related of these four novel sequences probably form a homoeologous group (Additional file 1: Figure S12) and therefore were named as *TaGA3ox-A3*, *TaGA3ox-B3* and *TaGA3ox-D3* (Table 1). Near-identical sequences to the A and B homoeologs were identified in the durum wheat transcript assembly [34] and a partial sequence from *T. urartu* was also identified; however, *TaGA3ox3* was not found in the *Ae. tauschii* assembly. The fourth novel gene, located on bread wheat chromosome 2BL and also identified in the durum wheat transcript assembly, was provisionally named *TaGA3ox-B4*; no homoeologs of this sequence were identified in the A or D genomes of wheat or in the A and D diploid progenitor species, although a similar sequence (79.3 % amino acid identity) had been previously identified in barley and annotated as *HvGA3ox1* [39].

We have previously determined the biochemical function of the bread wheat GA3ox2 genes by heterologous expression of the cDNAs in *E. coli* [29]: products from all three homoeologs converted GA_9_ to GA_4_ and GA_20_ to GA_1_, demonstrating GA 3β-hydroxylase (GA 3-oxidase) activity. In this study we present the functional characterization of *TaGA3ox3* and *TaGA3ox4* through expression in *E. coli* of synthetic cDNAs. When lysates from induced bacterial cells containing synthetic cDNA constructs were incubated with [1-^14^C]GA_9_, *TaGA3ox-B3* was shown to encode a functional GA 3-oxidase, converting the substrate to [1-^14^C]GA_4_ (Fig. 3A), while expression products of *TaGA3ox-A3* did not have any detectable catalytic activity; *TaGA3ox-D3* was not tested as the Chinese Spring sequence contains a 7 bp insertion in exon 2, indicating that this gene is unlikely to be functionally active.

**Figure 4:**
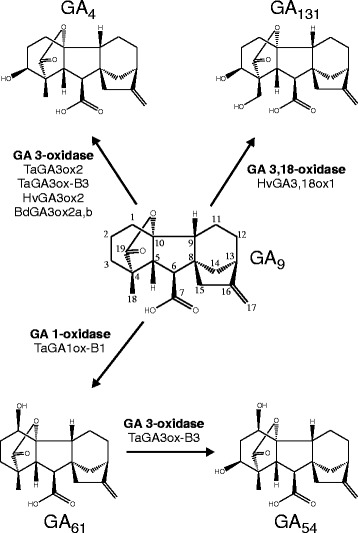
Reactions catalysed *in vitro* by homologs of GA3ox from bread wheat (Ta), barley (Hv) and Brachypodium (Bd)

An unexpected result was observed for TaGA3ox-B4, which converted [1-^14^C]GA_9_ to a product with an HPLC retention time different to that of [1-^14^C]GA_4_ (Fig. 3C). This product was analysed by combined gas chromatography-mass spectroscopy (GC-MS) and had a mass spectrum consistent with [1-^14^C]GA_61_ (1β-hydroxy-GA_9_) [40], identifying TaGA3ox-B4 as a GA 1β-hydroxylase (GA 1-oxidase; Fig. 4), the first time an enzyme with such a catalytic activity has been described. Based on this result we propose to rename this wheat gene as *TaGA1ox-B1.*

GA_61_ was originally identified in the endosperm of developing grains of bread wheat [41] along with the more abundant 1β,3β-dihydroxylated form, GA_54_ (1-hydroxy-GA_4_; Fig. 4) [42]. To determine the likely sequence of reactions leading to the production of GA_54_ in grain, expression products of *TaGA3ox-B3* and *TaGA1ox-B1* were separately incubated with ^14^C-labelled GA_9_, GA_4_ and GA_61_ in the presence of co-substrates and cofactors and the products analysed by HPLC. The 3-oxidase enzyme, TaGA3ox-B3, was active against both GA_9_ (producing GA_4_) and GA_61_ (producing GA_54_, Fig. 3A,B) whereas the GA 1-oxidase, TaGA1ox-B1 acted only upon GA_9_ (producing GA_61_), and not upon GA_4_ (Fig. 3C,D). This suggests that the order of reactions in developing wheat grains is GA_9_ → GA_61_ → GA_54_, catalysed by TaGA1ox-B1 and TaGA3ox-B3, respectively (Fig. 4).

As reported above, we identified a published sequence in barley, annotated as *HvGA3ox1* [39] but most closely related to *TaGA1ox1* and *TaGA3ox3* (Fig. 2B) and located on the syntenic barley chromosome arm, 2HL. We investigated the catalytic activity of *HvGA3ox1* and *HvGA3ox2* by heterologous expression of synthetic cDNAs in *E. coli* as above. When incubated with [1-^14^C]GA_9_, HvGA3ox2 yielded GA_4_ (Fig. 3E), as expected for a GA 3-oxidase, whereas HvGA3ox1 generated a product with an HPLC retention time different from both GA_4_ and GA_61_. GC-MS analysis of this novel product revealed that it was GA_131_ (3β,18-dihydroxy-GA_9_; Fig. 3F) [43], thus identifying HvGA3ox1 as a bifunctional enzyme, a GA 3β,18-dihydroxylase (GA 3,18-oxidase). We therefore propose to rename *HvGA3ox1* as *HvGA3,18ox1*. It has been previously shown that whereas developing grains of wheat accumulate 1-hydroxy-GAs, grains from barley accumulate 18-hydroxy-GAs including GA_131_ (18-hydroxy-GA_4_) and GA_132_ (18-hydroxy-GA_1_) [43-45]. It therefore seems highly likely that *HvGA3,18ox1* is the only enzyme required for the production of GA_131_ and GA_132_ from GA_9_ and GA_20_, respectively, in developing barley grains, while biosynthesis of bioactive GA_4_ and GA_1_ from these substrates in the rest of the plant is catalysed by *HvGA3ox2* (Fig. 4).

As phylogenetic analysis (Fig. 2B) suggested a close relationship between *TaGA3ox3*, *TaGA1ox-B1* and *HvGA3,18ox1*, we investigated their chromosomal locations. All these novel genes are located on the long arms of the group 2 chromosomes of wheat and barley, respectively. To further refine the syntenic relationships, the POPSEQ mapping data of wheat [32] was interrogated and showed the contig containing *TaGA3ox-A3* to be located on chromosome 2AL at 120.3 cM while *TaGA1ox-B1* was on chromosome 2BL at 134.03 cM; the contigs containing *TaGA3ox-B3* and *TaGA3ox-D3* were absent from the POPSEQ data. Predicted genes from the wheat contigs mapped to the same location as *TaGA3ox-A3* and *TaGA1ox-B1* were screened by BLASTN against the pseudomolecule of barley chromosome 2H [37], on which *HvGA3,18ox1* is located at 608.9 Mbp; 95 % of the wheat genes in the same mapping bin as *TaGA3ox-A3* had a top BLAST hit on barley chromosome 2H within 3 cM of *HvGA3,18ox1*, while 90 % of the genes co-locating with *TaGA1ox-B1* also had a top BLAST hit within the same window. This suggests that *TaGA3ox3, TaGA1ox-B1* and *HvGA3,18ox1* are in orthologous positions in the wheat and barley genomes and are likely to be derived from a common ancestral gene, as suggested by the phylogenetic analysis (Fig. 2B). Similar BLAST searches of the rice and Brachypodium genomes with the wheat genes flanking *TaGA3ox-A3* and *TaGA1ox-B1* did not identify any linkage to *OsGA3ox1*, *OsGA3ox2*, *BdGA3ox2a* or *BdGA3ox2b*.

### GA 2-oxidases

Ten *GA2ox* genes, *OsGA2ox1* through *OsGA2ox10*, have been described in rice (Table 1); although the biochemical function of some of the rice genes has been demonstrated by heterologous expression in *E. coli* (e.g., *OsGA2ox1*, [46]; *OsGA2ox5* [47]), by transactivation in rice by T-DNA insertion (e.g., *OsGA2ox3*, [46]) or by ectopic expression in transgenic plants (e.g., *OsGA2ox5*, [46]), most have not been fully characterised. Based on phylogenetic analysis of protein sequences from a number of dicot species, Lee and Zeevaart [48] proposed three structural classes of GA2ox enzymes. A phylogenetic analysis of GA2ox sequences from Arabidopsis, rice, and Brachypodium suggested that the grass enzymes can each be assigned to one of these classes (Fig. 2C). Class I, exemplified by AtGA2ox1, -2 and -3, includes the rice and Brachypodium paralogs GA2ox3, -4, -7, -8 and -10; Class II contains AtGA2ox4 and -6, and the grass paralogs GA2ox1 and -2; Class III is represented by AtGA2ox7 and -8 and the grass paralogs GA2ox5, -6 and -9. Previous data suggest that most GA2ox enzymes in Classes I and II almost exclusively use C_19_-GAs as substrates, while class III enzymes reportedly metabolize only C_20_-GAs.

In wheat, bioinformatic analysis of assembled and raw chromosome arm data revealed likely orthologs for each of the rice and Brachypodium GA2ox genes (Table 1) with the sole exception of *OsGA2ox5*, which was not detected in any wheat species, or in barley (Additional file 2). In bread wheat, *T. urartu* and *Ae. tauschii* we identified an additional group of *GA2ox* genes on the homoeologous group 4 chromosomes, that were most similar to *TaGA2ox6*; the bread wheat genes were named as *TaGA2ox-A11, -B11* and *-D11* and we identified two further related paralogs in the bread wheat genome assembly, *TaGA2ox-B12* and *TaGA2ox-B13*, both on chromosome 4BL (Table 1) and not detected in the A and D genomes.

In general, the chromosomal locations of the wheat GA2ox genes as indicated by the chromosome arm survey data was as predicted by synteny with rice [38]. However, *GA2ox2* is located on rice chromosome 1 and the orthologous genes in wheat would be expected to be found on the group 3 chromosomes but instead are on the group 7 chromosomes (Table 1). Also, the A and B homoeologs of *TaGA2ox4* and *TaGA2ox8* were found on the long arms of the wheat group 1 chromosomes, as predicted from synteny with rice, but in both cases no homoeolog was found on chromosome 1D, although partial sequences with high nucleotide sequence identity (94–98 %) were identified in the *Ae. tauschii* assembly (Additional file 2). However, genes very closely related to *TaGA2ox4* and *TaGA2ox8* and to the candidate orthologous sequences in *Ae. tauschii* were identified in the chromosome arm assembly for 5BL, and these genes were tentatively named *TaGA2ox-D4(5BL)* and *TaGA2ox-D8(5BL)*.

To confirm the biochemical activities of wheat *GA2ox* genes we expressed one representative of each paralogous group as a fusion protein in *E. coli* and tested for activity against C_20_ (GA_12_) and C_19_ (GA_9_) substrates. The activity detected in each bacterial lysate was as predicted by the phylogenetic analysis (Fig. 2C): TaGA2ox-D1, -D2, -B3, -D4, -D7, -D8 and -D10 were all active against the C_19_ substrate, [1-^14^C]GA_9_, while TaGA2ox-D6 and TaGA2ox-D9 were active against the C_20_ substrate, [1-^14^C]GA_12_, (Additional file 1: Figure S3); no activity against either substrate was detected for any of the three homoeologs of TaGA2ox11; TaGA2ox-B12 and TaGA2ox-B13 were not tested. TaGA2ox-D2, -B3, -D4 and -D10 also further oxidised the GA_51_ product of GA_9_ to its catabolite, which is almost certainly derived by rearrangement of the ketone, 2-oxo-GA_9_, formed by a second round of oxidation at C-2. In contrast to most species, however, we found that some of the wheat enzymes showed markedly reduced substrate specificity towards C_20_- or C_19_-GAs. Notably, TaGA2ox-B3, TaGA2ox-D4 and TaGA2ox-D10, all from GA2ox Class I by phylogeny (Fig. 2C), efficiently converted GA_12_ to GA_110_ (2β-hydroxy-GA_12_) while TaGA2ox-D6, a Class III enzyme, converted GA_9_ to GA_51_ (2β-hydroxy-GA_9_) (Additional file 1: Figure S3). TaGA2ox9, also in Class III, showed partial activity against GA_9_, producing an unidentified product with a retention time different to both GA_51_ and its catabolite.

### Transcript levels for GA biosynthetic and signalling gene expression in wheat tissues by RNA-seq

**Figure 5:**
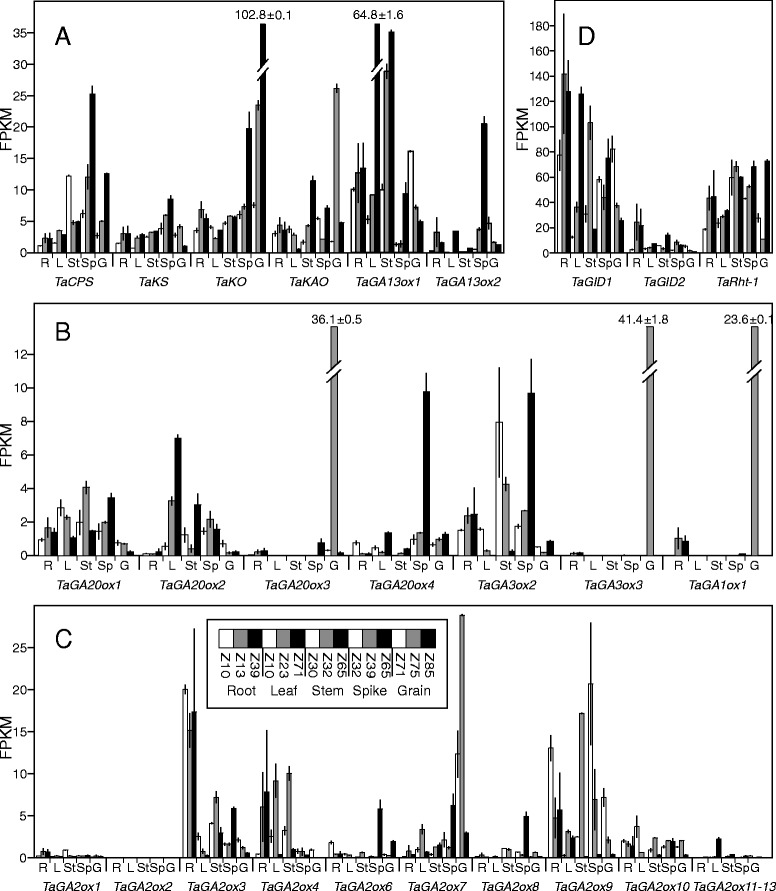
Expression of GA biosynthetic and signalling genes in five bread wheat tissues each at three developmental stages. Expression levels from each homolog were summed and then averaged across replicates, ± standard errors. A: Early GA pathway genes; B: GA-biosynthetic genes; C: GA-inactivating genes; D: GA signalling genes. GA2ox11-13 represents the sum of all additional paralogs of TaGA2ox6: GA2ox11,-12, and -13. FPKM: Fragments per kb per million mapped reads; Z: Zadoks developmental stage

To determine the relative expression levels of the wheat GA genes across the life cycle of wheat, we exploited a dataset of RNA-seq samples derived from five different organs (root, leaf, stem, spike, and grain) each at three developmental stages of bread wheat cv. Chinese Spring, generated as part of the analysis of chromosome 3B [49]. The paired-end RNA-seq reads were mapped to a transcriptome reference consisting of all the wheat coding sequences identified above together with non-redundant cDNA sequences from the IWGSC wheat chromosome arm survey (see Methods). Mean fragments per kb per million mapped reads (FPKM) values (from biological duplicates) for each homoeolog of each gene are presented in Additional file 3 and histograms of expression levels of each gene family, summing the FPKM values from each homoeolog, are shown in Fig. 5.

The genes encoding the early enzymes in GA biosynthesis, catalysing the steps from GGDP to GA_53_, were found to be expressed in all tissues and stages (Fig. 5A and Additional file 3), although the homoeologous gene sets for *TaCPS*, *TaKS*, *TaKO* and *TaKAO* were more highly expressed in the spike at anthesis than in most other tissues. The *TaKO* genes, particularly *TaKO-D1*, also appear to be very highly expressed late in developing grain at Zadoks stage 85 (Additional file 3), although the physiological basis for this is unclear. GA13ox, which catalyses the 13-hydroxylation of GA_12_ to form GA_53_, is encoded by two paralogs as in rice [50]. The homoeologues of *TaGA13ox1* are more highly expressed than those of *TaGA13ox2* in all tissues except mature spikes (Fig. 5A and Additional file 3) and in most organs the *GA13ox* genes have their highest levels of expression in the most mature developmental stage sampled.

The biosynthetic 2-ODD gene families, GA20ox and GA3ox, showed tissue specific expression profiles (Fig. 5B). *TaGA20ox1* and *TaGA20ox2* were the most highly expressed GA20ox genes in vegetative tissues, while *TaGA20ox4* was highest in the spike at anthesis and *TaGA20ox3* expression was very high in and almost completely restricted to expanding grain. *TaGA20ox1* was the most highly expressed GA20ox in roots, whereas in leaves and stems *TaGA20ox1* and *TaGA20ox2* showed a contrasting pattern, with the former having higher expression early in development of the organ and the latter being higher towards tissue maturity. The tissue specificity of GA3ox paralogs was even more striking: *TaGA3ox2* appeared to be responsible for GA biosynthesis in vegetative and floral organs, while *TaGA1ox-B1* and *TaGA3ox3* were expressed at a very high level and almost exclusively at the mid-way stage of grain development, although *TaGA1ox-B1*also showed some expression in roots (Fig. 5B).

The GA2ox gene family, responsible for GA inactivation, showed variation in expression between both genes and tissue/time points (Fig. 5C). Several genes had very low or undetectable expression: no transcripts from *TaGA2ox2* homoeologs were found in any tissue and *TaGA2ox1* was expressed at very low levels. The only transcripts detected from the novel wheat paralogs related to *TaGA2ox6* were found in mature leaves, from *TaGA2ox-B12* (Fig. 5C and Additional file 3). *TaGA2ox3*, *TaGA2ox4* and *TaGA2ox9* were the most highly expressed *GA2ox* genes overall, contributing most to *GA2ox* levels in roots, leaves and stems, while *TaGA2ox9* was the most highly expressed *GA2ox* in the developing spike. In addition, several other *GA2ox* genes, namely *TaGA2ox3*, *TaGA2ox6*, *TaGA2ox7* and *TaGA2ox8*, also contributed to *GA2ox* transcript levels in the spike at anthesis (Fig. 5C and Additional file 3). *TaGA2ox7* was by far the most highly expressed *GA2ox* in developing grain, with some contribution from *TaGA2ox9* at the earliest stage of grain development.

We also investigated expression of the GA signalling components *TaRht*, *TaGID1* and *TaGID2*: these genes were expressed in all tissues studied (Fig. 5D). The genes encoding the GA receptor, *TaGID1*, were most highly expressed in roots, mature leaves and elongating stems while *TaGID2* expression was highest in roots. *TaRht* showed relatively little variation in expression except for a lower level in expanding grain.

There were some noticeable differences between expression levels of homoeologs for each of the studied genes, as shown in Additional file 3. In general, where one homoeolog was expressed at a significantly higher or lower level than the other two homoeologs, this was reflected across most of the 15 tissue/time samples, as has recently been described for homoeologous gene expression in grain tissues [51]. However, there were clear exceptions to this rule: for example, *TaKO-A1* was most highly expressed in nearly all vegetative, spike and grain samples, but *TaKO-D1* dominated in late grain development. Similarly, *TaCPS-A1* showed the lowest level of expression in most vegetative tissues, but was higher in most reproductive tissues.

### qRT-PCR of GA genes in tissues of durum wheat

To support the expression patterns determined by RNA-seq analysis in bread wheat we developed qRT-PCR assays for all the biosynthetic and signalling genes (Additional file 4). As we did not have access to the tissue samples used for the RNA-seq analysis, we assessed transcript levels in existing samples of RNA from shoot, root, grain and spike tissues in the tetraploid (durum) wheat cv. Kronos [34]. Redundant primers were designed to amplify both A and B homoeologous copies of each gene where both sequences were known. Additional file 1 (Figure S4) shows the distribution of expression of each paralog between tissues. The results of this analysis broadly reflect the RNA-seq analysis above: in particular, *TaGA20ox3*, *TaGA1ox1* and *TaGA3ox3* transcripts were detected almost exclusively in grain tissues. *TaGA20ox1* and *TaGA20ox2* were expressed in vegetative tissues, mainly the shoot, although appreciable levels of *TaGA20ox4* were also found in this tissue as well as in the spike. As with the RNA-seq analysis *TaGA3ox2* was expressed in root, shoot and spike tissues but at a much lower level in grain. There was also reasonably good agreement in the expression profiles of the GA2ox genes: the qRT-PCR showed *TaGA2ox3* expression to be highest in roots, *TaGA2ox4* highest in shoots, *TaGA2ox7* highest in grain, while *TaGA2ox8* and *TaGA2ox9* expression was highest in the spike.

### Expression patterns of Brachypodium GA genes

To gain insight into conservation of expression patterns between members of the grass family, we accessed RNA-seq reads from *Brachypodium distachyon* Bd21 [52] and mapped them to the Brachypodium reference transcriptome from Ensembl Plants (http://plants.ensembl.org/). This revealed a high degree of conservation of expression pattern between this species and wheat (Additional file 1: Figure S5): as in wheat, *BdGA20ox1* and *BdGA20ox2* were most highly expressed in vegetative tissues, while *BdGA20ox3* expression was highest in developing seed. Notably, *BdGA20ox4* expression was highly expressed in anthers, suggesting that the high level of *TaGA20ox4* expression observed in wheat spikes close to anthesis (Fig. 5B) may also have been in this organ. Also, while *BdGA3ox2a* expression was confined to vegetative tissues, anthers and developing embryos, *BdGA3ox2b* was expressed predominantly in developing seed tissues, specifically the endosperm (Additional file 1: Figure S5B). The expression profiles of the *GA2ox* gene family were somewhat less well conserved between Brachypodium and wheat, although *GA2ox3* and *GA2ox7* were major contributors in both species. The highest level of *GA2ox* expression was in the pistil (*BdGA2ox3* and *BdGA2ox7*), anther (*BdGA2ox3* and *BdGA2ox8*) and in the embryo (*BdGA2ox8* and *BdGA2ox10*) and endosperm (*BdGA2ox7*) of developing seeds (Additional file 1: Figure S5C).

### GA gene expression patterns in developing wheat grain

**Figure 6:**
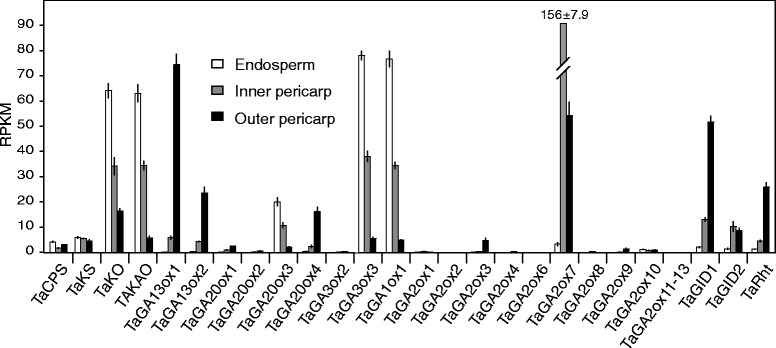
Expression of GA biosynthetic and signalling genes in different layers of the developing bread wheat grain at 10 days post anthesis. Expression levels from each homolog were summed and averaged across replicates, ± standard error. Tissue layers are as defined in Additional File 1 (Figure S6). GA2ox11-13 represents the sum of all additional paralogs of TaGA2ox6: GA2ox11, -12, and -13. RPKM: Reads per kb per million mapped reads

The above results demonstrated that the developing wheat grain exhibits high expression levels of GA biosynthetic and signalling genes, including the novel GA 1-oxidase *TaGA1ox-B1*. To investigate further these patterns of expression within the expanding grain, we generated a set of RNA-seq reads from tissue layers of developing grains of bread wheat cv. Holdfast at 12 days post-anthesis: the endosperm, the inner seed coat/pericarp layer (consisting of the aleurone, nucellar epidermis, integuments, any remaining tube cells, and cross cells) and the outer pericarp layer of immature grain, consisting of the mesocarp parenchyma, hypodermis and epidermis (Additional file 1: Figure S6A). These tissue distributions were confirmed using known cell-specific gene expression patterns (Additional file 1: Figure S6B), although it is likely that there is some cross-contamination of the samples from adjacent layers. Single-end RNA-seq reads were mapped to the wheat transcriptome reference described above (see Methods), and average RPKM (reads per kilobase per million mapped reads) values for each gene from three biological replicates were calculated. The results in Fig. 6 represent combined counts from A, B and D homoeologs, while expression values for individual genes are presented in Additional file 5.

Taken together, RPKM expression values validated those of the whole grain sample from the previous experiments, showing that the early GA biosynthetic genes *TaKO* and *TaKAO* were highly expressed in the grain and that *TaGA20ox3, TaGA20ox4, TaGA3ox3,* and *TaGA2ox7* account for the majority of GA 20-oxidase, GA 3-oxidase, and GA 2-oxidase expression, respectively. *TaGA1ox-B1* was also highly expressed in grain. The three GA signalling genes assayed, *TaGID1, TaGID2* and *TaRht,* were all expressed in the grain. However, there were very clear distinctions in expression levels of these genes between different grain tissue layers.

While the cyclase genes, *TaCPS* and *TaKS*, were expressed in all three grain layers, *TaKO* and *TaKAO* were more highly expressed in the endosperm and inner pericarp than in the outer layer. In stark contrast, transcripts of both paralogs of *TaGA13ox* were absent from the endosperm and concentrated in the outer pericarp. The later GA biosynthetic genes *TaGA20ox3* and *TaGA3ox3*, and also the novel *TaGA1ox* gene, were found to be more highly expressed in the endosperm than in the seed coat/pericarp layers and, within the seed coat/pericarp, more highly in the inner layer than the outer layer (Fig. 6). *TaGA20ox4* was an exception to this trend, showing highest levels of expression in the outer pericarp layers. A contrasting trend was observed for the GA catabolic gene *TaGA2ox7*, which was expressed at negligible levels in the endosperm, but at higher levels in the seed coat/pericarp tissues and highest in the inner layer (Fig. 6). The GA signalling genes *TaGID1*, *TaGID2* and *TaRht* were expressed almost exclusively in the seed coat/pericarp layers, almost no expression being detected in the endosperm. *TaGID1* and *TaRht* were also predominantly expressed in the outer of the two seed coat/pericarp layers.

## Discussion

### 2-Oxoglutarate-dependent dioxygenases in the GA pathway of wheat

Since GAs contribute to nearly every stage of plant development, modifying their biosynthesis, perception and turnover has great potential to engineer improved varieties of agriculturally important crops. A prerequisite for such an approach is a more complete description and characterization of the components of the GA biosynthesis and signalling pathways. In the current study, we describe the sequence and biochemical activity of the *GA20ox*, *GA3ox* and *GA2ox* gene families in wheat, building upon a previous study which described the genes encoding the enzymes catalysing the earlier stages of GA biosynthesis in wheat [24]. While the absence of a fully-sequenced wheat genome precluded a precise determination of the final number of genes in each family, the transcript and genome assemblies used for this study cover a large proportion of the wheat genes [32, 34]. For missing or incomplete genes, we also successfully reassembled chromosome arm-specific sequence reads onto consensus sequences derived from assembly of 454 reads, allowing us to further extend the coverage, and also illustrating the potential for improving the wheat genome assembly using existing short read sequence data. Moreover, the number of 2-ODD paralogs found in wheat are very similar to those described for both rice and Brachypodium that have completely sequenced genomes, and in most cases we identified full-length or partial sequences for all three homoeologs, suggesting that the great majority of the genes from the GA pathway were identified in this study.

We identified or assembled full-length or partial sequences for all three homoeologs of four *GA20ox* genes in wheat (Table 1), corresponding to the four characterized members of this family in Brachypodium and rice (Fig. 2A). We confirmed that all four paralogs encode fully functional GA 20-oxidase enzymes, each capable of catalysing the complete series of reactions from GA_12_ to GA_9_. We similarly identified wheat homologs of all but one of the ten characterized rice *GA2ox* genes and confirmed that representative homoeologs of each of the enzymes encoded by these genes have GA 2-oxidase activity against either C_19_- or C_20_-GAs as substrates. However, these assays revealed that the substrate specificities of some of the wheat GA2ox enzymes do not conform to the current observations in other species where enzymes in Classes I and II, structurally-related to *Arabidopsis* AtGA2ox1 through AtGA2ox6, are active against C_19_-GAs whereas enzymes related to AtGA2ox7 and AtGA2ox8 (i.e., from Class III; Fig. 2C) are active only against C_20_-GAs [48]. Previously, the only identified exception to this pattern was the Class I enzyme CsGA2ox4 from cucumber, which showed weak activity against the C_20_-GA GA_12_ [53]. However, this study shows that several of the wheat enzymes have broader substrate specificities: TaGA2ox-B3, TaGA2ox-D4 and TaGA2ox-D10, all from GA2ox Class I by sequence similarity (Fig. 2C), were also active against C_20_-GAs, while TaGA2ox-D6, a Class III enzyme, was active against C_19_-GAs (Additional file 1: Figure S3). The significance of this wider substrate specificity is unclear, particularly since there do not appear to be distinct biological roles for the three structural classes of GA2ox enzymes or distinct class-specific expression profiles during development [21].

In addition to the *GA2ox* genes in rice, Brachypodium and barley, we identified five additional *GA2ox* gene sequences in wheat, comprising one homoeologous group on the long arm of the group 4 chromosomes, *TaGA2ox11*, plus two more individual paralogs on chromosome 4B, *TaGA2ox-B12* and *TaGA2ox-B13.* All of these additional genes are closely related to *TaGA2ox6*, suggestive of gene duplication events in the ancestors of wheat. The importance of these GA 2-oxidase genes to wheat development remains unclear, however, since expression of all three homoeologs of *TaGA2ox11* was negligible in all tissues studied and none of the encoded enzymes could be shown to exhibit GA 2-oxidase activity. Expression of the *TaGA2ox-B12* and *TaGA2ox-B13* paralogs was similarly very low in the assayed tissues.

Of the three 2-ODD families, the largest divergence from rice was observed in the GA 3-oxidases. The rice genome contains two *GA3ox* genes [54]; the wheat genes orthologous to *OsGA3ox2* were identified and characterized in a previous study and shown to act principally as GA 3-oxidases, but also with additional minor activities including GA 2-oxidase, 2,3-desaturation and even 13-hydroxylation [29]. We did not identify a clear wheat ortholog of *OsGA3ox1*, either in sequence homology or in syntenic position, and this gene was also absent from the Brachypodium genome. Instead, during the current study, we found novel *GA3ox* gene sequences in bread wheat, three of which were closely related to one another and most likely represent a homoeologous group (*TaGA3ox3*) while a fourth gene, *TaGA1ox-B1* (initially named *TaGA3ox-B4*) was identified only in the B genome (Fig. 2B); orthologs of these genes were found in durum wheat but not in rice or Brachypodium. Of the *GA3ox3* group, only *TaGA3ox-B3* could be shown to encode an active GA 3-oxidase, converting GA_9_ to GA_4_; *TaGA3ox-D3* contains a 7 bp insertion in the second exon and while *TaGA3ox-A4* appeared to encode a full-length protein, no enzyme activity could be detected in the *E. coli* expression products. This might be explained by polymorphisms observed in this paralog that result in mis-sense changes in amino acid residues conserved between all grass GA3ox sequences (Additional file 1: Figure S9), but technical problems with heterologous expression cannot be ruled out.

In contrast, expression products of *TaGA3ox-B4* were found to catalyse 1β-hydroxylation of GA_9_ to yield GA_61_ (Figs. 3B and 4), rather than the 3β-hydroxylation suggested by its similarity to other GA 3-oxidases. This is the first description of an enzyme with GA 1-oxidase activity, and the gene was therefore renamed as *TaGA1ox-B1.* We found a single ortholog of *TaGA1ox-B1* in durum wheat but not in the A genome or the A and D diploid progenitors, and little sequence data is available for *Ae. speltoides*, the closest living relative of the B genome progenitor. However, a search of the draft barley genome [37] identified two GA3ox-like genes: *HvGA3ox2* was shown to encode a ‘normal’ GA 3-oxidase (Fig. 3D) and had 95-96 % nucleotide sequence identity with the three wheat *TaGA3ox2* homoeologs. In contrast, *HvGA3ox1* [39] is most closely related to *TaGA1ox-B1* (87.6 % nucleotide sequence identity; Fig. 2B) and is located on the orthologous chromosome arm in barley, 2HL. We showed that *HvGA3ox1* encodes a GA-3β,18-dihydroxylase, converting GA_9_ to GA_131_ (Figs. 3D and 4) and was therefore renamed as *HvGA3,18ox1*.

Analysis of the POPSEQ mapping data [32] showed that both *TaGA3ox-A3* and *TaGA1ox1-B1* were in orthologous positions on the group 2 chromosomes of wheat to *HvGA3,18ox1* on barley chromosome 2H. However, these genes are not orthologous to either *OsGA3ox1* or *OsGA3ox2*, which are located on rice chromosomes 5 and 1, respectively, or to the two *GA3ox2* variants in Brachypodium, where are located on chromosomes 2 and 4 from that species. It seems highly likely, therefore, that the three *Triticeae*-specific genes evolved from an unidentified common ancestor but have subsequently diverged, acquiring different catalytic activities. Amino acid sequence identity ranged from 66 % between HvGA3,18ox1 and TaGA3ox-A3 to 79 % between HvGA3,18ox1 and TaGA1ox1-B1, whereas amino acid sequence identity between the wheat and barley GA3ox2 enzymes responsible for bioactive GA production in vegetative tissues is 95–96 %. This suggests the existence of a strong purifying selection of mutations in the *GA3ox2* genes compared with the genes of novel function identified here.

Investigation of the expression patterns of these novel *GA3ox*-like genes showed that *TaGA1ox-B1* is only expressed in the developing grain of both bread and durum wheat (Fig. 5 and Additional file 1: Figure S4), predominantly in the endosperm (Fig. 6) and that *TaGA3ox-B3* shows a similar expression profile, although with some expression detected in vegetative tissues; the other homoeologues of *TaGA3ox3* are also expressed in developing endosperm, but at a much lower level (Additional file 5). Similarly, inspection of the barley Gene Expression Atlas at http://www.plexdb.org revealed that *HvGA3,18ox1* (Affymetrix probeset contig9888_at) is only expressed in the endosperm of developing barley grains. Supporting the expression and functional analyses described above, developing wheat grains accumulate 1β-hydroxylated GAs, including GA_54_ (1β-hydroxy-GA_4_) and GA_55_ (1β-hydroxy-GA_1_) [42]. Given that we showed that *TaGA1ox-B1* is active against GA_9_ but not GA_4_, whereas *TaGA3ox-B3* is active against both GA_9_ and GA_61_ (1β-hydroxy-GA_9_), the order of reaction *in planta* is likely to be GA_9_ → GA_61_ → GA_54_, catalysed by TaGA1ox-B1 and TaGA3ox-B3, respectively (Fig. 4), and it seems likely that these two enzymes also produce the 13-hydroxylated equivalent, GA_55_, from GA_20_ in developing grain via the same sequence of reactions. In contrast, 1-hydroxy-GAs have not been identified in developing barley grains, which accumulate a number of 18-hydroxylated GAs, including GA_131_ (18-hydroxy-GA_4_) [44], presumably produced through the action of HvGA3,18ox1 on GA_9_ (Fig. 4).

### Tissue specificity of GA biosynthesis, signalling and turnover in wheat

Our survey of GA gene expression in five tissues of bread wheat each at three stages of development (Fig. 5) and also different tissues of durum wheat (Additional file 1: Figure S4) and Brachypodium (Additional file 1: Figure S5) indicated that all organs and time points were competent in GA biosynthesis, perception and inactivation, exhibiting measurable transcript levels for at least one paralog of each component assayed. One caveat to consider when drawing conclusions from GA biosynthetic expression data is the existence of feedback and feed-forward mechanisms which act upon the transcription of 2-ODD genes. As part of a homeostatic mechanism, increased concentrations of bioactive GA inhibit *GA20ox* and *GA3ox* expression and promote *GA2ox* expression [23]. Therefore, high expression of GA biosynthetic genes is not necessarily indicative of higher endogenous bioactive GA levels and may instead result from their up-regulation under conditions of low GA. The early biosynthetic genes, *TaCPS*, *TaKS*, *TaKO* and *TaKAO*, and the signalling genes *TaRht*, *TaGID1* and *TaGID2*, are expressed at all stages of development, albeit with marked variation in transcript levels between tissues and stages. However, it is clear that the multiple paralogs of the 2-ODD genes encoding GA20ox, GA3ox and GA2ox have more specific patterns of expression. In particular, the expression of *TaGA20ox3*, *TaGA3ox3* and *TaGA1ox1* is very high in the grain, specifically at Zadoks 75 developmental stage, but is much lower in the other tissues assayed. This is similar to the expression patterns observed for *OsGA20ox3* and *OsGA3ox1* in rice [55], although no wheat ortholog of *OsGA3ox1* was identified; *OsGA20ox3* and *TaGA20ox3* are similarly not orthologous by chromosome location, although the phylogenetic relationship appears clear (Fig. 2A).

In the leaf and stem, *TaGA20ox1* is expressed more highly in early, rapidly-elongating stages of development while *TaGA20ox2* appears to be highest in more mature tissue, after the period of maximal elongation growth (Fig. 5B), implying that *TaGA20ox1* might be more important in determining growth rate and therefore final organ size. However, both paralogs of these two genes contribute to plant height in rice [16,56], although only *OsGA20ox2* (*SD-1*) has been exploited in plant breeding [57]. *TaGA20ox1*, *-2* and *-3* are also expressed to some extent at all stages of spike development, a result consistent with a recent study which showed that these three GA biosynthetic genes are up-regulated by long days in the shoot apical meristem of wheat, where bioactive GA is required for the up-regulation of floral meristem identity genes *SOC1-1* and *LFY* in spike development [7]. Relatively few genes from the *GA2ox* family show high levels of expression in the tissues assayed, and some have narrow expression domains. Thus *TaGA2ox3*, *TaGA2ox4* and *TaGA2ox9* account for most of the vegetative GA2ox expression, while *TaGA2ox6* and *TaGA2ox8* predominate in the flowering spike and *TaGA2ox7* in developing grain.

Analysis of GA gene expression in separate tissue layers of the developing grain revealed that the GA biosynthetic genes, notably *TaKO*, *TaGA20ox3*, *TaGA3ox3* and *TaGA1ox1,* were predominantly expressed in the endosperm. In contrast, genes involved in GA perception, signalling and turnover were expressed at only low levels in the endosperm and were instead expressed at their highest levels in the inner and outer seed coat/pericarp layers. Indeed, expression levels of the GA receptor, *TaGID1*, and the central signalling component, *TaRht*, were 26- and 22-fold higher, respectively, in the outer pericarp than in the endosperm (Fig. 6). These results suggest that the main site of GA biosynthesis in the young developing grain is the endosperm tissue while GA signalling occurs predominantly in the outer layers of the wheat grain, possibly implying movement of bioactive GAs between the tissues. As this period of grain development, Zadoks 73, is one of rapid radial expansion growth, it is tempting to speculate that bioactive GA produced by the endosperm promotes cell expansion in the outer grain layers which otherwise would constrain growth of the endosperm. In support of this, dwarfing mutations in *TaRht* genes that reduce sensitivity to bioactive GAs also reduce grain size [15], implying a role for GAs and GA signalling in grain expansion.

However, no defined role for GA in seed development has been demonstrated in any species. Although developing seeds of plants often accumulate high levels of GAs, in many cases these are inactive or partially active forms. Thus, in rice the *OsGA3ox1* gene that is expressed in developing grain encodes an enzyme with both 3β-hydroxylase and 2β-hydroxylase activities [54], although the latter activity is a minor component, at least when assayed *in vitro*. Unlike the other sequenced grass species (maize, sorghum and *Setaria italica*), wheat, barley and Brachypodium lack orthologs of *GA3ox1* but in each case have evolved novel paralogs of the *GA3ox2* gene responsible for GA biosynthesis in vegetative tissues (Fig. 2B and Additional file 1: Figure S7) that have high expression in developing grain. In the case of bread wheat, we show above that two novel paralogs, *TaGA3ox3* and *TaGA1ox1*, combine to produce the unusual gibberellin GA_54_ (1β-hydroxy-GA_4_) in grain. Similarly, in barley *HvGA3,18ox1* encodes a bifunctional GA 3β,18-dihydroxylase; this gene is closely related to *TaGA3ox3* and *TaGA1ox1* and its expression is confined to developing grain. It is presumably, therefore, responsible for the accumulation of 18-hydroxy-GAs, such as GA_131_ (18-hydroxy-GA_4_), in seeds of this species. Brachypodium also contains a paralogous gene related to *GA3ox2*, *Bradi4g23570*, that we have named *BdGA3ox2b*. This gene is similarly expressed exclusively in seed tissues (Additional file 1: Figure S5) and encodes an enzyme with only GA 3-oxidase activity. Thus, rice, barley, wheat, and Brachypodium have independently evolved paralogs of *GA3ox* that produce high levels of GAs in developing grain. However, the GAs synthesized in wheat and barley grain exhibit reduced biological activity: GA_131_ is ~4-fold less active than GA_4_ [58] and the biological activity of 1-hydroxy GAs is similarly lower than of their non-1-hydroxylated equivalents (personal communication from the late Dr. John Lenton). The biological relevance of these unusual GA-modifying activities is therefore unclear and merits further investigation.

### Target genes for novel dwarfing effects in wheat

The introduction of the *Rht* dwarfing alleles during the Green Revolution had a major impact on global wheat productivity because of a reduced incidence of lodging and improved assimilate partitioning [59]. However, because *Rht* is a negative regulator of GA signalling in all tissues, these alleles are also associated with negative pleiotropic effects, such as the requirement for planting closer to the surface and a slight reduction in grain size [15, 19]. Results from our study confirm that *Rht* was expressed in all tissues assayed, including the outer layers of expanding grain and suggests that alternative dwarfing alleles, more specific and targeted to the stem, may limit these negative pleiotropic effects. Several genetic studies to identify plant height QTLs are being carried out in order to identify alternative dwarfing alleles for wheat, although to date no specific gene has been identified [60].

One solution may lie in the selection of loss-of-function mutations in GA biosynthetic genes using currently available TILLING populations [61, 62]. In rice, the most widely used dwarfing gene is *GA20ox2* [16, 17], where multiple loss-of-function alleles have been identified which reduce height. These mutations have only limited effects on reproductive development, presumably because other *GA 20-oxidase* genes regulate GA biosynthesis in these tissues. In wheat, *TaGA20ox1*, *TaGA20ox2* and *TaGA20ox4* are expressed in the leaf and stem, suggesting that these are promising candidates in which to search for disruptive mutations. However, since all three homoeologs of both *TaGA20ox1* and *TaGA20ox2* are expressed in stem tissues (Additional file 3) multiple mutations may necessary to obtain economically relevant height reductions. Current efforts to sequence the exomes of wheat TILLING lines [63] is accelerating the discovery of mutations for most of the genes presented in this study, which will facilitate the experimental validation of their function and the testing of their effects on plant height and productivity.

## Conclusions

In this study we identified the genes encoding 2-ODD enzymes from the GA biosynthetic pathway of bread wheat, through a combination of interrogation of publicly available, partially-assembled genome sequences and *de novo* assembly of shotgun reads from individual chromosome arms. We also catalogued the GA 2-ODD genes from barley and Brachypodium. With a few exceptions, hexaploid bread wheat contains three homoeologs of each paralog of the genes identified in rice. In each case the function of the enzymes encoded by the genes was demonstrated by heterologous expression and their expression was analysed across a range of tissues and developmental stages. Certain paralogs of the *GA20ox*, *GA3ox* and *GA2ox* gene families were shown to be exclusively expressed in developing grain, including a novel, highly-expressed GA 1-oxidase gene in wheat endosperm (and a related GA 3,18-dihydroxylase gene in barley). The presence of these genes likely explains the high levels of 1β-hydroxylated GAs in wheat grain, and 18-hydroxylated GAs in barley, in both cases the activities resulting in a reduction in the biological activity of the GAs produced. The pattern of transcript accumulation of the GA genes in early developing grain suggested that, while most GA biosynthesis is carried out in the endosperm, GA inactivation, perception and signalling are confined to the seed coat and pericarp, consistent with a role for GA in grain expansion. This comprehensive identification and characterisation of the GA 2-ODD genes in wheat will provide the basis for a better understanding of GA-regulated development in this species, including the involvement of GA biosynthesis and signalling in grain development.

## Methods

### Identification and phylogeny of wheat genes for components of the GA pathway

To identify wheat (*Triticum aestivum* L.) genes encoding components of the GA biosynthetic and signalling pathway, sequences from Brachypodium (*Brachypodium distachyon* L.), identified by BLASTP at www.phytozome.org using rice (*Oryza sativa* L.) peptide sequences [31] as queries, were used to search the IWGSC chromosome arm survey assembly at urgi.versailles.inra.fr. In many cases it was not possible to identify all three wheat homoeologs and, therefore, guided assembly of homologous genomic sequence reads from chromosome-arm-specific shotgun sequencing assembled to 454 contigs [32, 33] was carried out using the Geneious software (Biomatters Ltd) as outlined in Additional file 1 (Figure S1). Accession numbers for all full-length 2-ODD sequences identified from wheat and other grasses are shown in Additional file 2. In addition, full-length genomic, coding sequence and polypeptide sequences of all the wheat 2-ODD genes, plus coding sequences of other genes in the pathway that were used for transcript analysis, are provided as FASTA files in Additional file 6.

For phylogenetic analysis, peptide sequences were aligned using MUSCLE [64] and the resulting alignments (Additional file 1: Figures S8-S10) were edited to remove unaligned sequences in the N- and C-terminal regions. For GA20ox the N-terminal 82 and C-terminal 31 columns of the alignment were removed; similarly for GA3ox the 39 N-terminal and C-terminal 45 columns and for GA2ox the N-terminal 69 and C-terminal 49 columns were deleted. Phylogenetic analysis was carried out on the resulting alignments using the PhyML algorithm within TOPALi v2.5 [65], including model optimisation and bootstrapping (100 repeats); an outgroup was used for rooting and phylogenetic trees were drawn using MEGA5 [66]. The trees obtained using Neighbor Joining methods were not significantly different. To simplify presentation, only one homoeolog of each paralogous bread wheat gene was included in the phylogenetic trees presented in Figure 2, but complete versions of each tree with bootstrap values are shown in Additional file 1 (Figures S11–S13).

### Heterologous expression of wheat, barley and Brachypodium GA 2-ODDs

Coding sequences for a single representative homoeolog of each wheat 2-ODD, and of barley (*Hordeum vulgare* L.) and Brachypodium (*Brachypodium distachyon* L.) *GA3ox* genes, from the GA pathway were synthesised (Genscript, Piscataway, USA) and inserted in-frame as a fusion with thioredoxin in the expression vector pET32b. The constructs were expressed in *E. coli* strain Rosetta2 (DE3) pLysS (Merck Millipore Ltd) by induction of log phase cultures with 0.5 mM IPTG followed by growth at 25 °C for 5–6 h. Cells were harvested by centrifugation, suspended in 100 mM Tris-Cl pH7.5, 5 mM DTT, 10 u.ml^-1^ DNAseI (Sigma), lysed by freeze-thaw and sonication and assayed using radiolabelled GA substrates in the presence of co-substrates and co-factors as described previously [67]. Control assays were also run to demonstrate that neither *E. coli* proteins nor products encoded by the pET32b vector were active against the GA_12_ and GA_9_ substrates. All products were identified by comparison of HPLC retention times with those of standards, or, in the case of ^14^C-labelled GA_54_, GA_61_ and GA_131_, by comparison of their mass spectra with published spectra [40] after combined gas chromatography–mass spectrometry of methyl esters trimethylsilyl ethers as described previously [68] using a MAT95XP mass spectrometer coupled to a Trace GC (ThermoElectron).

### Mapping RNA-seq reads to the wheat reference transcriptome

Paired-end RNA-seq reads from root, leaf, stem, spike and grain tissues of wheat cv. Chinese Spring [49] were obtained from http://urgi.versailles.inra.fr/. Paired-end RNA-seq reads from Brachypodium tissues were downloaded from the Short Read Archive at Genbank (accession number SRA046377). For developing grain samples, bread wheat cv. Holdfast seedlings were vernalized for 8 weeks at 8 °C and transplanted to pots of Rothamsted Prescription Mix including slow-release fertilizer. The plants were grown in a glasshouse with supplemental lighting under a 16 h day (18 °C), 8 h (14 °C) night and tagged at anthesis (anther emergence). Immature grain at 12 days post-anthesis were dissected into endosperm, inner and outer seed coat/pericarp tissues and RNA extracted as described by Wan et al. [69]. Illumina RNA-seq libraries from three biological replicates of each tissue were prepared using the TruSeq mRNA sample kit (Illumina TruSeq RNA sample preparation guide Part 15008136 Rev A, Nov 2010) using 4 μg total RNA. Single end reads, 110 bp, were generated on an Illumina GAIIx sequencer and RNA-seq analysis was carried out within the Galaxy environment [70]. For all sets of RNA-seq data, sequencing reads were trimmed for quality using Trimmomatic [71] and reads from each library were mapped using BWA-mem using default parameters [72] to a wheat reference transcriptome consisting of the predicted cDNA sequences from the IWGSC assembly (v21) at www.plants.ensembl.org/Triticum_aestivum. Where genes of interest were absent from this reference, full-length coding sequences developed in this work were added to replace either partial or missing sequences as detailed in Additional file 7. Gene sequences from the early part of the GA pathway were obtained from online databases: *TaCPS* [EMBL:GU980886, EMBL:GU980887, EMBL:GU980888); *TaKS* [GenBank:FR719731, EMBL: GU980889, EMBL: GU980890], *TaKO* [EMBL:GU980893, EMBL: GU980894, EMBL: GU980895]; *TaKAO* [EMBL:GU980891, EMBL:GU980892, EMBL:GU143912]. FPKM (for paired-end reads) and RPKM (for single-end reads) values were generated using the eXpress tool [73].

### Availability of supporting data

The RNA-seq data from tissue layers of developing grain described in this work are available in the ArrayExpress database (www.ebi.ac.uk/arrayexpress) under accession number E-MTAB-3103. The phylogenetic input data and analysis for the GA20ox, GA3ox and GA2ox trees are available from Dryas (doi:10.5061/dryad.sk2nd).

## Additional files

Additional file 1: Figure S1. Strategy for identification and assembly of wheat GA 2-ODD sequences. **Figure S2:** HPLC traces of incubations of heterologous expression products of GA20ox genes with [1-^14^C]-GA_12_. Figure S3: HPLC traces of incubations of heterologous expression products of GA2ox genes with [1-^14^C]-GA_12_ and [1-^14^C]-GA_9_. Figure S4: Relative expression of GA biosynthetic and signalling genes in durum wheat tissues determined by qRT-PCR. Figure S5: Transcript analysis of GA biosynthetic and signalling genes in *Brachypodium distachyon* by RNA-seq. Figure S6: Dissection of tissue layers from developing grain of bread wheat. Figure S7: Phylogenetic tree of grass GA3ox-like protein sequences. Figures S8–10: GA20ox, GA3ox and GA2ox protein alignments, respectively. Figures S11–13: Phylogenetic trees of all GA20ox, GA3ox and GA2ox protein sequences, respectively, with bootstrap support values.

Additional file 2: **Rice, Brachypodium, barley and wheat genes encoding GA 2-ODDs including accession numbers.**

Additional file 3: **Homoeolog-specific expression of GA biosynthetic and signalling genes across five tissues of wheat at three developmental stages.**

Additional file 4: **qRT-PCR primers used in this study and qRT-PCR methods.**

Additional file 5: **Homoeolog-specific expression of GA biosynthetic and signalling genes in different layers of the wheat grain.**

Additional file 6: **FASTA files containing genomic, coding sequence and polypeptide sequences of all full-length wheat genes described in this work.**

Additional file 7: **Sequences in the IWGSC reference used for RNA-seq mapping substituted with full-length CDS sequences from this work.**

## Abbreviations

2-ODD: 2-oxoglutarate-dependent dioxygenases
FPKM: Fragments per kilobase per million mapped reads
GA: Gibberellin
GA1ox: GA 1-oxidase (GA 1β-hydroxylase)
GA13ox: GA 13-hydroxylase
GA20ox: GA 20-oxidase
GA2ox: GA 2-oxidase (GA 2β-hydroxylase)
GA3ox: GA 3-oxidase (GA 3β-hydroxylase)
GC-MS: Coupled gas chromatography–mass spectrometry
HPLC: High performance liquid chromatography
IWGSC: International Wheat Genome Sequencing Consortium
RPKM: Reads per kilobase per million mapped reads
qRT-PCR: Quantitative reverse transcription-polymerase chain reaction
TILLING: Targeting induced local lesions in genomes
