# Supplementary material for: Heterologous expression and transcript analysis of gibberellin biosynthetic genes of grasses reveals novel functionality in the GA3ox family
Source: BMC Plant Biol. 2015 Jun 5;15:130. doi: 10.1186/s12870-015-0520-7 (PMC4455330; doi:10.1186/s12870-015-0520-7)
Supplement: Supplementary file 1 — Strategy for identification and assembly of wheat GA 2-ODD sequences. Figure S2: HPLC traces of incubations of heterologous expression products of GA20ox genes with [1-14C]-GA12. Figure S3: HPLC traces of incubations of heterologous expression products of GA2ox genes with [1-14C]-GA12 and [1-14C]-GA9. Figure S4: Relative expression of GA biosynthetic and signalling genes in durum wheat tissues determined by qRT-PCR. Figure S5: Transcript analysis of GA biosynthetic and signalling genes in Brachypodium distachyon by RNA-seq. Figure S6: Dissection of tissue layers from developing grain of bread wheat. Figure S7: Phylogenetic tree of grass GA3ox-like protein sequences. Figures S8–10: GA20ox, GA3ox and GA2ox protein alignments, respectively. Figures S11–13: Phylogenetic trees of all GA20ox, GA3ox and GA2ox protein sequences, respectively, with bootstrap support values. [file 12870_2015_520_MOESM1_ESM.pdf]

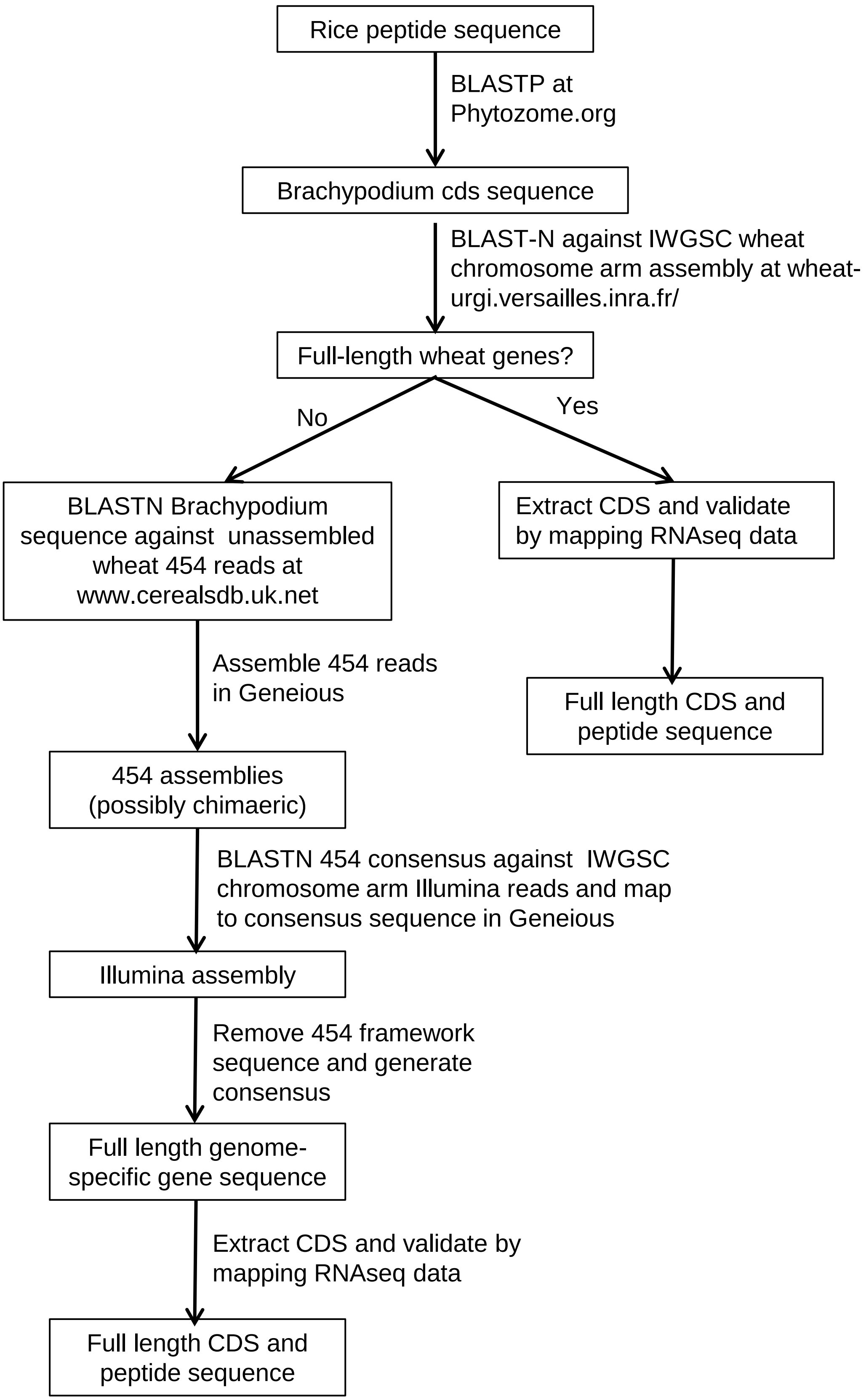

Figure S1: Workflow for identification and assembly of genes for wheat GA 2-ODDs

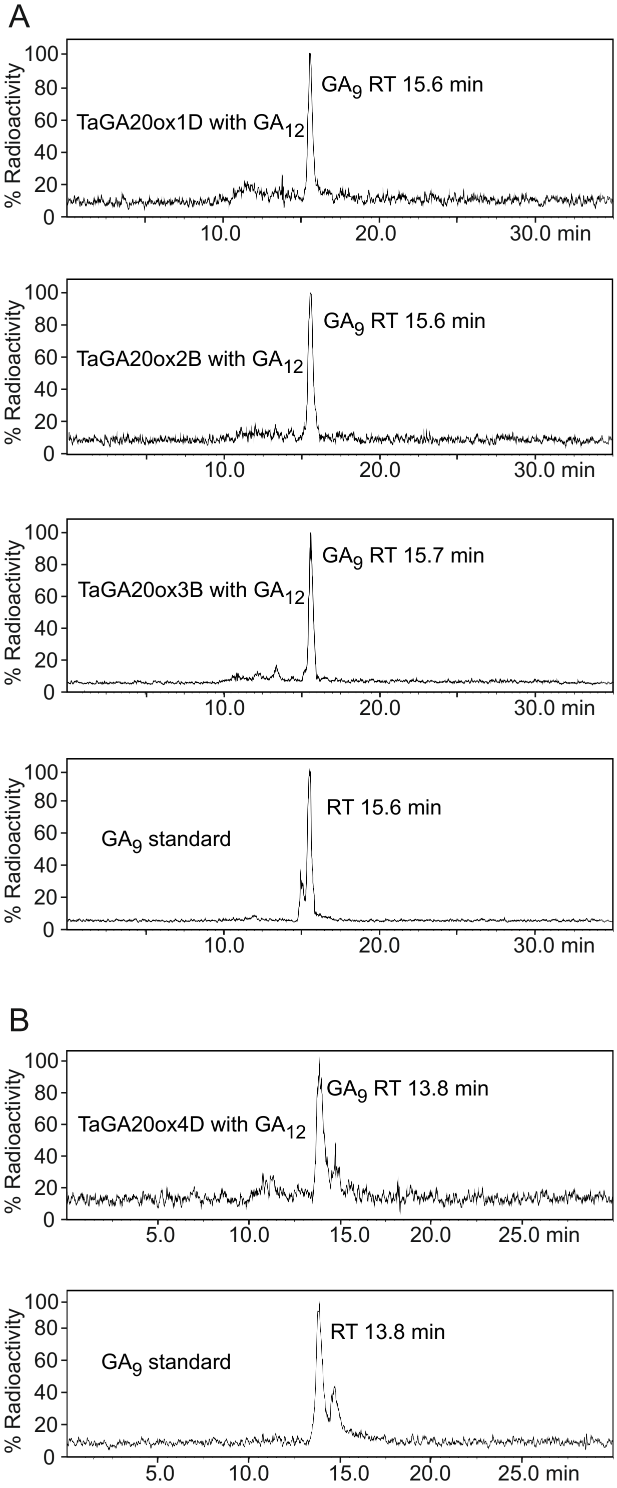

Figure S2. HPLC traces of incubations of heterologous expression products of GA20ox genes with $[^{14}C_1]GA_{12}$.

A: TaGA20ox1D, TaGA20ox2B and TaGA20ox3B. B: TaGA20ox4D - note that this sample was run under different HPLC conditions and the GAs therefore have different retention times than in A. The X-axis shows HPLC retention time in minutes while the Y-axis is scaled such that the height of the largest peak of radioactivity is 100%.

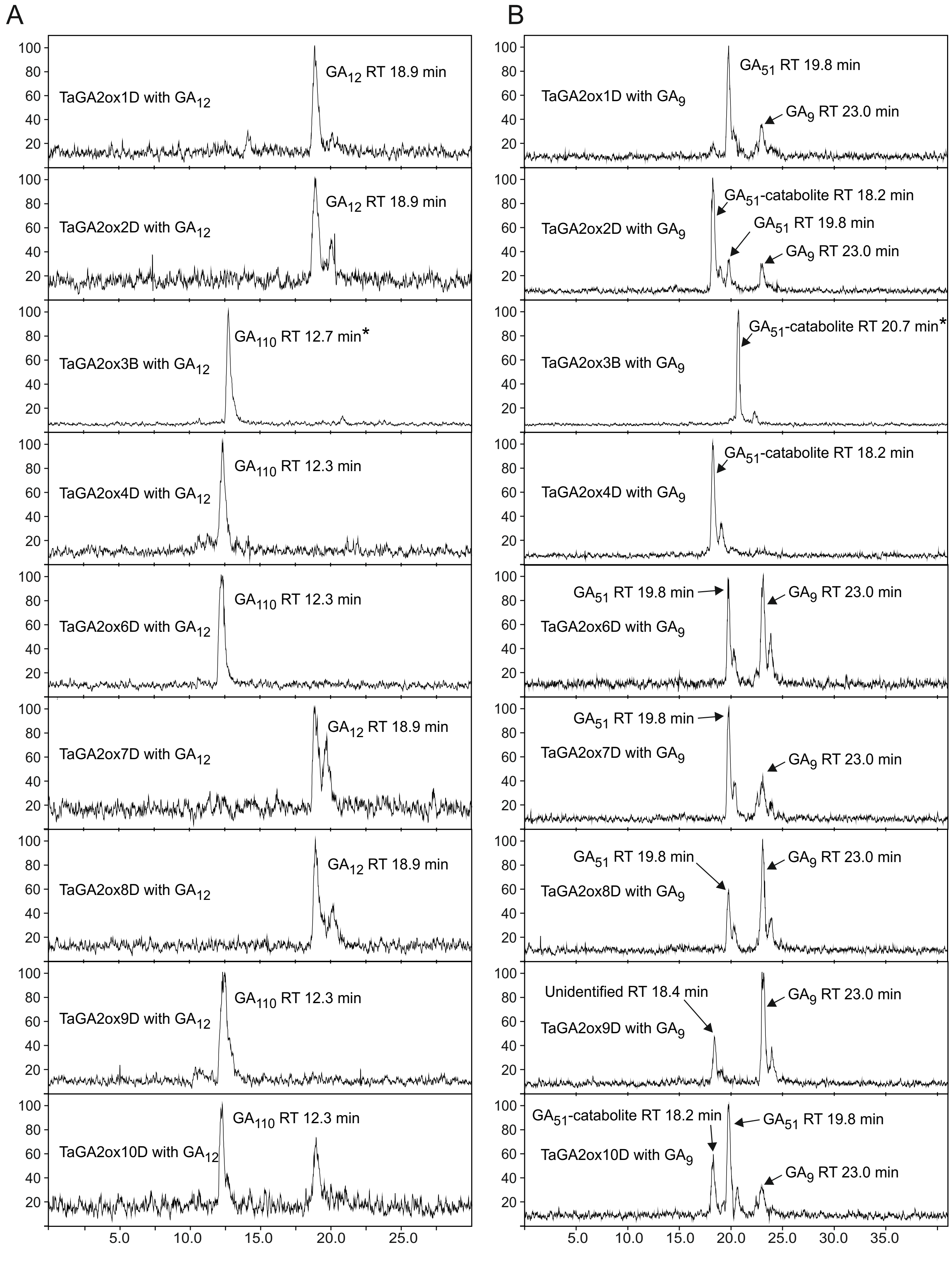

Figure S3. HPLC traces of incubations of heterologous expression products of GA2ox genes with (A) [$^{14}C_1$]GA_{12} and (B) [$^{14}C_1$]GA_9. The Y-axis shows retention time in minutes. *The products from incubation of TaGA2ox3B with [$^{14}C_1$]GA_9 were run under different HPLC conditions and have a longer retention time than in other panels. The X-axis shows HPLC retention time in minutes while the Y-axis is scaled such that the height of the largest peak of radioactivity is 100%.

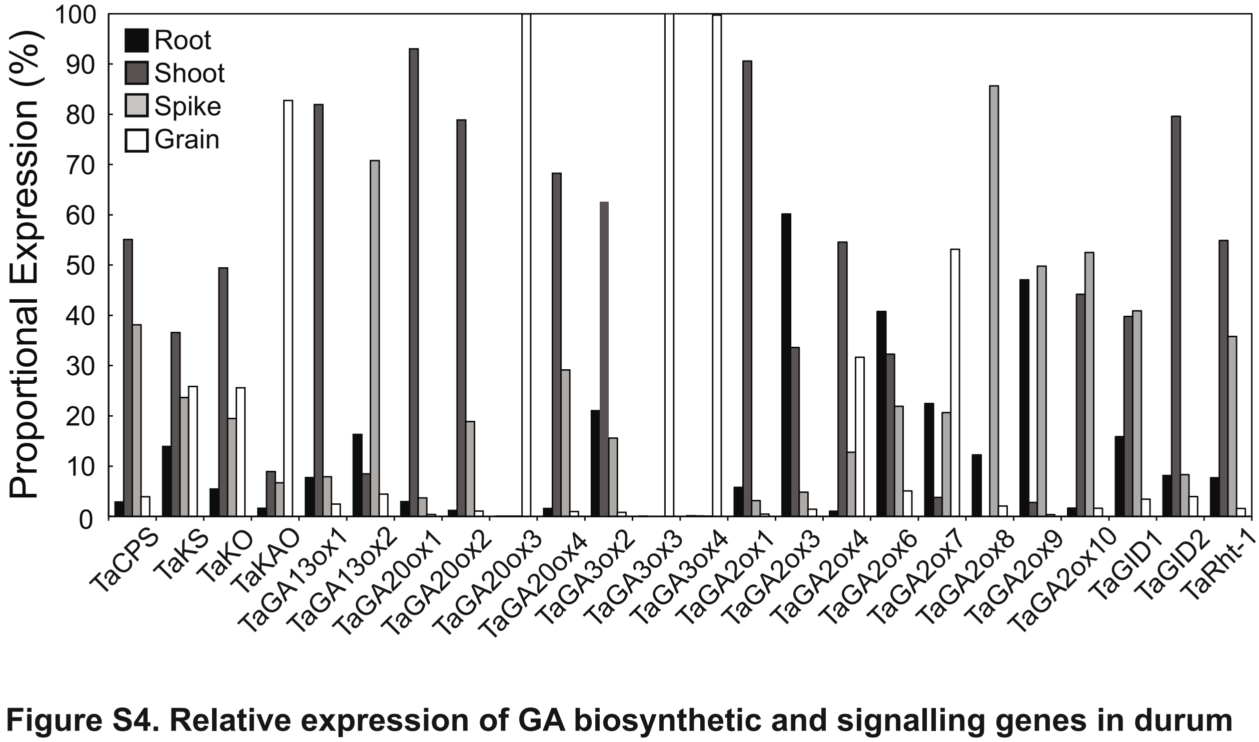

Figure S4. Relative expression of GA biosynthetic and signalling genes in durum wheat tissues determined by RT-PCR. Bars show the proportional distribution of transcripts from each gene across the four tissues.

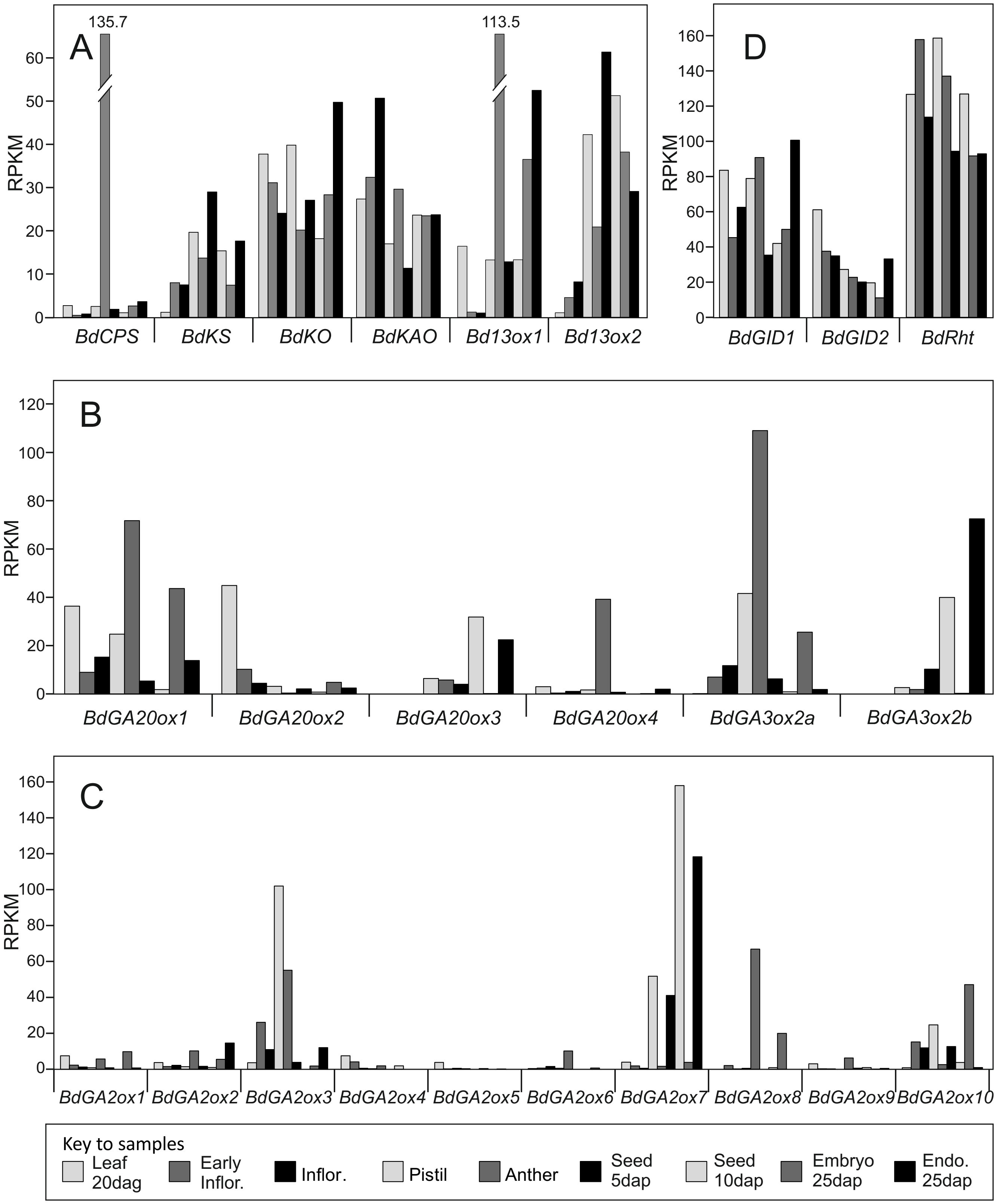

Figure S5. Transcript analysis of GA biosynthetic and signalling genes in *Brachypodium distachyon* by RNA-seq.
Unreplicated RNA-seq read samples from Brachypodium tissues was obtained from the Short Read Archive (http://www.ncbi.nlm.nih.gov/sra). Mapping to the Brachypodium reference transcriptome (http://plants.ensembl.org/) and calculation of RPKM values were carried out using BWA-mem and eXpress as described in Methods. dag: days after germination; dap: days after pollination; inflor: infloresence; endo.: endosperm.

A

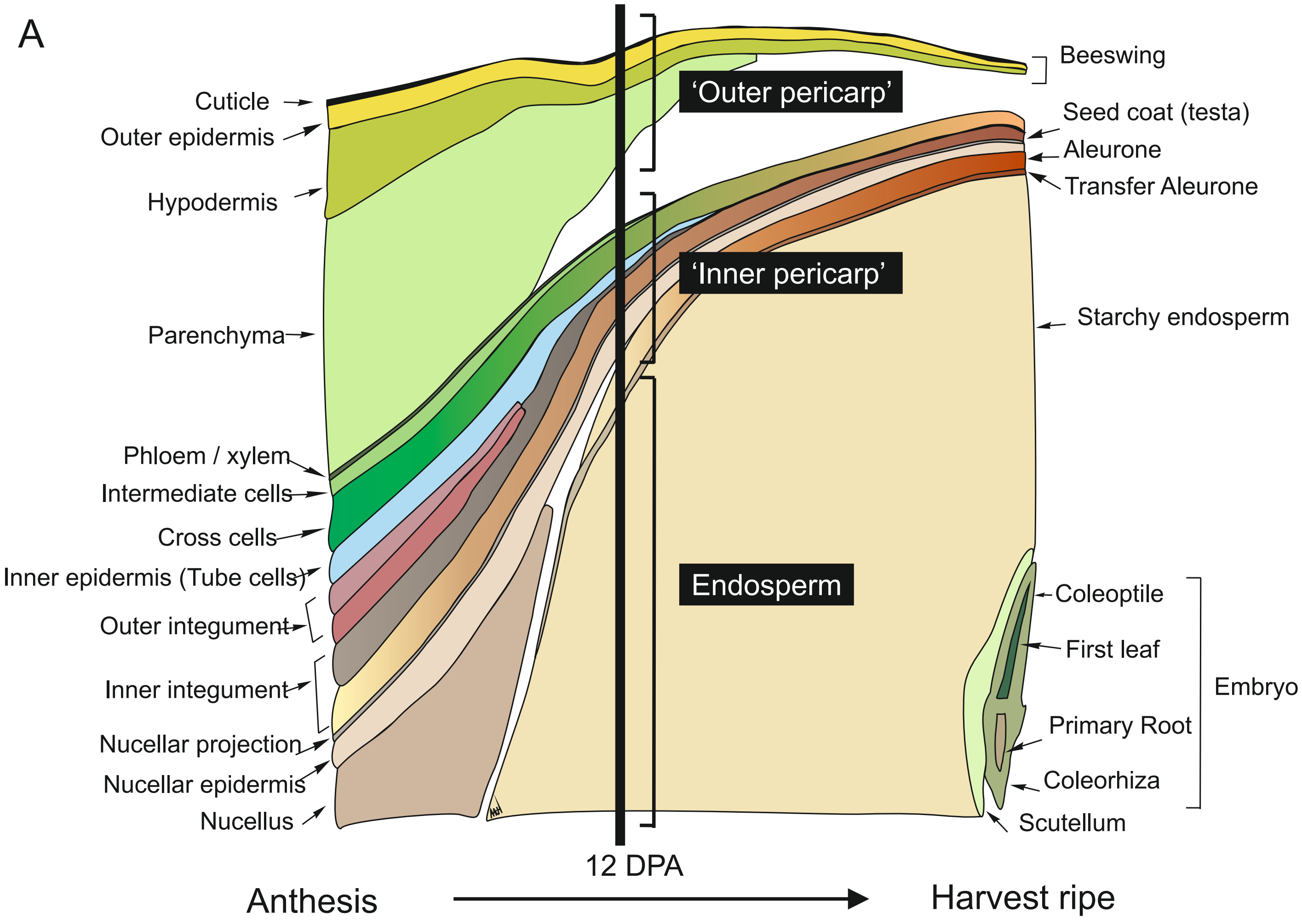

B

IWGSC gene model	Gene name	Known location	Ref
Traes_5DS_0BFE4FD87.1	PinB	Endosperm	1
Traes_1DL_D861501F5.1	HMW-1Dx5	Endosperm	2
Traes_1AS_9385680A1.1	END1	Transfer aleurone	3
Traes_4AL_3D862C090.1	7s-globulin	Aleurone & transfer cells	4
Traes_2BS_CADAA49C9.1	Per-1	Aleurone	5
Traes_5AL_320913F7A.1	Ltp2	Aleurone	6
Traes_2AL_2C0155C8F.1	Nucellain	Nucellus	7
Traes_1AL_6E5BA9C6F.1	CAB	Cross cells	8
Traes_5AL_9C5A73F10.1	Rubisco-SSU	Cross cells	8
Traes_6AS_6AAA7678C.1	Yabby-DL	Outer pericarp	9

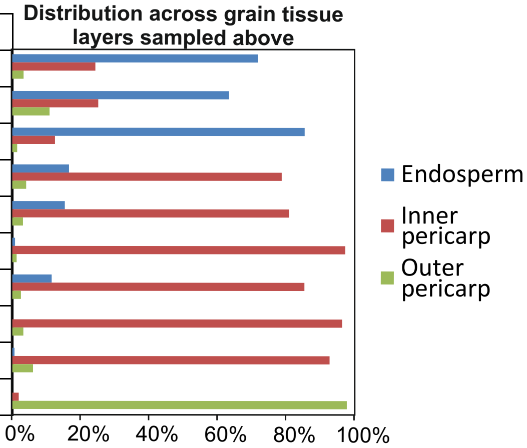

Figure S6. Dissection of tissue layers from developing grain of bread wheat. A: Diagrammatic representation of changes in the tissue layers of developing wheat grain with time. Samples for RNA-seq analysis were taken at 12 days post-anthesis (DPA), approximately equivalent to Zadoks stage 73. B: The tissue layers dissected from developing wheat grain were assessed by the distribution of mean FPKM values of RNA-seq reads corresponding to transcripts of known location. References: (1) Gautier et al. (1994) Plant Mol Biol 25, 43-57, (2) Lamacchia et al. (2001) J Exp Bot 52, 243-250, (3) Doan et al. (1996) Plant Mol Biol 31, 877-886, (4) Huttly et al., unpublished, (5) Stacy et al. (1996) Plant Mol Biol 31, 1205-1216, (6) Kalla et al. (1994) Plant J 6, 849-860, (7) Linnestad et al. (1998) Plant Physiol 118, 1169-1180, (8) Morrison (1976) Bot Gaz 137, 85-93, (9) Wan et al. (2008) BMC Genomics 9.

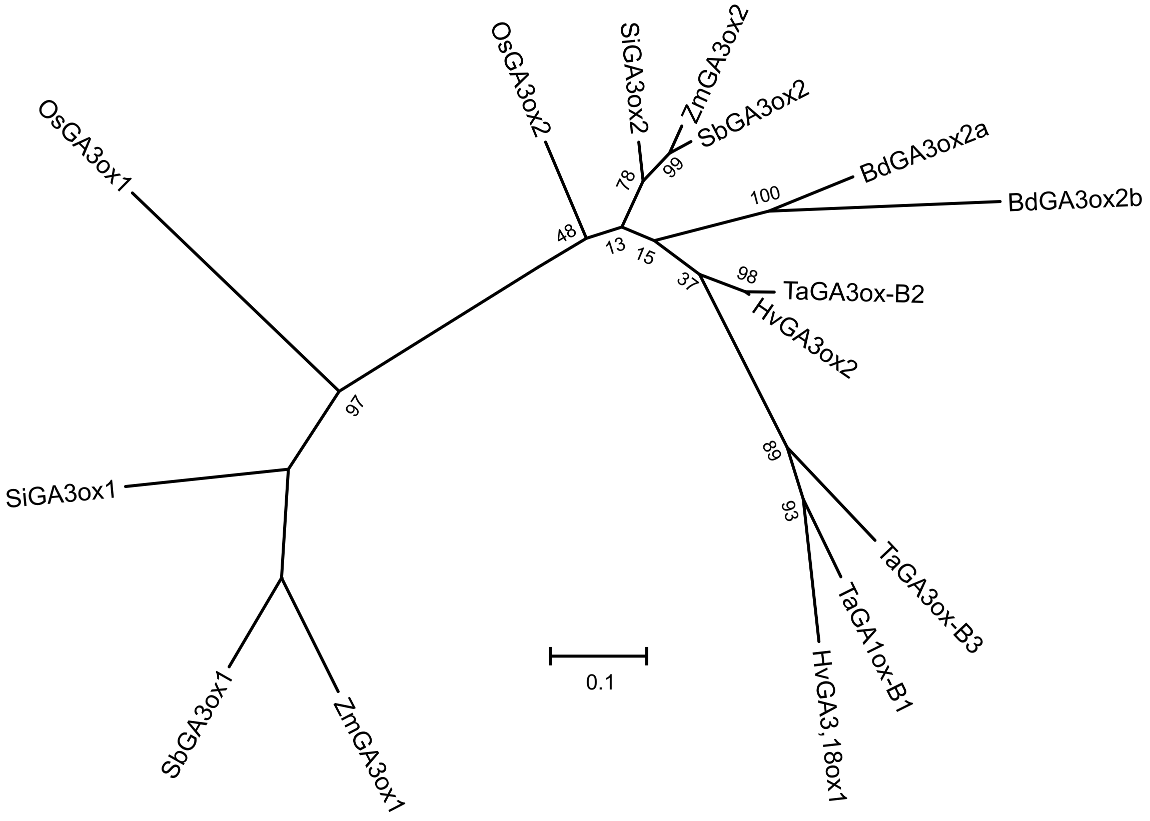

Figure S7. Phylogenetic tree of grass GA3ox-like protein sequences.
In addition to the wheat (Ta), Barley (Hv), Brachypodium (Bd) and rice (Os) sequences described in the text, the tree also contains GA3ox sequences from maize (ZmGA3ox1: GRMZM2G044358; ZmGA3ox2: GRMZM2G036340), sorghum (SbGA3ox1: Sb09g005400; SbGA3ox2: Sb03g004020) and *Setaria italica* (SiGA3ox1: Si025127m; SiGA3ox2: Si001832m); accession codes are from www.phytozome.org. Numbers show bootstrap support; the scale bar shows substitutions per site. Non-aligned residues were trimmed from the alignment prior to tree generation using PhyML within TOPALi, as reported in Materials and Methods.

Figure S8. Alignment of GA20ox protein sequences using MUSCLE with OsGA3ox2 as outgroup.

```
                       *        20         *        40         *        60         *        80
AtGA20ox1   : -----------MAVSFVTTSPEEEDKPKLGLGNIQTPLIFNP-------------------------------SMLNLQA :  38
AtGA20ox2   : ---------MAILCTTTSPAEKEHEPKQDLEKDQTSPLIFNP-------------------------------SLLNLQS :  40
AtGA20ox3   : ---------------------------MATECIATVPQIFSE-------------------NKTKEDSSIFDAKLLNQHS :  34
AtGA20ox4   : ----------------MECIIKLPQRFNKNKSKKNPLRIFDS-------------------------------TVLNHQP :  33
AtGA20ox5   : --------------------MCIYASRQTVCPYLTPFKVKRP-------------------KSREMNSSDVNFSLLQSQP :  41
BdGA20ox1   : --------------------------------MVMQPVLFDA-------------------------------AVLSGRS :  17
BdGA20ox2   : -------------------------------MDTAPPLVLSP-----------SAPSITDTSTAKAKQSAAPVFDLRREP :  38
BdGA20ox3   : -----------------------------------MALEFDA-------------------------------TILSRAP :  14
BdGA20ox4   : --------MVDYQLSNSGEPNATTPIAAMDQVGRNPLLLFGP-----------------------ADDSKNAIIPWRQSK :  49
HvGA20ox1   : ----------------------------------MVQPVFDA-------------------------------AVLSGRT :  15
HvGA20ox2   : -MVLQTAQQEPSLTRPPRCSVASAGSPAAMDTSPATPLLLQP-------PAPSIDPSAAKAAVSKGGGAATAVYDLRREP :  72
HvGA20ox3   : ----------------------------------MASLVFDA-------------------------------AVLSRKE :  15
HvGA20ox4   : ---------MVDVSNFVEANGNAAVSIPAMEVAGSPHVPFVP----------------RDANATDSKNAKDVLDLWRQQK :  55
OsGA20ox1   : ---------------------------MSMVVQQEQEVVFDA-------------------------------AVLSGQT :  22
OsGa20ox2   : ----------------------------MVAEHPTPPQPHQP------------PPMDSTAGSGIAAPAAAAVCDLRMEP :  40
OsGA20ox3   : -----------------------------------AAVVFDA-------------------------------AILSKQE :  14
OsGA20ox4   : MHASPHPLQIAHDTLLLSLTHTLCTAETGSTIRTGTAMVYISNAQDASKLIVTAKGGGGEADDAAASSAAVVLDLWRQPA :  80
TaGA20ox-A1 : ----------------------------------MVRPVFDA-------------------------------AVLSGRS :  15
TaGA20ox-B1 : ----------------------------------MVQPVFDA-------------------------------AVLSGRA :  15
TaGA20ox-D1 : ----------------------------------MVQPVFDA-------------------------------AVLSGRA :  15
TaGA20ox-A2 : -----------------------------MDTSPATPLLLQP-------PAPSIDPFAAKAAVNKNGGAATAVYDLRREP :  44
TaGA20ox-B2 : -----------------------------MDTSPTTPLLLQP-------PAPSIDPFAAKAAVNKNGGAATAVYDLRREP :  44
TaGA20ox-D2 : -----------------------------MDTSPATPLLLQP-------PAPSIDPFAAKAAVNKGGGAATAVYDLRREP :  44
TaGA20ox-A3 : ----------------------------------MATLVFDA-------------------------------AVLSRKD :  15
TaGA20ox-B3 : ----------------------------------MATLVFDA-------------------------------AVLSRKD :  15
TaGA20ox-D3 : ----------------------------------MATLVFDA-------------------------------AVRSRKD :  15
TaGA20ox-A4 : -----------------------------MEVARSPHLPCVP----------------VDANATDSNSATDVLDLWRQQK :  35
TaGA20ox-B4 : -----------------------------MEVARSPHLPYVP----------------GDANATDSKNATDVLDLWRQQK :  35
TaGA20ox-D4 : -----------------------------MEVARSPHLPCVP----------------VDANATDSNSATDVLDLWRQQK :  35
OsGA3ox2    : --------------------------------MPTPSHLKNP-----------------------------LCFDFRAAR :  19

                       *       100         *       120         *       140         *       160
AtGA20ox1   : N-IPNQFIWPD-DEKPSIN---VLE---LDVPLIDLQNLLS------D-PSSTLDASRLISEACKKHGFFLVVNHGISEE : 103
AtGA20ox2   : Q-IPNQFIWPD-EEKPSID---IPE---LNVPFIDLSS-----------QDSTLEAPRVIAEACTKHGFFLVVNHGVSES : 101
AtGA20ox3   : HHIPQQFVWPD-HEKPSTD---VQP---LQVPLIDLAGFLS-----GD-SCLASEATRLVSKAATKHGFFLITNHGVDES : 101
AtGA20ox4   : DHIPQEFVWPD-HEKPSKN---VPI---LQVPVIDLAGFLS-----ND-PLLVSEAERLVSEAAKKHGFFLVTNHGVDER : 100
AtGA20ox5   : N-VPAEFFWPE-KD-VAPS---EGD---LDLPIIDLSGFLN-----GN-EAETQLAAKAVKKACMAHGTFLVVNHGFKSG : 106
BdGA20ox1   : D-IPSQFIWPA-DESPTPD--AAEP---LDVPLIDIGGIVAS--GAGDRAAAVASVARLVGDACSRHGFFQVVNHGIDAA :  88
BdGA20ox2   : K-IPAPFVWPH-AE-VRPT--TAEE---LGMPVVDVGVLRKSRNGGGD-AAALRRAVAQVAAAGATHGFFQVSGHGVDAA : 109
BdGA20ox3   : V-IPPQFVWPA-DE-ASPA--AVGE---IAIPVIDLAAFLS--------GSGDFSGADHFAAACERHGFFQIVNHGVDPA :  78
BdGA20ox4   : Q-IPDSFVWPH-ADTHPPSSSTTTTTELLDVPVVDLAAALR------D-AAGMRDAAAQAAAACASHGFFLVTGHGVDPA : 120
HvGA20ox1   : D-IPSQFIWPE-GESPTPD--ATEE---MHVPLIDIGGMLS-----GD-PRAAAEVTRLVGEACERHGFFQVVNHGIDAQ :  82
HvGA20ox2   : K-IPAPFVWPH-AE-VRPT--TAQE---LAVPVVDVGVLRN-----GD-AAGIRRAVAQVASACATHGFFQVSGHGVDNA : 138
HvGA20ox3   : D-IPPQFIWPA-DEAPSVD--GVEE---IVVPVVDLAGFLA-----GD-DAGL----NELVAACERHGFFQVVNHGVDPA :  78
HvGA20ox4   : Q-IPAPFIWPH-AD-ARPS--SILE---LDVPVVDIGAALHS-------AAGMARAAAQVAEACASHGFFQVTGHGVDPA : 120
OsGA20ox1   : E-IPSQFIWPA-EESPGSV--AVEE---LEVALIDVGA-----------GAERSSVVRQVGEACERHGFFLVVNHGIEAA :  84
OsGa20ox2   : K-IPEPFVWPN-GD-ARPA--SAAE---LDMPVVDVGVLRD-----GD-AEGLRRAAAQVAAACATHGFFQVSEHGVDAA : 106
OsGA20ox3   : A-IPAQFVWPA-DEAPAADDGVVEE---IAIPVVDLAAFLA--------SGGIG---RDVAEACERHGFFQVVNHGVDPA :  78
OsGA20ox4   : K-IPAPFVWPR-ADVALPP--SSPPTGELDVPVVDLAAALR------D-AAGMRRAVAQVAAACASHGFFQVSGHGVPPS : 149
TaGA20ox-A1 : D-IPSQFIWPE-GESPTPD--AAEE---LHVPLINIGGMLS-----GD-AAAAAEVTRLVGEACERHGFFQVVNHGIDAE :  82
TaGA20ox-B1 : D-IPSQFIWPE-GESPTPD--AAEE---LHVPLIDIGGMLS-----GD-AAAAAEVTRLVGEACERHGFFQVVNHGIDAE :  82
TaGA20ox-D1 : D-IPSQFIWPE-GESPTPD--AAEE---LHVPLIDIGGMLS-----GD-PAAAAEVTRLVGEACERHGFFQVVNHGIDAE :  82
TaGA20ox-A2 : K-IPAPFVWPH-AE-VRPT--TAEE---LAVPVVDVGVLRN-----GD-AAGLRRAVAQVAAACATHGFFQVSGHGVDDA : 110
TaGA20ox-B2 : K-IPAPFVWPH-AE-VRPT--TAEE---LAMPVVDVSVLRN-----GD-AAGLRRAVAQVAAACATHGFFQVSGHGVDDT : 110
TaGA20ox-D2 : K-IPAPFVWPH-AE-VRPT--TAQE---LAVPVVDVGVLRN-----GD-AAGLRRAVAQVAAACATHGFFQVSGHGVDEA : 110
TaGA20ox-A3 : D-IPPQFVWPA-DEAPSVH--GVEE---IAVPVVDIAGFLA-----GD--GAATGGLRDLAAACEKHGFFQVVNHGVDPA :  81
TaGA20ox-B3 : D-IPPQFVWPA-DEAPSVD--GVEE---IAVPVVDIAGFLA-----GDGSAGAGPGLRDLAAACEKHGFFQVVNHGVDPA :  83
TaGA20ox-D3 : D-IPPQFVWPA-DEAPSVD--GVEE---IVVPVVDLAGFLA-----GD-GSADAGGLRDLAAACEKHGFFQVVNHGVDPA :  82
TaGA20ox-A4 : Q-IPAPFVWPH-AD-ARPS--SMLE---LDVPVVDIGAALHS-------AAGMGRAAAQVAEACASHGFFQVTGHGVDLA : 100
TaGA20ox-B4 : Q-IPAPFVWPN-AD-ARPS--SMLE---LDVPVVDIGAALHS-------AAGMGRAAAQVAEACASHGFFQVTGHGVDPA : 100
TaGA20ox-D4 : Q-IPAPFVWPN-AD-ARPS--SMLE---LDVPVVDIGAALHS-------AAGMGRAAAQVAEACASHGFFQVTGHGVDPA : 100
OsGA3ox2    : R-VPETHAWPGLDDHPVVD--GGGGGGEDAVPVVDVGA--------GD-------AAARVARAAEQWGAFLLVGHGVPAA :  81
```

```
                             *        180         *        200         *        220         *        240
AtGA20ox1   : LISDAHEYTSRFFDMPLSEKQRVLRKSGESVGYASSFTGRFSTKLPWKETLSFRFC------DDMSRSKSVQDYFCDALG : 177
AtGA20ox2   : LIADAHRLMESFFDMPLAGKQKAQRKPGESCGYASSFTGRFSTKLPWKETLSFQFS------NDNSGSRTVQDYFSDTLG : 175
AtGA20ox3   : LLSRAYLHMDSFFKAPACEKQKAQRKWGESSGYASSFVGRFSSKLPWKETLSFKFS-----PEEKIHSQTVKDFVSKKMG : 176
AtGA20ox4   : LLSTAHKLMDTFFKSPNYEKLKAQRKVGETTGYASSFVGRFKENLPWKETLSFSFS---PTEKSENYSQTVKNYISKTMG : 177
AtGA20ox5   : LAEKALEISSLFFGLSKDEKLRAYRIPGNISGYTAGHSQRFSSNLPWNETLTLAFK--------KGPPHVVEDFLTSRLG : 178
BdGA20ox1   : LLADAHRCVDAFFKLPLAEKQRALRRPGESCGYASSFVGRFSSKLPWKETLSFRSS--------PSCPDLPLDFILSNLG : 160
BdGA20ox2   : LARAALDGASDFFRMPLADKQRARRVQGTVSGYTSAHADRFATKLPWKETLSFGFH----GQDCAEAKPVVVDYFTSTLG : 185
BdGA20ox3   : LLAEAYRSMDAFYALPLAEKQRAKRRLGENHGYAGSFTGRFESRLPWKETMSFNCS------DAPENASKVVDYFVSVLG : 152
BdGA20ox4   : LARAALDGAAGFFRLPLATKQRARRVTGSVAGYAAAHADRFAANLPWKETLSFRHH------HDDDDRDAVLDYFTSTLG : 194
HvGA20ox1   : LLADAHRCVDAFFTMPLPEKQRALRRPGESCGYASSFTGRFASKLPWKETLSFRSC--------PSDPALVVDYIVAPLG : 154
HvGA20ox2   : LARAALDGASGFFRLPLAEKQRARRIPGTVSGYTSAHADRFASKLPWKETLSFGFH-----DRAGAAAPVVVDYFTSTLG : 213
HvGA20ox3   : LLAKAYRCCDAFYALPLAEKQRAQRRLGENHGYAGSFVGRFGSKLPWKETMSFNCS------AAPESARKVVDYFVGVLG : 152
HvGA20ox4   : LAQAALDGAADFFRLPLATKQRARRSPGTVKGYASAHADRFAAKLPWKETLSFIHNHVHEDVGARASSH-VVDYFTSALG : 199
OsGA20ox1   : LLEEAHRCMDAFFTLPLGEKQRAQRRAGESCGYASSFTGRFASKLPWKETLSFRYS----SAGDEEGEEGVGEYLVRKLG : 160
OsGa20ox2   : LARAALDGASDFFRLPLAEKRRARRVPGTVSGYTSAHADRFASKLPWKETLSFGFH-------DRAAAPVVADYFSSTLG : 179
OsGA20ox3   : LLAEAYRCCDAFYARPLAEKQRARRRPGENHGYASSFTGRFDCKLPWKETMSFNCS------AAPGNARMVADYFVDALG : 152
OsGA20ox4   : LARAALDGAAGFFRLPPAAKQRARRAPGTVTGYTAAHADRFVDNLPWKETLSFGHR--HAN-AAGNNSSTVADYF-STLG : 225
TaGA20ox-A1 : LLADAHRCVDNFFTMPLPEKQRALRRPGESCGYASSFTGRFASKLPWKETLSFRSC--------PSDPALVVDYIVATLG : 154
TaGA20ox-B1 : LLADAHRCVDAFFTMPLPEKQRALRRPGESCGYASSFTGRFASKLPWKETLSFRSC--------PSDPALVVDYIVATLG : 154
TaGA20ox-D1 : LLADAHRCVDNFFTMPLPEKQRALRHPGESCGYASSFTGRFASKLPWKETLSFRSC--------PSDPALVVDYIVATLG : 154
TaGA20ox-A2 : LARAALDGASGFFGLPLAEKQRARRVPGTVSGYTSAHADRFASKLPWKETLSFGFH------DRAGAAPVVVDYFTSTLG : 184
TaGA20ox-B2 : LARAALDGACGFFRLPLAEKQRARRIPGTVSGYTSAHADRFASKLPWKETLSFGFH------DRAGAAPVVVDYFTSTLG : 184
TaGA20ox-D2 : LARAALDGASGFFRLPLAEKQRARRVPGTVSGYTSAHADRFASKLPWKETLSFGFH------DRAGAAPVVVDYFTSTLG : 184
TaGA20ox-A3 : LLAKAYRCCDAFYALPLAEKQRAQRRLGENHGYAGSFVGRFGSKLPWKETVSFNCS------AAPEGARKVVDYFVAVLG : 155
TaGA20ox-B3 : LLAKAYQCCDAFYALPLAEKQRAQRRLGENHGYAGSFVGRFGSKLPWKETMSFNCS------AAPEGARKVVDYFVGVLG : 157
TaGA20ox-D3 : LLAKAYRCCDAFYAMPLAEKQRAQRRLGENHGYAGSFVGRFGSKLPWKETMSFNCS------AAPEGARKVVDYFVGVLG : 156
TaGA20ox-A4 : LARAALDGAADFFRLPLATKQRVRRSPGTVEGYASAHADRFAAKLPWKETLSFSHN--HDDVGARGNSHVVVDYFTSALG : 178
TaGA20ox-B4 : LARAALDGAADFFRLPLATKQGARRSPGTVEGYASAHADRFAAKLPWKETLSFSHN--HDDVGARGDSHVVVDYFTSALG : 178
TaGA20ox-D4 : LARAALDGAADFFRLPLATKQGARRSPGTVEGYASAHADRFAAKLPWKETLSFSHN--HDDVGARGNSHVVVDYFTSALG : 178
OsGA3ox2    : LLSRVEERVARVFSLPASEKMRAVRGPGEPCGYGSPPISSFFSKLMWSEGYTFS----------PSSLRSELRRLWPKSG : 151

                             *        260         *        280         *        300         *        320
AtGA20ox1   : HGF-QPFGKVYQEYCEAMSSLSLKIMELLGLSLGV---K-RD--YFREFFEENDSIMRLNYYPPCIKPDLTLGTGPHCDP : 250
AtGA20ox2   : QEF-EQFGKVYQDYCEAMSSLSLKIMELLGLSLGV---N-RD--YFRGFFEENDSIMRLNHYPPCQTPDLTLGTGPHCDP : 248
AtGA20ox3   : DGY-EDFGKVYQEYAEAMNTLSLKIMELLGMSLGV---E-RR--YFKEFFEDSDSIFRLNYYPQCKQPELALGTGPHCDP : 249
AtGA20ox4   : DGY-KDFGSVYQEYAETMSNLSLKIMELLGMSLGI---K-RE--HFREFFEDNESIFRLNYYPKCKQPDLVLGTGPHCDP : 250
AtGA20ox5   : NHR-QEIGQVFQEFCDAMNGLVMDLMELLGISMGL---KDRT--YYRRFFEDGSGIFRCNYYPPCKQPEKALGVGPHNDP : 252
BdGA20ox1   : EEH-RRLGEVYARYCEEMSRVSLEIMEVLGESLGV---G-RS--HYRSFFEGNDSIMRLNYYPPCQRPYETLGTGPHCDP : 233
BdGA20ox2   : QDF-EPMGRVYQEYCEKMKELSLTIMELLELSLGV---E-RG--YYREFFADSSSIMRCNYYPPCPEPERTLGTGPHCDP : 258
BdGA20ox3   : EEY-RQMGEVWQEYCDVMTRLALDVTDLLAVGLGL---G-RG--ALRGFFAGGDSVMRLNNYPPCRQPHLTLGTGPHRDP : 225
BdGA20ox4   : DDF-KPLGEVYQEYCGAMEAASLAIMEVLGVSLGV---G-RG--HYRDFFADGSSVMRCNYYPPCPEPDRTLGTGPHCDP : 267
HvGA20ox1   : EDH-RRLGEVYARYCSEMSRLSLEIMEVLGESLGV---G-RA--HYRRFFEGNESIMRLNYYPPCQRPLETLGTGPHCDP : 227
HvGA20ox2   : PDY-EPMGRVYQEYCGKMKELSLRIMELLELSLGV---EKRG--YYRDFFADSSSIMRCNYYPPCPEPERTLGTGPHCDP : 287
HvGA20ox3   : EEY-RHMGDVWQEYCNEMTRLALDVTEVLAACLGL---D-RG--ALRGFFAGDDSLMRLNHYPPCKKPHLTLGTGPHHDP : 225
HvGA20ox4   : DDF-MHLGEVYQEYCEAMEDASLAIMEVLGVSLGL---G-RG--YYRDFFADGSSIMRCNYYPRCPEPDRTLGTGPHCDP : 272
OsGA20ox1   : AEHGRRLGEVYSRYCHEMSRLSLELMEVLGESLGIVGDR-RH--YFRRFFQRNDSIMRLNYYPACQRPLDTLGTGPHCDP : 237
OsGa20ox2   : PDF-APMGRVYQKYCEEMKELSLTIMELLELSLGV---E-RG--YYREFFADSSSIMRCNYYPPCPEPERTLGTGPHCDP : 252
OsGA20ox3   : EEY-RHMGEVYQEYCDVMTRLALDVTEVLAVALGL---G-RG--ELRGFFADGDPVMRLNHYPPCRQPHLTLGTGPHRDP : 225
OsGA20ox4   : DDF-KHLGEVYQEYCEAMEEVTKAIMAVLGESLGV---G-GG--YYREFFEDSSSIMRCNYYPPCPEPERTLGTGPHCDP : 298
TaGA20ox-A1 : EDH-RRLGEVYARYCSEMSRLSLEIMEVLGESLGV---G-RA--HYRRFFEGNDSIMRLNYYPPCQRPLETLGTGPHCDP : 227
TaGA20ox-B1 : EDH-RRLGEVYARYCSEMSRLSLEIMEVLGESLGV---G-RA--HYRRFFEGNDSIMRLNYYPPCQRPMETLGTGPHCDP : 227
TaGA20ox-D1 : EDH-RRLGEVYARYCSEMSRLSLEIMEVLGESLGV---G-RA--HYRRFFEGNDSIMRLNYYPPCQRPLETLGTGPHCDP : 227
TaGA20ox-A2 : PDY-EPMGRVYQEYCEKMKELSLRIMELLELGLGV---EKRG--YYRDFFADSSSIMRCNYYPPCPEPERTLGTGPHCDP : 258
TaGA20ox-B2 : PDY-EPMGRVYQEYCGKMKELSLTIMELLELSLGV---EKRG--YYREFFADSSSIMRCNYYPPCPEPERTLGTGPHCDP : 258
TaGA20ox-D2 : PDY-EPMGRVYQEYCGKMKELSLRIMELLELSQGV---EKRG--YYREFFADSSSIMRCNYYPPCPEPERTLGTGPHCDP : 258
TaGA20ox-A3 : EEY-RDMGEVWQEYCDEMTRLALDV-DVLAACLGL---G-RG--ALRGFFAGDASLMRLNHYPPCQQPHLTLGTGPHHDP : 227
TaGA20ox-B3 : EEY-RDMGEVWQEYCDEMTRLALDVTDVLAACLGL---R-HG--ALRGFFAGDDSLMRLNHYPPCQQPHLTLGTGPHHDP : 230
TaGA20ox-D3 : EEF-RYMGEVWQEYCDEMTRLALDVTDVLAACLGL---G-RG--SLRGFFAGDDSLMRLNRYPPCQQPHLTLGTGPHHDP : 229
TaGA20ox-A4 : DDF-KHLGEVYQEYCEAMEEASLAIMEVLGVSLGL---G-RG--YYRDYFADGSSTMRCNNYPRCPEPDRTLGTGPHCDP : 251
TaGA20ox-B4 : GDF-KHLGEVYQEYCEAMEDASLAIMEVLGVSLGL---G-RG--YYRDFFADGSSIMRCNYYPRCPEPDRTLGTGPHCDP : 251
TaGA20ox-D4 : DDF-KHLGEVYQEYCEAMEEASLAIMEVLGMSLGL---G-RG--YYRDYFADGSSIMRCNYYPRCPEPDRTLGTGPHCDP : 251
OsGA3ox2    : DDY-LLFCDVMEEFHKEMRRLADELLRLFLRALGLTGEEVAGVEAERRIGERMTATVHLNWYPRCPEPRRALGLIAHTDS : 230
```

```
                          *        340         *        360         *        380         *        400
AtGA20ox1   : TSLTILHQD-HVNGLQVFVE------N--QWRSIRPNPKAFVVNIGDTFMALSNDRYKSCLHRAVVNSESERKSLAFFLC : 321
AtGA20ox2   : SSLTILHQD-HVNGLQVFVD------N--QWQSIRPNPKAFVVNIGDTFMALSNGIFKSCLHRAVVNRESARKSMAFFLC : 319
AtGA20ox3   : TSLTILHQD-QVGGLQVFVD------N--KWQSIPPNPHAFVVNIGDTFMALTNGRYKSCLHRAVVNSERERKTFAFFLC : 320
AtGA20ox4   : TSLTILQQD-QVSGLQVFVD------N--QWQSIPPIPQALVVNIGDTLMALTNGIYKSCLHRAVVNGETTRKTLAFFLC : 321
AtGA20ox5   : TAITVLLQD-DVVGLEVFAA------G--SWQTVRPRPGALVVNVGDTFMALSNGNYRSCYHRAVVNKEKVRRSLVFFSC : 323
BdGA20ox1   : TSLTILHQD-AVGGLQVHVD------G--RWRAIAPRQDAFVVNIGDTFMALSNGRYKSCLHRAVVNSKVPRKSLAFFLC : 304
BdGA20ox2   : TALTILLQD-DVGGLEVLVD------G--DWRPVRPVPGAMVINIGDTFMALSNGRYKSCLHRAVVNRQQERRSLAFFLC : 329
BdGA20ox3   : TSLTLLHQD-LVGGLQVFVG------G--EWRAVRPREDAFVVNIGDTFAALVDGRHASCLHRAVVNGAAARRSLTFFLN : 296
BdGA20ox4   : SALTLLMQDGGVDGLQVLVD------G--GWRPVRPKPDELVVNIGDTFMALSNGRYKSCLHRAVVHRERERRSLAYFLC : 339
HvGA20ox1   : TSLTILHQD-DVGGLQVHTD------G--RWRSIRPRADAFVVNIGDTFMALSNGRYKSCLHRAVVNSRVPRKSLAFFLC : 298
HvGA20ox2   : TALTILLQD-DVGGLEVLVD------G--DWRPVRPVPGAMVINIGDTFMALSNGRYKSCLHRAVVNRRQERRSLAFFLC : 358
HvGA20ox3   : TALTLLHQD-DVGGLEVFTG------G--AWRAVRPRSDAFVVNIGDTFSALTNGRHVSCLHRAVVNGSLARRALTFFLN : 296
HvGA20ox4   : SALTILLQDGEVDGLQVLVD------G--AWRSVRPKPGELVVNIGDTFMALSNGRYKSCLHRAVVHREKERRSLAYFLA : 344
OsGA20ox1   : TSLTILHQD-HVGGLEVWAE------G--RWRAIRPRPGALVVNVGDTFMALSNARYRSCLHRAVVNSTAPRRSLAFFLC : 308
OsGa20ox2   : TALTILLQD-DVGGLEVLVD------G--EWRPVSPVPGAMVINIGDTFMALSNGRYKSCLHRAVVNQRRERRSLAFFLC : 323
OsGA20ox3   : TSLTLLHQD-DVGGLQVLPDDAAAAAG--GWRAVRPRADAFVVNIGDTFAALTNGRHASCLHRAVVNGRVARRSLTFFLN : 302
OsGA20ox4   : SALTVLLQDGDVDGLQVLVA------G--AWRPVRPLPGAFVVNIGDTFMALTNGRYKSCLHRAVVHREQERRSLAFFLC : 370
TaGA20ox-A1 : TSLTILHQD-NVGGLQVHTE------G--RWRSIRPRADAFVVNIGDTFMALSNGRYKSCLHRAVVNSRVPRKSLAFFLC : 298
TaGA20ox-B1 : TSLTILHQD-NVGGLQVHTE------G--RWRSIRPRADAFVVNIGDTFMALSNGRYKSCLHRAVVNSKVPRKSLAFFLC : 298
TaGA20ox-D1 : TSLTILHQD-NVGGLQVHTE------G--RWRSIRPRADAFVVNIGDTFMALSNGRYKSCLHRAVVNSRVPRKSLAFFLC : 298
TaGA20ox-A2 : TALTILLQD-DVGGLEVLVD------G--DWRPVRPVPGAMVINIGDTFMALSNGRYKSCLHRAVVNRRQERRSLAFFLC : 329
TaGA20ox-B2 : TALTILLQD-DVGGLEVLVD------G--DWRPVRPVPGAMVINIGDTFMALSNGRYKSCLHRAVVNRRQERRSLAFFLC : 329
TaGA20ox-D2 : TALTILLQD-DVGGLEVLVD------G--DWRPVRPVPGAMVINIGDTFMALSNGRYKSCLHRAVVNRRQERRSLAFFLC : 329
TaGA20ox-A3 : TSLTLLHQD-DVGGLEVFTG------G--AWRAVRPRGDAFVVNIGDTFSALTNGRHISCLHRAVVNSSLARRSLTFFLN : 298
TaGA20ox-B3 : TSLTLLHQD-DVGGLEVFTG------G--AWRAVRPRSDAFVVNIDDTFSALTNGRHISCLHRAVVNSSLARRSLTFFLN : 301
TaGA20ox-D3 : ASLTLLHQD-DVGGLEVFTG------G--AWRAVRPRSDAFVVNIGDTFSALTNGRHISCLHRAVVNSSLARRSLTFFLN : 300
TaGA20ox-A4 : SALTILLQDGDVDGLQVLVD------G--AWRFVRPKTGELVVNIGDTFMALSNGRYKSCLHRAVVHREKERRSLAYFLS : 323
TaGA20ox-B4 : SALTILLQDGEVDGLQVLVD------G--AWRFVRPKTGELVVNIGDTFMALSNGRYKSCLHRAVVHREKERRSLAYFLS : 323
TaGA20ox-D4 : SALTILLQDGDVDGLQVLVD------G--AWRFVRPKTGELVVNIGDTFMALSNGRYKSCLHRAVVHREKERRSLAYFLA : 323
OsGA3ox2    : GFFTFVLQS-LVPGLQLFRR------GPDRWVAVPAVAGAFVVNVGDLFHILTNGRFHSVYHRAVVNRDRDRVSLGYFLG : 303

                          *        420         *        440         *        460         *        480
AtGA20ox1   : PKKDRVVTPPRELL----DSITSRRYPDFTWSMFLEFTQKHYRADMNTLQAFSDWLTKPI-------------------- : 377
AtGA20ox2   : PKKDKVVKPPSDIL----EKMKTRKYPDFTWSMFLEFTQKHYRADVNTLDSFSNWVITNNNPI----------------- : 378
AtGA20ox3   : PKGEKVVKPPEELVNG--VKSGERKYPDFTWSMFLEFTQKHYRADMNTLDEFSIWLKNRRSF------------------ : 380
AtGA20ox4   : PKVDKVVKPPSELE-------GERAYPDFTWSMFLEFTMKHYRADMNTLEEFTNWLKNKGSF------------------ : 376
AtGA20ox5   : PREDKIIVPPPELVE---GEEASRKYPDFTWAQLQKFTQSGYRVDNTTLHNFSSWLVSNS-------------------- : 380
BdGA20ox1   : PEMDKTVAPPGKLV----DEENPRVYPDFTWRALLDFTQKHYRADMKTLEVFSGWVLQQQ-------------------K : 361
BdGA20ox2   : PRQDRVVRPPLAVV----SSSAPRQYPDFTWADLMRFTQRHYRADTRTLDAFTQWLAPSSSSSSSSSPAPSAAPPPPVAS : 405
BdGA20ox3   : PPLDRVVSPPQGLLVAKDGGAINRKYPGFTWREFLEFTQKHYRSDTNTMEAFVDWIKQGRRGAQFHGD-----------A : 365
BdGA20ox4   : PRKDRVVRPPPST-----TAPAPRLYPDFTWADLSRFTQRHYRADARTLDAFARWLGPAK-------------------- : 394
HvGA20ox1   : PEMDKVVAPPGTLV----DAANPRAYPDFTWRSLLDFTQKHYRADMKTLEVFSSWVVQQQ-------------------- : 354
HvGA20ox2   : PREDRVVRPPPSLR-------SPRQYPDFTWADLMRFTQRHYRADTRTLDAFTQWF-----------------------S : 408
HvGA20ox3   : PQLDRPVTPPAELLA---IDGRPRVYPDFTWREFLEFTQKHYRSDSRTLDAFVAWINQGHTG------------------ : 355
HvGA20ox4   : PREDRVVRPPPS------PAPAPRLYPDFTWAELMRFTQRHYRADARTLDAFACWLDLPSCPTTP--------------- : 403
OsGA20ox1   : PEMDTVVRPPEELV----DDHHPRVYPDFTWRALLDFTQRHYRADMRTLQAFSDWLNHHRH------------------L : 366
OsGa20ox2   : PREDRVVRPPP-------SAATPQHYPDFTWADLMRFTQRHYRADTRTLDAFTRWLAPPAADAAAT-------------A : 383
OsGA20ox3   : PRLDRVVSPPPALV----DAAHPRAFPDFTWREFLEFTQRHYRSDTNTMDAFVAWIKQRNG------------------Y : 360
OsGA20ox4   : PREDRVVRPPAGA-----GAGERRLYPDFTWADFMRFTQRHYRADTRTLDAFARWLRPPAC----SGAAPVVG--PPTTA : 439
TaGA20ox-A1 : PEMDKVVAPPGTLV----DASNPRAYPDFTWRSLLDFTQKHYRADMKTLEVFSSWIVQQQQGQL---------------A : 359
TaGA20ox-B1 : PEMDKVVAPPGTLV----DAANPRAYPDFTWRSLLDFTQKHYRADMKTLEVFSSWIVQQQQGQLAL-------------Q : 361
TaGA20ox-D1 : PEMDKVVAPPGTLV----DAANPRAYPDFTWRSLLDFTQKHYRADMKTLEVFSSWIVQQQ-------------------Q : 355
TaGA20ox-A2 : PREDRVVRPPPGLR-------SPRRYPDFTWADLMRFTQRHYRADTRTLDAFTQWFSSSTSPPPPA-------------P : 389
TaGA20ox-B2 : PREARVVRPPPGLR-------SPRQYPDFTWADLMRFTQRHYRADTRTLDAFTQWFSSSSS------------------S : 384
TaGA20ox-D2 : PREDRVVRPPPGLR-------SPRRYPDFTWADLMRFTQRHYRADTRTLDAFTQWFSSSS-------------------S : 383
TaGA20ox-A3 : PQLDCPVAPPAELLA---I-GRPRAYPDFTWREFLEFTQKHYRSDWRTLDAFAAWINQGRKG------------------ : 356
TaGA20ox-B3 : PQLDRLVAPPAELLA---VDGRPRVYPDFTWREFLEFTQKHYRSDWRTLDAFASWINQGRKG------------------ : 360
TaGA20ox-D3 : PQLDRPVAPPTELLA---IDGRPRAYPGFTWREFLEFTQKHYRSDWRTLDAFAAWINQGRKG------------------ : 359
TaGA20ox-A4 : PREDRVVRPPPS------PAPAPRLYPDFTWAELMRFTQRHYRADSRTLDAFARWLDPPSFSAAPLPHGP---------A : 388
TaGA20ox-B4 : PREDRVVRPPPS------PAPAPRLYPDFTWAELMRFTQRHYRADSRTLDAFARWLDPPSFSAAPLPHGP---------A : 388
TaGA20ox-D4 : PREDRVVRPPPS------PAPAPRLYPDFTWAELMRFTQRHYRADARTLDAFARWLDPPTCSATPLSHGP---------A : 388
OsGA3ox2    : PPPDAEVAPLPEAV----PAGRSPAYRAVTWPEYMAVRKKAFATGGSALKMVSTDAAAAADEHDDVAA-----------A : 368
```

```
AtGA20ox1   : ------ :   -
AtGA20ox2   : ------ :   -
AtGA20ox3   : ------ :   -
AtGA20ox4   : ------ :   -
AtGA20ox5   : DKKST- : 385
BdGA20ox1   : QKQLGS : 367
BdGA20ox2   : TAQEAA : 411
BdGA20ox3   : GQAEKN : 371
BdGA20ox4   : -AQEIV : 399
HvGA20ox1   : PASART : 360
HvGA20ox2   : SAQEAA : 414
HvGA20ox3   : ------ :   -
HvGA20ox4   : QAQGTV : 409
OsGA20ox1   : QPTIYS : 372
OsGa20ox2   : QVEAAS : 389
OsGA20ox3   : ESLDKY : 366
OsGA20ox4   : TQAATV : 445
TaGA20ox-A1 : LQPAMT : 365
TaGA20ox-B1 : PQPART : 367
TaGA20ox-D1 : PQPART : 361
TaGA20ox-A2 : AAQQAA : 395
TaGA20ox-B2 : SAQEAA : 390
TaGA20ox-D2 : SAQEAA : 389
TaGA20ox-A3 : ------ :   -
TaGA20ox-B3 : ------ :   -
TaGA20ox-D3 : ------ :   -
TaGA20ox-A4 : QAQGTV : 394
TaGA20ox-B4 : QAQGTV : 394
TaGA20ox-D4 : QAQGTV : 394
OsGA3ox2    : ADVHA- : 373
```

Figure S9. Alignment of GA3ox-related protein sequences using MUSCLE with OsGA2ox1 as outgroup.

```
                      *        20         *        40         *        60         *        80
AtGA3ox1    : ---------MPAMLTDVFRGHPIHLPHSHIPDFTSLRELPDSYKW--TPKDDLLFSAAPSPPATGEN----IPLIDLDH- :  64
AtGA3ox2    : ---------MSSTLSDVFRSHPIHIPLSNPPDF---KSLPDSYTW--TPKDDLLF-----SASASDET---LPLIDLSD- :  57
AtGA3ox3    : ----------MSSVTQLFKNNPVNRDRIIPLDFTNTKTLPDSHVW------SKPEPETTSGP---------IPVISLSN- :  54
AtGA3ox4    : ---------MPSLAEEICIG-----------NLGSLQTLPESFTWKLTAADSLLRPSSAVSFDAVEES---IPVIDLSN- :  56
BdGA3ox2a   : ----------MPTASHL-TS-----PR--YFDFRAARGVPESHAW--PGLHDHPVVDGGAGAGAGEDDADRVPVVDMRAD :  60
BdGA3ox2b   : ----------MPTATHL--------------DFQAARGVPETHEW--PVLHDHPVVDG--GAGAGEDE---VPVVDLRDA :  49
HvGA3,18ox1: ------------MPSQLNKD-----PHNRYFDLGAAREVPDTHAW--DGQHELPVVDG--GVGAGDDA---VPVVDMRD- :  55
HvGA3ox2    : ----------MPTPSHLSKD-----PH--YFDFRAARRVPETHAW--PGLHDHPVVDGG-GAGGGPDA---VPVVDMRD- :  56
OsGA3ox1    : MQIMTSSSTSPTSPTSPLAAAADNGVAAAYFNFRGAERVPESHVW--KGMHEKDTAPVAAADADGGDA---VPVVDMSGG :  75
OsGA3ox2    : ----------MPTPSHL-KN-----PL--CFDFRAARRVPETHAW--PGLDDHPVVDGG-G-GGGEDA---VPVVDVGA- :  54
TaGA1ox-B1 : ----------MPTPSKLNKD-----PHNHYFDLGAARQVPETHAW--EGLHEHPVVDG--GVGAGEDA---VPVVDMQD- :  57
TaGA3ox-A2 : ----------MPTPSHLSKD-----PR--YFDFRAARRVPETHAW--PGLHDHPVVDGS-GAGGGPDA---VPVVDMRD- :  56
TaGA3ox-A3 : ----------MSAPSQLSKD-----PHNDYFDFGAARQVPETHTW--EGLYEHPLVDG--GVGAAEDA---VPVVDLRD- :  57
TaGA3ox-B2 : ----------MPTPAHLSKD-----PH--YFDFRAARRVPETHAW--PGLHDHPVVDGS-GAGGEPDA---VPVVDMRD- :  56
TaGA3ox-B3 : ----------MSAPSQLSKD-----PRNNYFDFGAARQVPETHMW--EGLYEHPVVDG--GVRAGEDA---VPVVDMQD- :  57
TaGA3ox-D2 : ----------MPTPAHLSKD-----PR--YFDFRAARRVPETHAW--PGLHDHPVVDGS-GAGGGPDA---VPVVDMRD- :  56
TaGA3ox-D3 : ----------MSAPSQLSKH-----PHNNYFDFGAARQVPETHTW--EGLYEHPLVDG--GVGAAEDA---VPVVDLRD- :  57
OsGA2ox1    : --------MVVPSATTPARQ-----------ETVVAAAPPAAAAS-----------GVVGGGGGVT----IATVDMSAE :  45

                      *       100         *       120         *       140         *       160
AtGA3ox1    : -P-DATNQIGHACRTWGAFQISNHGVPLG--LLQDIEFLTGSLFGLPVQRKLKSARSE-TGVSGYGVARIASFFNKQMWS : 139
AtGA3ox2    : -I-HVATLVGHACTTWGAFQITNHGVPSR--LLDDIEFLTGSLFRLPVQRKLKAARSE-NGVSGYGVARIASFFNKKMWS : 132
AtGA3ox3    : -P-EEHGLLRQACEEWGVFHITDHGVSHS--LLHNVDCQMKRLFSLPMHRKILAVRSP-DESTGYGVVRISMFYDKLMWS : 129
AtGA3ox4    : -P-DVTTLIGDASKTWGAFQIANHGISQK--LLDDIESLSKTLFDMPSERKLEAASSD-KGVSGYGEPRISPFFEKKMWS : 131
BdGA3ox2a   : PS-AAAAAVARAAETWGAFLLEGHGVPES--LLARVEERVAGMFALPAPEKMRAVRGR-GESCGYGSPPISSFFAKSMWS : 136
BdGA3ox2b   : DPAKVAAAMARAAEQWGAFLLLGHGVPAD--LVARVEERIEAMFALPASEKTRAVRGR-GESCGYGSPPISGFFDKSMWS : 126
HvGA3,18ox1: -P-RAAEAVARASEQWGAFLLEGHGVPTE--LLARVEAGIAGMFALPTPEKMRAARHD-GDLYGYGPPLIASYVSKNMWS : 130
HvGA3ox2    : -P-CAAEAVALAAQDWGAFLLQGHGVPLE--LLARVEAAIAGMFALPASEKMRAVRRP-GDSCGYGSPPISSFFSKCMWS : 131
OsGA3ox1    : DD-AAVAAVARAAEEWGGFLLVGHGVTAE--ALARVEAQAARLFALPADDKARGARRPGGGNTGYGVPPYLLRYPKQMWA : 152
OsGA3ox2    : -G-DAAARVARAAEQWGAFLLVGHGVPAA--LLSRVEERVARVFSLPASEKMRAVRGP-GEPCGYGSPPISSFFSKLMWS : 129
TaGA1ox-B1 : -P-HAAEAVARASEQWGAFLLQGHGVPRE--LLARVEAGIAGMFALPKTDKMRAARQG-GDPYGYGLPHIALYFSKTMWS : 132
TaGA3ox-A2 : -P-CAAEAVALAAQDWGAFLLEGHGVPLE--LLAGVEAAIGGMFALPASEKMRAVRRP-GDSCGYGSPPISSFFSKCMWS : 131
TaGA3ox-A3 : -P-HAAEAVARASEQWGTFLLEGHGIPSE--LLARVEARIASVFALPASEKMRAARQD-GQSHGYGLPPIASYFPKTTWS : 132
TaGA3ox-B2 : -P-FAAEAVGLAAQDWGAFLLVGHGVPLD--LLVRVEAAIAGMFALPASEKMRAVRRP-GDSCGYGSPPISSFFSKCMWS : 131
TaGA3ox-B3 : -P-RAAEAVARASEQWGVFLLEGHGIPSE--LLARVEAGIAGMFALPTPEKMRAERQD-GEPYGYGLPHIASYSSKATWS : 132
TaGA3ox-D2 : -P-CAAEAVALAAQDWGAFLLEGHGVPLE--LLARVEAAIAGMFALPASEKMRAVRRP-GDSCGYGSPPISSFFSKCMWS : 131
TaGA3ox-D3 : -P-HAAEAVARASEQWGTFLLEGHGIPSE--LLARVEAGIASMFALPASEKMRAARQD-GQSHGYGLPPIASYFPKTTRS : 132
OsGA2ox1    : RG-AVARQVATACAAHGFFRCVGHGVPAAAPVAARLDAATAAFFAMAPAEKQRA--GP-ASPLGYGCRSIG--FNGDVGE : 119

                      *       180         *       200         *       220         *       240
AtGA3ox1    : EGFTITGSPLN-DFRKLWPQ---HHLNYCDIVEEYEEHMKKLASKLMWLALNSLGVSEEDIEWASLSSDLNWAQA---AL : 212
AtGA3ox2    : EGFTVIGSPLH-DFRKLWPS---HHLKYCEIIEEYEEHMQKLAAKLMWFALGSLGVEEKDIQWAGPNSDFQGTQA---VI : 205
AtGA3ox3    : EGFSVMGSSLRRHATLLWPD---DHAEFCNVMEEYQKAMDDLSHRLISMLMGSLGLTHEDLGWLVPDKTGSGTDSIQSFL : 206
AtGA3ox4    : EGFTIADDSYRNHFNTLWPH---DHTKYCGIIQEYVDEMEKLASRLLYCILGSLGVTVEDIEWAHKLEKSGSKVGRG-AI : 207
BdGA3ox2a   : EGYTFSPAHLRRDLRKLWPKAGPDYLLFCEVMEEFHREMRLLADKLLGLFLRALGLTADEALAVEATHRIAETMTA--TT : 214
BdGA3ox2b   : EGYTFAPANVRLDLLKLWPDAGPHYLLFCEVMEEFHKGMFALAHKLLGLFLSALGLTAADCAAAA---KIADTMAA--SM : 201
HvGA3,18ox1: EGYTLTPANLHAEFRKIWPDAGQHYRHFSGVMEEFLKEMRSLANRLMELFLVALGLTAEQIAGVEAEHNLTESMTE--TM : 208
HvGA3ox2    : EGYTFSPANLRSDLRKLWPKAGHDYRHFCAVMEEFHREMRVLADKLLELFLVALGLTGEQVAAVESEHKIAETMTA--TM : 209
OsGA3ox1    : EGYTFPPPAIRDEFRRVWPDAGDDYHRFCSAMEEYDSSMRALGERLLAMFFKALGLAGNDAPGGETERKIRETLTS--TI : 230
OsGA3ox2    : EGYTFSPSSLRSELRRLWPKSGDDYLLFCDVMEEFHKEMRRLADELLRLFLRALGLTGEEVAGVEAERRIGERMTA--TV : 207
TaGA1ox-B1 : EGYSLSPTNLRPELRKIWPDAGHDYSQFCGVMEEFHKEMRALADKLMELFLVALGLTGPQIAGVEAEHKLTETMSE--TI : 210
TaGA3ox-A2 : EGYTFSPANLRSDLRKLWPKAGHDYRHFCAVMEEFHREMRALADKLLELFLVALGLTGEQVAAVESEHKIAETMTA--TM : 209
TaGA3ox-A3 : EGYTLSPANLRAELRKIWPDAGGDYRHFCDVMEEFHRQMLAVVDKLTVLFLAALGVAS-----VEAGRKIVETMSETVTM : 207
TaGA3ox-B2 : EGYTFSPANLRSDLRKLWPKAGHDYRHFCAVMEEFHREMRALADKLLELFLVALGLTGEQVAAVESEQKIAETMTA--TM : 209
TaGA3ox-B3 : EGYTLSPASLRAELRRVWPDAGEDYRHFCDVMEEFHRQMRAVADKLTVLFLAALGLAGEQIAAVEAERKIVETMSETVTM : 212
TaGA3ox-D2 : EGYTFSPANLRSDLRKLWPKAGHDYRHFCAVMEEFHREMRALADKLLELFLVALGLTGEQVAAVESEQKIAETMTA--TM : 209
TaGA3ox-D3 : EGYTLSPANLRAELRKMWPDAGEDYRHFCDVMEEFHRQMRAVADKLTVLFLAALGVAG-----VEAERKIVDTMSETVTM : 207
OsGA2ox1    : LEYLLLHANPAAVAHRARTIDAMDPSRFSAIVNEYIEAMKKLACEILDLLGEGLGLKDPRYFS-----KLTTNADSDCLL : 194
```

```
                        *        260         *        280         *        300         *        320
AtGA3ox1    : QLNHYPVCPEPDR---------------------------AMGLAAHTDSTLLTILYQNNTAGLQV---FRDDL---GWV : 259
AtGA3ox2    : QLNHYPKCPEPDR---------------------------AMGLAAHTDSTLMTILYQNNTAGLQV---FRDDV---GWV : 252
AtGA3ox3    : QLNSYPVCPDPHL---------------------------AMGLAPHTDSSLLTILYQGNIPGLEIESPQEEGS---RWI : 256
AtGA3ox4    : RLNHYPVCPEPER---------------------------AMGLAAHTDSTILTILHQSNTGGLQV---FREES---GWV : 254
BdGA3ox2a   : HLNWYPRCPDPKR---------------------------ALGLIPHTDSGYFTFVLQSQVPGLQL---FRRGP--DRWV : 262
BdGA3ox2b   : HLNWYPKCPDPKR---------------------------AVGLIAHTDSGYFTFVLQSMVPGLQL---FRREP--DRWV : 249
HvGA3,18ox1: HLNWYPKCPDPTR---------------------------ALGLKGHTDSGFFTFVMQSQVPGLHL---FRHGPPADRWV : 258
HvGA3ox2    : HLNWYPKCPDPKR---------------------------ALGLIAHTDSGFFTFVLQSLVPGLQL---FRHGP--DRWV : 257
OsGA3ox1    : HLNMFPRCPDPDR---------------------------VVGLAAHTDSGFFTFILQSPVPGLQL---LRHRP--DRWV : 278
OsGA3ox2    : HLNWYPRCPEPRR---------------------------ALGLIAHTDSGFFTFVLQSLVPGLQL---FRRGP--DRWV : 255
TaGA1ox-B1 : HLNWYPKCPDPKR---------------------------ALGMKAHTDSGFFALLMQSQVPGLHL---FRHGPPADRWV : 260
TaGA3ox-A2 : HLNWYPKCPDPKR---------------------------ALGLIAHTDSGFFTFVLQSLVPGLQL---FRHGP--DRWV : 257
TaGA3ox-A3 : HINWYPKCPDPKR---------------------------TLGLAIHTDSGFFTFLMQSHVSGLQL---FRHEP--DRWV : 255
TaGA3ox-B2 : HLNWYPKCPDPKR---------------------------ALGLIAHTDSGFFTFVLQSLVPGLQL---FRHGP--DRWV : 257
TaGA3ox-B3 : HLNWYPKCPDPKR---------------------------ALGMTTHTDSGFFTFVMQSHVPGMQL---FRRGP--DRWV : 260
TaGA3ox-D2 : HLNWYPKCPDPKR---------------------------ALGLIAHTDSGFFTFVLQSLVPGLQL---FRHGP--DRWV : 257
TaGA3ox-D3 : HLNWYPKCPDPKR---------------------------TLGLATHTDSGFFTFVMQSHVSGMQL---FRRGP--DRWV : 255
OsGA2ox1    : RINHYPPSCNIHKLDHDDQCNIKSLVSTKASNGGNLMAGGRIGFGEHSDPQILSLLRANDVEGLQVFVPDHEGK--EMWV : 272

                        *        340         *        360         *        380         *        400
AtGA3ox1    : TVPPFPGSLVVNVGDLFHILSNGLFKSVLHRARVNQTRARLSVAFLW-GPQSDIKISPVPKLVSPVES-PLYQSVTWKEY : 337
AtGA3ox2    : TAPPVPGSLVVNVGDLLHILTNGIFPSVLHRARVNHVRSRFSMAYLW-GPPSDIMISPLPKLVDPLQS-PLYPSLTWKQY : 330
AtGA3ox3    : GVEPIEGSLVVIMGDLSHIISNGQFRSTMHRAVVNKTHHRVSAAYFA-GPPKNLQIGPLTS--DKNHP-PIYRRLIWEEY : 332
AtGA3ox4    : TVEPAPGVLVVNIGDLFHILSNGKIPSVVHRAKVNHTRSRISIAYLWGGPAGDVQIAPISKLTGPAEP-SLYRSITWKEY : 333
BdGA3ox2a   : AVPAVPGAFVVNVGDLFSILTNGRFHSVFHRAVVNKESHRVSLGYFL-GPPAQTRVGPLEEALTPARPKPMYRPVTWPEY : 341
BdGA3ox2b   : AVPAMPGAFVINVGDLFNIVTNGRFHNVFHRAVVSRESHRVSLGYFL-GPPAQAVVAPLDEALTLDRPRPAYRPVVWREY : 328
HvGA3,18ox1: EVPAVPGALIVNIGDLFQILTNGRFRSVYHRVVVNRERERISVAYFL-IPPADVKVAPLKEVVGGGK--PVYRALTWSES : 335
HvGA3ox2    : TVPAVPGAMVVNVGDLFHILTNGRFHSVYHRAVVNRDSDRISLGYFL-GPPAHVKVAPLREAL-AGTP-AAYRAVTWPEY : 334
OsGA3ox1    : TVPGTPGALIVVVGDLFHVLTNGRFHSVFHRAVVNRERDRISMPYFL-GPPADMKVTPLVAAGSPESK-AVYQAVTWPEY : 356
OsGA3ox2    : AVPAVAGAFVVNVGDLFHILTNGRFHSVYHRAVVNRDRDRVSLGYFL-GPPPDAEVAPLPEAVPAGRS-PAYRAVTWPEY : 333
TaGA1ox-B1 : EVPAVPGALVVNIGDLFQILTNGRFRSVYHRAVVNRDRERISLAYFL-GPAANAKVGPLKEVVGGGR--PAYRALTWPEY : 337
TaGA3ox-A2 : TVPAVPGAMVVNVGDLFQILTNGRFHSVYHRAVVNRDSDRISLGYFL-GPPAHVKVAPLREAL-AGTP-AAYRAVTWPEY : 334
TaGA3ox-A3 : GVP--PGALIVNIGDLFQILTNGRFRSTYHRAVVNRESTRVSVAYHL-GPPADAKVAPLREAVGGGK--PAYRTVTWREY : 330
TaGA3ox-B2 : TVPAVPGAMVVNVGDLFQILTNGRFHSVYHRAVVNRESDRISLGYFL-GPPAHVKVAPLREAL-AGTP-AAYRAVTWPEY : 334
TaGA3ox-B3 : GVP--PGALIVNIGDLFQILTNGRFRSAYHRAVVNRDNARVSVAYHL-GPPADVKVAPLREAVGAGK--PAYRTVTWREY : 335
TaGA3ox-D2 : TVPAVPGAMVVNVGDLFQILTNGRFHSVYHRAVVNRDSDRISLGYFL-GPPAHVKVAPLREAL-AGTP-AAYRAVTWPEY : 334
TaGA3ox-D3 : --PGH??ALIVNISDLFQILTNGRFCSTYHRAVVNRDSTRVSVAYHL-GPPAGVKVAPLREAVGCGK--PAYRTVTWREY : 328
OsGA2ox1    : QVPSDPSAIFVNVGDVLQALTNGRLISIRHRVIATACRPRLSTIYFA-SPPLHARISALPETITASSP-RRYRSFTWAEY : 350

                        *        420         *        440         *
AtGA3ox1    : LRTKATHFNK------------ALSMIR-----NHREE---------------- : 358
AtGA3ox2    : LATKATHFNQ------------SLSIIR-------------------------- : 346
AtGA3ox3    : LAAKATHFNK------------ALTLFRC------------------------- : 349
AtGA3ox4    : LQIKYEVFDK------------AMDAIRVVNPTN-------------------- : 355
BdGA3ox2a   : MTVRKKAFAT---------GDSALQMVAVDQD-DDDDAQTKLANRKIKVSGEAY : 385
BdGA3ox2b   : MGLREKALFV---------GESALKMIAVAKD-DAPQTPKLHETS--------- : 363
HvGA3,18ox1: IVVRKEAFANHGADLEFGKGRTALDMLSISSDEDDGAEHHRD------------ : 377
HvGA3ox2    : MGVRKKAFTT---------GASALKMVAISTD-DAADVLPDVLSS--------- : 369
OsGA3ox1    : MAVRDKLFGT---------NISALSMIRVAKEEDKES----------------- : 384
OsGA3ox2    : MAVRKKAFAT---------GGSALKMVSTDAA-AAADEHDDVAAAADVHA---- : 373
TaGA1ox-B1 : IIVRKEAFANGGADLEFTKGGTALEMVSINPDDDDGMDDQGDISS--------- : 382
TaGA3ox-A2 : MGVRKKAFTT---------GASALKMVAISTDNDAANDTDDLISS--------- : 370
TaGA3ox-A3 : FLVRKEAFTT---------GGSALQMVSLSPG-DHDDDGDGADQISEISS---- : 370
TaGA3ox-B2 : MGVRKKAFTT---------GASALKMVAISTD-DAANDTDDLILS--------- : 369
TaGA3ox-B3 : IVVRKEAFAT---------GGSALEMVSLSPD-DHDDEGDGADHISEIPS---- : 375
TaGA3ox-D2 : MGVRKKAFTT---------GASALKMVAISTDNDAANHTDDLISS--------- : 370
TaGA3ox-D3 : FLVRKEAFAT---------GGSALQMVSLSPG-DHKNHGDGADQISEISS---- : 368
OsGA2ox1    : ---KTTMYSLR-------LSHSRLELFKIDDD-DSDNASEGKA----------- : 382
```

Figure S10. Alignment of GA2ox protein sequences using MUSCLE with OsGA3ox2 as outgroup.

```
                               *        20         *        40         *        60         *        80
AtGA2ox1     : MAV-LSK---------PVAIPK----------------------------SGF----------SLIPVIDMSDP--- :  26
AtGA2ox2     : MVV-LPQ---------PVTLDN--HISLIP---------------TYKPVPVL---------TSHSIPVVNLADP--- :  39
AtGA2ox3     : MVI-VLQ---------PASFDS----NLYV---------------NPKCKPRP----------VLIPVIDLTDS--- :  35
AtGA2ox4     : MVKGSQK---------IVAVDQ----------------------------------------------DIPIIDMSQE--- :  23
AtGA2ox6     : MVL-PSS---------TPLQTT-----------------------GKKTISSP---------EYNFPVIDFSLN--- :  32
AtGA2ox7     : MAS------------QPPFKTN--FCSIFG-------------SSFPNSTSESNTNTSTIQTSGIKLPVIDLSHL--- :  48
AtGA2ox8     : MDPPFNE-----------IYNNLLYNQITK--------------KDNDVSEIPFSFSVTAVVEEVELPVIDVSRL--- :  50
BdGA2ox1     : MVVPSST---------TPARQE-----------------------IATAAAL---------QLPAIPTVDMSAP--- :  34
BdGA2ox10    : MVV-LAQ----------GELEQ---IALPA---------------VQKAAPPL----------ADVPEIDLAAA--- :  35
BdGA2ox2     : MVM-------------PASTAS-----------------------SAACRDTA---------SGAGIPTVDMSAP--- :  30
BdGA2ox3     : MVV-LAS---------PPAADH---IPLLR---------------SPDPGDYF----------SGMPVIDLCSP--- :  36
BdGA2ox4     : MVV-LAK---------PAALEQ---ISLLR---------------TPEPWESF----------AGVPAVDLSAP--- :  36
BdGA2ox5     : MEDHEYEQ-----DESNPPLLAT--YKHLLD---------------GEHRRLPLGVVAGDEEDDQCDLPVIDLAPL--- :  54
BdGA2ox6     : MPALFAD-----GATSDPPLADS--YRALLR-----------SGGIAHPPEESL------SPVLERDLPMIDIECL--- :  52
BdGA2ox7     : MVV-LAK----------GELEQ---IALPA---------------AQSPP-------------ANVQAVDLSSA--- :  32
BdGA2ox8a    : MAAITMTA--------LPSSVDHQVVIPLLK---------------CPIAAAAA----------AAMIPTVDLSSP--- :  43
BdGA2ox8b    : MVA-IKA---------PSSIDH--ANPLTK---------------SPKAAAAA----------AAAIPTVDLSSP--- :  38
BdGA2ox9     : MPA-FAE------SAAEPPLADS--YHALLR---RAGADGRNKDAHCTPTVHADHVLPAPDMAVSECELPMIDVGCL--- :  65
HvGA2ox1     : MVV-PS---------TTPARQE-TATLLLP---------------QAQPSPGG----------GAIPTVDMSAP--- :  38
HvGA2ox10    : MVV-LAK----------GELEQ---IALPA---------------VHKTAPPL----------ADLPEVDLSVA--- :  35
HvGA2ox2     : MVASTAA---------PASSSA---AAV-----------------APACRGMA---------PAGIPTIDMSAP--- :  36
HvGA2ox3     : MVV-LAG---------TPAVDH---IPLLR---------------SPDPGDNF----------SGMPVVDLSRP--- :  36
HvGA2ox4     : MVV-LAK---------PAALEQ---IALMR---------------TPEPWESF----------SGVPAVDLSSP--- :  36
HvGA2ox6     : MPA-FAE------PAIDPPLADS--YRALLR--------------SDERAGIAAAPATGSVAVLERDLPMIDLERL--- :  53
HvGA2ox7     : MVV-LAK----------GELEQ---IALPA---------------AQP-------------PLAHVRAIDLSAA--- :  32
HvGA2ox8     : TVA-ITV---------PISVDT---IPLLK---------------CVNAATAA------------VPSVDLSAP--- :  34
HvGA2ox9     : MPA-FAE------STAEPPLSDS--YYALLR-----------RGNKVDECASVPPPGCQVLPVAECELPMIDVGCL--- :  56
OsGA2ox1     : MVVPSAT---------TPARQE---TVVAA---------------APPAAAAS---GVVGGGGGVTIATVDMSAE--- :  45
OsGA2ox10    : MVV-LAK----------GELEQ---IALPA---------------AVQKAAPP---------LADVPEVDLGGG--- :  36
OsGA2ox2     : MVVPAAA---------APECGR------------------------REAAAAAAAAVFCRRGRGVVVPTVDMSAP--- :  42
OsGA2ox3     : MVV-LAG---------PPAVDH---IPLLR---------------SPDPGDVF----------SGVPVVDLGSP--- :  36
OsGA2ox4     : MVV-LAK---------PAALEQ---ISLVR---------------SPSVEDNF---------GAGLPVVDLAAD--- :  37
OsGA2ox5     : MEEHDYD----SNS--NPPLMST--YKHLFV---------------EQHRLDMD---MGAIDVDECELPVIDLAGL--- :  50
OsGA2ox6     : MPA-FAD------IAIDPPLADSYRALALLR---------RDRDGGIAPPAVQMV---GSGGAVLERDLPMVDLERL--- :  58
OsGA2ox7     : MVV-LAK----------GELEQ---IALPA---------------AHPPP-------------ADVRAIDLSAT--- :  32
OsGA2ox8     : MVA-ITA---------PSSIEH---IPLVR---------------CPKGANAG---------PQAVIPCIDLSAP--- :  38
OsGA2ox9     : MPA-IAD------CAADPPLADS--YYTLLR-------------LGGDDDDDACTKVTTTPQPVSECELPMIDVGCLTAP :  58
TaGA2ox-A1   : MVVPSTT---------SARQET--ATMLLP---------------PPCPGGGA------------IPTVDMSAP--- :  36
TaGA2ox-A10  : MVV-LAK----------GELEQ---IALPA---------------VHKTAPPL----------ADVPEVDLSVA--- :  35
TaGA2ox-A11  : MLA-FAE--GTSCTTTDPPLADS--YRALLC-------------RGELEGVGAASAHAVESLAMLERDLPIIDLERL--- :  59
TaGA2ox-A3   : MVV-LAG---------SPAVDH---IPLLR---------------SPDPGDNF----------SGMPVVDLSSP--- :  36
TaGA2ox-A4   : MVV-LAK---------PAALEQ---IALLR---------------TPEAWESF----------SGVPAVDLSGP--- :  36
TaGA2ox-A6   : MPA-FAE----PAI--DPPLADS--YRALLRTDQLRRGGIAPALSPAVPESVAVL---------ERDLPMIDLKRL--- :  58
TaGA2ox-A7   : MVV-LAK----------GELEQ---IALPA---------------AQP-------------PLAHVRAVDLSAA--- :  32
TaGA2ox-A8   : MVA-ITV---------PISVEA---IPLVK---------------CAHAAAAA------------VPSVDLSAP--- :  34
TaGA2ox-A9   : MPA-FAE------STAEPPLADS--YYALLR-----------GGNKADECASAPPPGCQAPPVSECELPMIDVGCL--- :  56
TaGA2ox-B1   : MVVPSTT---------PARQET--VTTLLP---------------PPCPGGGA------------IPTVDMSAP--- :  36
TaGA2ox-B10  : MVV-LAK----------GELEQ---IALPA---------------VHKTAPPL----------ANMPEVDLAVA--- :  35
TaGA2ox-B11  : MLA-FAK--RTSCTTTDPPLADS--YRALLC-------------RGKLEGGGAASAHAVESLAMLERDLPIIDLKRL--- :  59
TaGA2ox-B12  : MPA-SGE----GGTTSDPPLVDS--YRTLLR--------------KGGGDGFVPALAAESLAVLECDLPMIDLERL--- :  55
TaGA2ox-B13  : MAA-FDE---NGTT--DPPLADS--YRKLLR---------------RDELGGAL--ARESLAVLERDLPMIDLKRL--- :  51
TaGA2ox-B2   : MVASSAA---------PASSSA-----------------------APACRGMA---------PAGIPTIDMSAP--- :  33
TaGA2ox-B3   : MVV-LAS---------TPAVDH---IPLLR---------------SPDPGDYF----------SGMPVVDLSSP--- :  36
TaGA2ox-B4   : MVV-LAK---------PAALEQ---ITLLR---------------TPEPWESF----------SGIPAVDLSGP--- :  36
TaGA2ox-B6   : MPA-FAE------PAIDPPLADS--YRALLR------SDQLHRGGIAPALSPAV---LESVAVLERDLPMIDLKRL--- :  58
TaGA2ox-B7   : MVV-LAK----------GELEQ---IALPA---------------AQPP-------------LAHVRAIDLSAA--- :  32
TaGA2ox-D1   : MVVPSTT---------PARQET--ATMLLP---------------PPSAGGGA------------IPTVDMSAP--- :  36
TaGA2ox-D10  : MVV-LAK----------GELEQ---IALPA---------------VHKTAPPL----------ADVPEVDLAVA--- :  35
TaGA2ox-D11  : MLA-FAE--GTSCTTTDPPLADS--YRALLC-------------RGELEGGGAAPAHALESLAMLERDLPIIDLKHL--- :  59
TaGA2ox-D2   : MVASSAA---------PASSSA----------------------AAPACRDMA---------PAGIPTIDMSAP--- :  34
TaGA2ox-D3   : MVV-LAS---------TPAVDH---IPLLR---------------SPDPGDYF----------SGMPVVDLSSP--- :  36
TaGA2ox-D4   : MVV-LAK---------PAALEQ---ITLLR---------------PPEPWESF----------SGVPAVDLSGP--- :  36
TaGA2ox-D6   : MPA-FAE------PAIDPPLADS--YRALLR-------------SDQLRRGGIAPAPASESVAVLERALPMIDLKRL--- :  55
TaGA2ox-D7   : MELTCKQYWRKAFD--CQKMTEVENDLDLLA----------------------------IVLKALTELSFLEVCTA--- :  46
TaGA2ox-D8   : MVG-ITV---------PMSMDK---IPLVK---------------CANAAAAA-----------AVPTVDLSAG--- :  35
TaGA2ox-D9   : MPA-FAE------STAEPPLADS--YYALLR-----------GGNKADECASAPPPGCQAPPVSECELPMIDVGCL--- :  56
OsGA3ox2     : MPT-PSHL---K----NPLCFDF---RAARR---------------VPETHAWP---------GLDDHPVVDGGGG--- :  41
```

```
                           *        100         *        120         *        140         *        160
AtGA2ox1     : -------------------ESKHALVKACEDFGFFKVINHG--V--SAELVSVLEHETVDFFSLPKSEK---TQV----- :  75
AtGA2ox2     : -------------------EAKTRIVKACEEFGFFKVVNHG--V--RPELMTRLEQEAIGFFGLPQSLK---NR------ :  87
AtGA2ox3     : -------------------DAKTQIVKACEEFGFFKVINHG--V--RPDLLTQLEQEAINFFALHHSLK---DK------ :  83
AtGA2ox4     : -----------------RSQVSMQIVKACESLGFFKVINHG--V--DQTTISRMEQESINFFAKPAHEK---KS------ :  73
AtGA2ox6     : ----------------DRSKLSEKIVKACEVNGFFKVINHG--V--KPEIIKRFEHEGEEFFNKPESDK---LR------ :  83
AtGA2ox7     : ----------TSGEEVKRKRCVKQMVAAAKEWGFFQIVNHG--I--PKDVFEMMLLEEKKLFDQPFSVK---VRERF--- : 108
AtGA2ox8     : ----------IDGAEEEREKCKEAIARASREWGFFQVINHG--I--SMDVLEKMRQEQIRVFREPFDKK---SK------ : 107
BdGA2ox1     : -----------------RGLLSRQVARACAEQGFFRAVNHGVLVPGPAPAARQLDAATAAFFALPAHEK---QR------ :  88
BdGA2ox10    : ---------GAGGSAAGRAAAAKAVAAACEEHGFFKVTGHG--V--ASGLLARVEAAAADFFALPQREK---EAAMAMAM :  99
BdGA2ox2     : ---------------WGRAELSRQMVEAFAERGFFKAVNHG--V--PPRAPATLDAATAAFFERPAPEK---QA------ :  82
BdGA2ox3     : -------------------GAPRAIADACERFGFFKLVNHG--V--ATDAMDRLESEAVTFFSQPQADK---DR------ :  84
BdGA2ox4     : -------------------GAADDVVRACERFGFFSVVNHG--V--ARGVVERLEHEAALFFSWPQADK---DA-SA--- :  86
BdGA2ox5     : LLDLESDQQTRNNGGSSSAACRAAMVRAASEWGFFQVTNHG--V--PQPLLDELHGQQLRAFRRPFHRK--LAP------ : 122
BdGA2ox6     : ---ITTGGEGGASSMRKKKACADAMARAASEWGFFQVTNHG--V--GKELLEEMRKEQARLFRLPFETK---RK------ : 116
BdGA2ox7     : -----------RRSGPGRAAAARALVAACEEQGFFKVTGHG--V--APALVRALDAAAAAFFALPQAEK---EA--A--- :  89
BdGA2ox8a    : -------------------GAARAVAEACRGVGFFRATNHG--V--PSSLAATLEADAMAFFALPDKDK---QSTT---- :  93
BdGA2ox8b    : -------------------GAARAVADACRGVGFFRATNHG--I--PSSLAAALEARAMAFFALPHEDK--VDA------ :  87
BdGA2ox9     : --------TTGGGSAEERAACAAAIAGAAEDWGFFQVVNHG--V--KQELLEAMRREQTRLFRLPFEAK---AT------ : 124
HvGA2ox1     : ---------------RGRGALSRQVARACAEQGFFRAVNHG--V-PPAGPPARLDAATSAFFALAAHDK---QR------ :  91
HvGA2ox10    : -----------RSGSEGRAAAARAVAAACEEHGFFKVTGHG--V--PAELLARVENAAAAFFALSQRDK---EAAMSA-- :  95
HvGA2ox2     : ---------------AGRAEMSRQMVEAFAERGFFKAVNHG--V--PPRASARLDAASAAFFARPAEEK---QE------ :  88
HvGA2ox3     : -------------------GAPRAIADACERFGFFKLVNHG--V--ALDAMDRLESEAVRFFSLPQADK---DR------ :  84
HvGA2ox4     : -------------------GAAADVVRACERFGFFSVVNHG--V--PAGVVDRLEAEAVRFFASTQAEK---DA------ :  84
HvGA2ox6     : ----------RSGDARERKACADAMAAAASEWGFFQVINHG--V--GRQLLEEMRREQARLFRLPFDTK---EK------ : 110
HvGA2ox7     : -------------PGPGRDAAARALVSACEEQGFFKVTGHG--V--PLELVRAVEAAAAEFFALPQAEK---EA--A--- :  87
HvGA2ox8     : -------------------GAAAAVADACRAVGFFRATNHG--V--PAALTDALEERAASFFALPHKDKMDASA------ :  85
HvGA2ox9     : --------TTGGGGEEERAACAAAIASAAAEWGFFQVVNHG--V--KQELLEAMRREQVRLFRLPFEAK---AT------ : 115
OsGA2ox1     : -----------------RGAVARQVATACAAHGFFRCVGHG--VPAAAPVAARLDAATAAFFAMAPAEK---QR------ :  97
OsGA2ox10    : ---------------ACRADAARAVVAACEGHGFFKVTGHG--V--PAGLLARVEAAAAAFFAMAQPEK---EA--AAAA :  92
OsGA2ox2     : ---------------AGRGELSRQVARACAGSGFFRAVNHG--V--PPRVSAAMDAGAAAFFARAGAEK---QL------ :  94
OsGA2ox3     : -------------------GAARAVVDACERYGFFKVVNHG--V--ATDTMDKAESEAVRFFSQTQPDK---DR------ :  84
OsGA2ox4     : -------------------GAAGEVVRACERFGFFKVVSHG--V--GEGVVGRLEAEAVRFFASPQAAK---DA------ :  85
OsGA2ox5     : --------------MEAEQVCRADMVRAASEWGFFQVTNHG--V--PQALLRELHDAQVAVFRRPFQEK-VTERLLG--- : 108
OsGA2ox6     : ----------TRGGAGERKACAGAMARAASEWGFFQLTNHG--V--GRELMEEMRREQARLFRLPFETK---EK------ : 115
OsGA2ox7     : --------------GPARAAEARALVAACEEQGFFRVTGHG--V--PPGLVRAAEAAAARFFALPQPDK---EA--A--- :  86
OsGA2ox8     : -------------------GAAAAVADACRTLGFFKATNHG--V--PAGLADALESSAMAFFALPHQEK--LDM------ :  87
OsGA2ox9     : TGAAAAAAVGQQHQAEERAACAAAIAAAAAEWGFFQVVNHG--V--AQELLEAMRREQARLFRLPFEAK---SS------ : 125
TaGA2ox-A1   : ---------------RGRGALSRQVARACTEQGFFRAVNHG--V-PPAGPAARLDAATSAFFALAAHDK---QR------ :  89
TaGA2ox-A10  : -----------GSGSDGRAAAARAVALACEEHGFFKVTGHG--V--PAELLTRVENAAAAFFALSQRDK---EA------ :  91
TaGA2ox-A11  : ----------ASRDSRESKACADAMARAASEWGFFQVVNHG--V--GRDLLEEMRREQAKLFRLPFRTK---DK------ : 116
TaGA2ox-A3   : -------------------GAPRAIADACERFGFFKLVNHG--V--PADTMDRLESEAVRFFSLPQADK---DR------ :  84
TaGA2ox-A4   : -------------------GAAADVVRACERYGFFSVVNHG--V--PAGMVDRLEAEAVRFFASSQADK---DA------ :  84
TaGA2ox-A6   : ----------TSGDAGERKACADAMARAASEWGFFQVINHG--V--GRELLEEMRREQARLFRLPFDTK---EK------ : 115
TaGA2ox-A7   : -------------PGPGRAAAARALVTACEEQGFCKVTGHG--V--PPELVRAVEAAAAEFFALPQADK---EA--A--- :  87
TaGA2ox-A8   : -------------------GAAAAVADACRGVGFFRVTNHG--V--PAALADALEERAAAFFALPHKDKLEASA------ :  85
TaGA2ox-A9   : --------TACGGGTEERAACAAAIASAAAEWGFFQVVNHG--V--KQELLEAMRREQVRLFRLPFEAK---AT------ : 115
TaGA2ox-B1   : ---------------HGRGALSRQVARACAEQGFFRAVNHG--V-PPAGPAARLDAATSAFFALAAHDK---QR------ :  89
TaGA2ox-B10  : -----------GSGSDGRAAAGRAVAAACEEHGFFKVTGHR--V--PAELLARVENAAAAFFALSQRDK---EA------ :  91
TaGA2ox-B11  : ----------ASRDARESKACAEAMARAASEWGFFQVVNHG--V--GRELLEEMRREQARLFRLPFGTK---DK------ : 116
TaGA2ox-B12  : ----------MMGDARERGVCMDAMASAASEWGFFQVINHG--V--GQELLEEMRREQARLFHQPFNIK---EK------ : 112
TaGA2ox-B13  : ----------TSGDARERDVCADAMASAASEWGFFQVINHG--V--GRELLEEMRREQAMLFHQPFDTK---EK------ : 108
TaGA2ox-B2   : ---------------AGRAELSGQMVEAFAERGFFKAVNHG--V--PPRVSARLDAASAAFFARPAHEK---QE------ :  85
TaGA2ox-B3   : -------------------GAPRAIADACERFGFFKLVNHG--V--PADTMDRLESEAVRFFSLPQADK---DR------ :  84
TaGA2ox-B4   : -------------------DAAADVVRACERFGFFSVVNHG--V--PAGVVDRLEAEAVRFFASSQAEK---DA------ :  84
TaGA2ox-B6   : ----------TSGDAWERKACADAMACAASEWGFFQVINHG--V--GRELLEEMRREQARLFRLPFDTK---EK------ : 115
TaGA2ox-B7   : -------------PGPGRAAAARALVSACEEQGFFKVTGHG--V--PPELVRAAEAAAAEFFALPQAEK---EA------ :  86
TaGA2ox-D1   : ---------------RGRGALSRQVARACAEQGFFRAVNHG--V-PPAGPPARLDAATSAFFALAAHDK---QR------ :  89
TaGA2ox-D10  : -----------GRGSEGRAAAARAVAAACEEHGFFKVTGHG--V--PAELLARVENAAAAFFALSQRDK---EA------ :  91
TaGA2ox-D11  : ----------TSRDARESKACADAMARAASEWGFFQVVNHG--V--GRELLEEMRREQTRLSRLPFGTK---DK------ : 116
TaGA2ox-D2   : ---------------AGRAELSRQMVEAFAERGFFKAVNHG--V--PPRVSARLDAASAAFFARPAEEK---QQ------ :  86
TaGA2ox-D3   : -------------------GAPRAIADACERFGFFKLVNHG--V--PADTMDRLESEAVRFFSLPQADK---DR------ :  84
TaGA2ox-D4   : -------------------DAAADVVRACERFGFFSVVNHG--V--PAGVVDRLEAEAVRFFASSQAEK---DA------ :  84
TaGA2ox-D6   : ----------TSGDAGERKACADAMATAASEWGFFQVINHD--V--GRELLEEMRREQARLFRLPFDTK---EK------ : 112
TaGA2ox-D7   : -------------RQSARNIQRLYLYQPSGGPGFFKVTGHG--V--PPELVRAAEAAAAEFFALPQAEK---EA--A--- : 101
TaGA2ox-D8   : --------------------AAAAVEQACRSVGIFRAINHG--V--PTALTDALEAGAAAFFALPHKKK---LEASA--- :  85
TaGA2ox-D9   : --------TACGGGTEERAACAAAIASAAAEWGFFQVVNHG--V--KQELLEAMRREQVRLFRLPFEAK---AT------ : 115
OsGA3ox2     : -GGED----AVPVVDVGAGDAAARVARAAEQWGAFLLVGHG--V--PAALLSRVEERVARVFSLPASEK---MRAVR--- : 106
```

```
                         *        180         *        200         *        220         *        240
AtGA2ox1    : ---A------G-----YPFGYGNSKI-GRNGDVGWVEYLLMNANHDS---GSGPLFPSLLK------------------ : 118
AtGA2ox2    : ---A------GPP---EPYGYGNKRI-GPNGDVGWIEYLLLNA-NPQ---LSSPKTSAVFR---------------Q--- : 132
AtGA2ox3    : ---A------GPP---DPFGYGTKRI-GPNGDLGWLEYILLNA-NLC---LESHKTTAIFR---------------H--- : 128
AtGA2ox4    : ---V------RPVN--QPFRYGFRDI-GLNGDSGEVEYLLFHTNDPA---FRSQLSF---------------------- : 115
AtGA2ox6    : ---A------GPA---SPFGYGCKNI-GFNGDLGELEYLLLHA-NPT---AVADKSETISH---------------D--- : 128
AtGA2ox7    : ---S------DLSK--NSYRWGNPSA-TSPAQYSVSEAFHIILSEVS---RISD------------------------- : 147
AtGA2ox8    : ---------SEKFSAGSYRWGTPSA-TSIRQLSWSEAFHVPMTDIS---DNK-------------------------- : 146
BdGA2ox1    : ---ARGVQGQGPPS--SPLGYGCRTI-GFSGDVGELEYLLLHANDPA---YKAASIHAH-------------------- : 138
BdGA2ox10   : APAA------AAAGS-SPFGYASKRI-GSNGDLGWVEYLLLGVTAAGAPLPVPSS--------------------A--- : 147
BdGA2ox2    : ---A------GPP---DPLGYGSRSI-GSHGDVGELEYLTLHA-DPA---AVARRAVAIDR---------------E--- : 127
BdGA2ox3    : ---S------GPA---YPFGYGSKRI-GLNGDMGWLEYLLLAVDDSA---SLSGA---------------------V--- : 124
BdGA2ox4    : ---S------GPA---DPFGYGSKRI-GRNGDMGWLEYLLLAI-DRE---SLSKASP-------------------A--- : 127
BdGA2ox5    : ---GDGDDEDQLLLSPEAYRWGNPTA-TCLHQLSWSEAYHVPITTPS---PETK------------------------- : 169
BdGA2ox6    : ---A------GLLN--GSYRWGNPTA-TSLRQLSWSEAFHVPL-ASI---SGRDCDY-------------------G--- : 158
BdGA2ox7    : ---A------G-----RPVGYASKRI-GTAGDLGWIEYLLLCVAPGAGQALPVPASASFSTLPCAAAAAAAAASLSGSEP : 154
BdGA2ox8a   : ---TTTTPAPGPGSARPSLGYGSRRI-GSNGDVGWLEYLILSLGSNS----------------------------V--- : 137
BdGA2ox8b   : ---T------IAAAARRPFGYGSRSI-GSNGDVGWLEYLLLSLGSNSNSSSIPGASS---------------------- : 134
BdGA2ox9    : ---A------GLLN--HSYRWGTPTA-TSPAQLSWSEAFHVPLAAVS---GDPPCNY-------------------G--- : 167
HvGA2ox1    : ---A------GPP---SPLGYGCRSI-GFNGDVGELEYLLLHA-NPA---AVAHRASSIDT---------------N--- : 136
HvGA2ox10   : ---A------TPG---SPFGYASKRI-GNNGDLGWVEYLLLGV-TAAGAEAGAPLSVPEEVVPS------------A--- : 146
HvGA2ox2    : ---A------GPP---DPLGYGSRSI-GSHGDVGELEYLILHT-DPE---VVARKARAVDR---------------D--- : 133
HvGA2ox3    : ---S------GPA---YPFGYGSKRI-GLNGDMGWLEYLLLAV-DSA---SLPAASA-------------------V--- : 125
HvGA2ox4    : ---S------GPA---DPFGYGSKRI-GRNGDMGWVEYLLLAI-DRD---TLSKASP-------------------A--- : 125
HvGA2ox6    : ---A------GLLN--GSYRWGNPTA-TSLRHLSWSEAFHVPL-ATI---SREDCDY-------------------G--- : 152
HvGA2ox7    : ---A------G-----RPLGYGSKRI-GVSGDLGWIEYLLLGVTPAG---ALPAASFASWTLPCAGAAVASSS---E--- : 143
HvGA2ox8    : ----------------RPFGYGSKNI-GCNGDVGWLEYILLSVRSGS---VAAAS------------------------ : 120
HvGA2ox9    : ---A------GLLN--DSYRWGSPTA-TSPRQLSWSEAFHVPL-AGI---SGSPCSY-------------------G--- : 157
OsGA2ox1    : ---A------GPA---SPLGYGCRSI-GFNGDVGELEYLLLHA-NPA---AVAHRARTIDA---------------M--- : 142
OsGA2ox10   : AAAA------VPG---SPFGYGSKRI-GCNGDLGWVEYLLLGVAAAA---AAPLPAHGE-----------------ASPS : 142
OsGA2ox2    : ---A------GRP---TPLGYGSRSI-GANGDVGELEYLILHA-SPD---AVARKASAIDR---------------E--- : 139
OsGA2ox3    : ---S------GPA---YPFGYGSKRI-GFNGDMGWLEYLLLALDDAS---LADACTV---------------------- : 125
OsGA2ox4    : ---H------GPA---SPFGYGSKRI-GRNGDMGWLEYLLLAI-DGA---SLSRSSP-------------------A--- : 126
OsGA2ox5    : ---F------SP----ESYRWGTPTA-KCLEQLSWSEAYHIPMTTPR---PSTSIRA---------------------- : 148
OsGA2ox6    : ---A------GLLN--GSYRWGNPTA-TSLRHLSWSEAFHVPL-ASI---SGADCDF-------------------G--- : 157
OsGA2ox7    : ---A------G-----APLGYASKRI-GSAGDLGWIEYLLLCL-APAAAAAALPCAATSPT------------------ : 131
OsGA2ox8    : ---S------GPA---RPLGYGSKSI-GSNGDVGWLEYLLLSAGAAS---SGGAA------------------------ : 126
OsGA2ox9    : ---A------GLLN--DSYRWGTPTA-TSLRQLSWSEAFHLPL-AGI---SGKSCNY-------------------G--- : 167
TaGA2ox-A1  : ---A------GPP---SPLGYGCRSI-GFNGDVGELEYLLLHA-NPA---AVAHRASSIDTN----------------- : 134
TaGA2ox-A10 : ---AMSAATPG-----SPFGYASKRI-GNNGDLGWVEYLLLGV-SAA---TSGPLSVPEGVVPSA-------------- : 143
TaGA2ox-A11 : ---A------GLLN--GSYRWGNPTA-TSLGQLSWSESFHVPL-PSI---SREDCDY-------------------G--- : 158
TaGA2ox-A3  : ---S------GPA---YPFGYGSKRI-GLNGDMGWLEYLLLAVDAAS---LSAACA--------------------V--- : 125
TaGA2ox-A4  : ---S------GPA---DPFGYGSKRI-GRNGDMGWVEYLLLAI-ERD---ALSKASP-------------------A--- : 125
TaGA2ox-A6  : ---A------GLLN--GSYRWGNPTA-TSLRHLSWSEAFHVPL-ASI---SREDCDY-------------------G--- : 157
TaGA2ox-A7  : ---A------G-----RPLGYGSKRI-GVSGDLGWIEYLMLGV-TPG---ALPASFASWTLPCAADAVAASLS---E--- : 142
TaGA2ox-A8  : ----------------RPLGYGSKSI-GCNGDVGWLEYILLSVGSGS---VAAAS------------------------ : 120
TaGA2ox-A9  : ---A------GLLN--DSYRWGSPTA-TSPRQLSWSEAFHVPL-AGI---SGSPCSY-------------------G--- : 157
TaGA2ox-B1  : ---A------GPP---SPLGYGCRSI-GFNGDFGELEYLLLHA-NPA---AVAHRASSIDTN----------------- : 134
TaGA2ox-B10 : ---AMSAATPG-----SPFGYASKRI-GNNGDLGWVEYLLLGV-TAS---TGGPLSVPEGVVPSA-------------- : 143
TaGA2ox-B11 : ---A------GLLN--GSYRWGNPTA-TSLGQLSWFESFHVPL-PSI---SREDCDR-------------------G--- : 158
TaGA2ox-B12 : ---A------GLLN--GSYRWGTPTA-TSLRQLSWSEAFHVPLASIS---GEDDCGH-------------------A--- : 155
TaGA2ox-B13 : ---A------GLLN--GSYRWGNATA-TSLRQLSWSEAFHVPLASIS---EEDA------------------------- : 147
TaGA2ox-B2  : ---A------GPP---DPLGYGSRSI-GSHGDVGELEYLILHT-DPE---VVARKARAIDR---------------D--- : 130
TaGA2ox-B3  : ---S------GPA---YPFGYGSKRI-GLNGDMGWLEYLLLAVESAS---LSGACTV---------------------- : 125
TaGA2ox-B4  : ---S------GPA---DPFGYGSKRI-GRNGDMGWVEYLLLAI-ERD---ALSKASP-------------------A--- : 125
TaGA2ox-B6  : ---A------GLLN--GSYRWGNPTA-TSLRHLSWSEAFHVPL-ASI---SREDCDY-------------------G--- : 157
TaGA2ox-B7  : ---------AAG---RPLGYGSKRI-GVSGDLGWIEYVMLGVTPAG---ALPAASFTSWTLPCAGAAV-------ASLS : 142
TaGA2ox-D1  : ---A------GPP---NPLGYGCRSI-GFNGDVGELEYLLLHA-NPA---AVAHRASSIDTN----------------- : 134
TaGA2ox-D10 : ---AMSAATPG-----SPFGYASKRI-GNNGDLGWVEYLLLGV-TAA---TGGPLSVPEGVVPSA-------------- : 143
TaGA2ox-D11 : ---A------GLLN--GSYRWGNPTA-TSLGQLSWSESFHVPL-PSI---SREDCDY-------------------G--- : 158
TaGA2ox-D2  : ---A------GPP---DPLGYGSRSI-GSHGDVGELEYLILHT-DPD---VVARKARAIDR---------------D--- : 131
TaGA2ox-D3  : ---S------GPA---YPFGYGSKRI-GLNGDMGWLEYLLLAVDSAS---LSGACAV---------------------- : 125
TaGA2ox-D4  : ---S------GPA---DPFGYGSKRI-GRNGDMGWVEYLLLAI-ERD---TLSKASP-------------------A--- : 125
TaGA2ox-D6  : ---A------GLLN--GSYRWGNPTA-TSLRHLSWSEAFHVPL-ASI---SREDCDY-------------------G--- : 154
TaGA2ox-D7  : ---A------G-----RPLGYDSKRI-GVSGDLGWIEYLMLGVTPAG---ALPAASFASWTLPCAGAAVASLS---E--- : 157
TaGA2ox-D8  : ----------------NPLGYGSKSI-GRNGDVGWLEYILLSV-GSG---PVAAA---------------------S--- : 120
TaGA2ox-D9  : ---A------GLLN--DSYRWGSPTA-TSPRQLSWSEAFHVPL-AGI---SGTPCSY-------------------G--- : 157
OsGA3ox2    : ---G------PG----EPCGYGSPPISSFFSKLMWSEGYTF---SPS---SLRSELRRLWPKSG------------D--- : 152
```

```
                          *       260         *       280         *       300         *       320
AtGA2ox1     : -SPGTF-RNALEEYTTSVRKMTFDVLEKITDG-LGI---KPRNT-LSKLVSDQN----TDSILRLNHYP--PCPLSN--- : 182
AtGA2ox2     : -TPQIF-RESLEEYMKEIKEVSYKVLEMVAEE-LGI---EPRDT-LSKM-LRDE--K-SDSCLRLNHYP--AAEEEA--- : 196
AtGA2ox3     : -TPAIF-REAVEEYIKEMKRMSSKFLEMVEEE-LKI---EPKEK-LSRL-VKVK--E-SDSCLRMNHYP--EKEETP--- : 192
AtGA2ox4     : -------SSAVNCYIEAVKQLAREILDLTAEG-LHV----PPHS-FSRLISSVD----SDSVLRVNHYP--PSDQFF--- : 173
AtGA2ox6     : -DPFKF-SSATNDYIRTVRDLACEIIDLTIENLWGQ---KSSE--VSEL-IRDV--R-SDSILRLNHYP--PAPYAL--- : 192
AtGA2ox7     : -DRNNL-RTIVETYVQEIARVAQMICEILGKQ-VNV----SSEY-FEN--IFEL----ENSFLRLNKYH--PSVFGS--- : 208
AtGA2ox8     : -DFTTL-SSTMEKFASESEALAYMLAEVLAEK-------SGQNSSFFK--ENCV--R-NTCYLRMNRYP--PCPKPS--- : 207
BdGA2ox1     : -DPKHF-SCVVNEYVEAVKQLACDILDLLGEG-LGL---EDPRS-FSKLITEPD----SDSLLRINHYP--PSCTVHKLD : 205
BdGA2ox10    : -SLCAF-RELLDEYTVAVRRMTCEVLELMAEA-LGM----EKDE-FTRL-VLEE--D-SDSMLRVNHYPPAPRPELK--- : 212
BdGA2ox2     : -DPSRF-SEAVNEYVGAVRRLACQILDLLGEG-LGL---EDPTA-LSKIITVSD----SDSIIRINHYP--PSSAAA--- : 191
BdGA2ox3     : -PSGSAFRRALNEYIGAVRKVAVRVMEAMAEG-LGI---APLDA-LSKM-VTAA--G-SDQVFRVNHYP--PCAALQ--- : 189
BdGA2ox4     : -PSSSL-RDAINEYVGAMRGLARTVLEMVAEG-LGV---SPRGA-LADM-VTGE--A-SDQVFRLNHYP--PCPLLQ--- : 191
BdGA2ox5     : -TTTAT-RRVIHEVSAAMSKLARRILSVLAAE-HG----PPPET-----------ETTTCFLRLNRYP--QAPGSG--- : 225
BdGA2ox6     : -KLSSL-RGVMQEVADAMSRVADAVAGALAEK-LGQ---EAGSA---LFPAGCD--G-TTCFLRLNRYP--ACPFAP--- : 221
BdGA2ox7     : ESSCPL-RGLLEEYAAALRRMACEVLELMAEG-LGL---APSDA-ISRL-VSDG--G-SDNMLRVNHYP--PRPELQ--- : 219
BdGA2ox8a    : -LPASL-RLALHEYTRAVRELSGRVLELMAEG-LGIVGETERGV-LRRMVAGSE-----EELVRVNHYP--PPTTER--- : 203
BdGA2ox8b    : -LPPSL-RAALEEYTGAVRNASGKVLELMAEG-LGM---QERCA-LRRM-VDGS----EEELVRVNHYP--PTKEED--- : 197
BdGA2ox9     : -QLTTL-RDVTQEVATAMSKLANTLARVLAER-LGH-----AGE---RFPEGCD--E-RTCFLRLNRYP--PCPLSP--- : 228
HvGA2ox1     : -DPSRF-SSVVNEYVEAVKQLACDILDLLGEG-LGL---EDPRL-FSKL-VTEA--D-SDSLLRINHYP--PSCTVHKLD : 203
HvGA2ox10    : -SLCSF-RDLLNEYTVAVRRMTCQLLELMAEG-LGM---EDRDA-FTRL-VLHK--E-SDSMLRVNHYP--PRPELK--- : 210
HvGA2ox2     : -DPSRF-SEAVNEYVQAVRHLACRILDMLGEG-LGL---RDPAS-LSRLITTTD----GDSLVRINHYP--ASAAGD--- : 197
HvGA2ox3     : -PSCALFRAALNEYIAAVRKVAVRVMEAMAEG-LGI---AQADA-LSAM-VAAE--G-SDQVFRVNHYP--PCHALQ--- : 190
HvGA2ox4     : -PSSAL-REAINAYVSAMRGLARTVLEMVAEG-LGV---SPRGA-LADM-VTGE--A-SDQVFRVNHYP--PCPLLQ--- : 189
HvGA2ox6     : -KLSSL-REVMQEVADAMSCVADTVAGALAEE-LGH---AAGGG-ESTFPAGCD--G-TTCFLRLNRYP--ACPFAP--- : 217
HvGA2ox7     : -HPCPL-RDLLEEYAAAVRRMACGVLELMAEG-LGI---GPADA-LSRL-VSDG--E-SDNMLRVNHYP--PRPEMQ--- : 207
HvGA2ox8     : -LPPSL-RAALEEYTDAVREVGARVLELMADG-LGV-AEEHRGV-LRRMVAPDDAGG-ADEMVRVNHYP--PCPSSL--- : 189
HvGA2ox9     : -ELTSI-RDVTQEVAKAMSKLANTLARVLAES-LGH---TAGQG----FPEGCD--E-STCFLRLNRYP--ACPFSP--- : 219
OsGA2ox1     : -DPSRF-SAIVNEYIEAMKKLACEILDLLGEG-LGL---KDPRY-FSKL-TTNA--D-SDCLLRINHYP--PSCNIHKLD : 209
OsGA2ox10    : PSYGSF-RDILNEYVVAVRAMMWEVLKLMAEG-LGL---KEKDA-LVRL-VSHE--E-SDSVLRVNHYP--PHPELK--- : 207
OsGA2ox2     : -DPRRF-SQVVNDYVEAVRQLACHVLDLLGEG-LGL---RDPTS-LTRLITATD----NDSLIRINHYP--PSCAAA--- : 203
OsGA2ox3     : -PSCAVFRAALNEYISGVRKVAVRVMEAMSEG-LGI---AQADA-LSAL-VTAE--G-SDQVFRVNHYP--PCRALQ--- : 190
OsGA2ox4     : -PSSSL-RDAANKYVGAMRGMARTVLEMVAEG-LGV---APRGA-LADM-VVGDGAA-SDQILRLNHYP--PCPPLL--- : 192
OsGA2ox5     : -------RAVIEEVSRAMYELAQKLAEILMRGLPGA---GEGET-M----VTTR--E-ETCFLRLNRYP--PCAMAM--- : 205
OsGA2ox6     : -DLTSL-RGVMQEVAEAMSRVANTVAAALAEELTGR--GGGGASAAPWFPAGCD--E-TTCFLRLNRYP--ACPFAA--- : 225
OsGA2ox7     : -PPCPL-RELLREYSAAVRRVACGVLELMAEG-LGV---GPADA-LARL-VARE--D-SDSILRVNHYP--PRPDQL--- : 195
OsGA2ox8     : -LPAAL-RAAVEAYTGAVRGVGCRVMELMAEG-LGLGASEEGRCVLRRMVVGCE--G-SDEMLRVNHYP--PCLLPP--- : 195
OsGA2ox9     : -DLTSL-RDVTREVADAMSRLARALARVLAESLLGH---AAGER----FPEGCD--D-ATCFLRLNRYP--PCPFPP--- : 230
TaGA2ox-A1   : -DPSRF-SSVVNEYVEAVKQLACDILDLLGEG-LGL---EDPRL-FSKLITETD----SDSLLRINHYP--PSCTVH-KL : 200
TaGA2ox-A10  : -SLCSF-RDLLNEYTVAVRRMTCQALELMAEG-LGM---DDRDA-FTRLVLHKE----SDSMLRVNHYP--PRPELK--- : 207
TaGA2ox-A11  : -KLNSL-RGVMQVVADAMSRVAYTVAGTLAEN-LGH---EAGGG-EPVFPAGCD--G-TTCFLRLNRYP--ACPFAA--- : 223
TaGA2ox-A3   : -PSCALFRAALNEYIAAVRKVAVRVMEAMAEG-LGI---APVDA-LSAM-VTAE--G-SDQVFRVNHYP--PCHALQ--- : 190
TaGA2ox-A4   : -PTSAL-RDAINDYVGAMRGLARTVLEMVAEG-LGV---SPRGA-LADM-VTGD-AA-SDQVFRVNHYP--PCPLLQ--- : 190
TaGA2ox-A6   : -KLSSL-RAVMQEVADAMSRVADTVAAALAED-LGH---DAWGG-EPAFPAGCD--G-TTCFLRLNRYP--ACPFAP--- : 222
TaGA2ox-A7   : -PPCPL-RDLLEEYAAAVRRMACGVLELMAEG-LGV---APADA-LSRL-VSDA--E-SDSMLRVNHYP--PRPELQ--- : 206
TaGA2ox-A8   : -LPASL-RAALEEYTDAVREVGARVLELMADG-LGV-AEEHRGV-LRRM-VAPD--E-ADEMVRVNHYPPCPCPLAA--- : 188
TaGA2ox-A9   : -ELTSI-RDVTQEVANAMSKLANTLARVLASS-LGH---TAGQR----FPEGCD--E-RTCFLRLNRYP--PCPFSP--- : 219
TaGA2ox-B1   : -DPSRF-SSVVNEYVEAVKQLACDILDLLGEG-LGL---EDPRL-FSKLITETD----SDSLLRINHYP--PSCTVH-KL : 200
TaGA2ox-B10  : -SLCSF-RDLLNEYTVAVRRMTCQVLELMAEG-LRM---EDRDA-FTRLVLHKE----SDSMLRVNHYP--PRPELK--- : 207
TaGA2ox-B11  : -KLSTL-RGVMQEVADAMSQVAGTVAGMLAEN-LGH---EAGGE--SAFPAGCD--G-TTCFLRLNRYP--ACPFAA--- : 222
TaGA2ox-B12  : -ELSSL-RGVMQEVAAAMSRVADTVAGTLAKN-LGH---ESTFP----AAAGCD--G-TTCFLRLNRYP--ACPFAA--- : 217
TaGA2ox-B13  : -KLSSL-RGMMQEVADAMSRVAETVAGTLAKN-LG----QAGST-FPAA-AGCD--G-TTCFLRLNRYP--ACPFAP--- : 210
TaGA2ox-B2   : -DPSRF-SEAVNVYVQAVRHLACQILDLLGEG-LGL---RDPAS-LSRLVTTTD----SDSLVRINNYP--PSAASD--- : 194
TaGA2ox-B3   : -PSCALFRAALNEYIAAVRKVAVRVMEAMAEG-LGI---APVDA-LSAMVTAGG----SDQVFRVNHYP--PCHALQ--- : 190
TaGA2ox-B4   : -PTSAL-RDAINAYVGAMRGLARTVLEMVAEG-LGV---SPRGA-LADM-VTGD-VA-SDQVFRLNHYP--PCPLLQ--- : 190
TaGA2ox-B6   : -KLSSL-RGVMQEVADAMSHVADTVAAALAED-LGH---DAGGG-EPAFPAGCD--G-TTCFLRLNRYP--ACPFAP--- : 222
TaGA2ox-B7   : EPPCPL-RDLLEEYAAAVRRMACGVLELMAEG-LGV---APADA-LSRL-VSDA--E-SDSMVRVNHYP--PRPELQ--- : 207
TaGA2ox-D1   : -DPSRF-SSVVNEYVEAVKQLACDILDLLGEG-LGL---EDPRL-FSKLITETD----SDSLLRINHYP--PSCTVH-KL : 200
TaGA2ox-D10  : -SLCSF-RDLLNEYTEVVRRMTCQVLELMAEG-LGM---DDRDA-FTRLVLHKE----SDSMLRVNHYP--PRPELK--- : 207
TaGA2ox-D11  : -KLSTL-RGVMQEVADAMLRVADTVAGTLAEN-LGH---EAGGS-ESAFPAGCD--G-TTCFLRLNRYP--ACPFAA--- : 223
TaGA2ox-D2   : -DPSRF-SEAVNEYVQAVRHLACQILDLLGEG-LGL---RDPAS-ISRLVTTTD----SDSLVRINNYP--PSATGD--- : 195
TaGA2ox-D3   : -PSCALFRAALNEYIAAVRKVAVRVMEAMAEG-LGI---AAVDA-LSGM-VTAE--G-SDQVFRVNHYP--PCHALQ--- : 190
TaGA2ox-D4   : -PSTAL-RDAINAYVGAMRGLARTVLEMVAEG-LGV---SPRGA-LADM-VTGD--VASDQVFRLNHYP--PCPLLQ--- : 190
TaGA2ox-D6   : -KLSSL-RGVMQEVADAMSRVADTVAAVLAED-LGH---DAGGG-EPAFPAGCD--G-TTCFLRLNRYP--ACPFAP--- : 219
TaGA2ox-D7   : -PPCPL-RDLLEEYAAAVRRMACGVLELMAEG-LGI---APADA-LSRL-VADA--E-SDSMLRVNHYP--PRPELQ--- : 221
TaGA2ox-D8   : -LPPSL-RAALEEYTDAVREVGARVLELMAEG-LGL-AEENRGV-LRRV-VASD--E-ADKMVRVNHYPPWPCPLAA--- : 188
TaGA2ox-D9   : -ELASI-RDVTQEVANAMSKLANTLARVLAES-LGH---TAGQR----FPKGCD--E-RTCFLRLNRYP--PCPFSP--- : 219
OsGA3ox2     : -DYLLF-CDVMEEFHKEMRRLADELLRLFLRA-LGLTGEEVAGV-EAER-RIGE--R-MTATVHLNWYP--RCPEPR--- : 219
```

```
                       *       340         *       360         *       380         *       400
AtGA2ox1    : ------------------KKTNGGKNVIGFGEHTDPQIISVLR--SNNTSGLQINL-ND-------G-----SWISVPP- : 228
AtGA2ox2    : ----------------------EKMVKVGFGEHTDPQIISVLR--SNNTAGLQICV-KD-------G-----SWVAVPP- : 238
AtGA2ox3    : -----------------------VKEEIGFGEHTDPQLISLLR--SNDTEGLQICV-KD-------G-----TWVDVTP- : 233
AtGA2ox4    : -------------GEANLSDQSVSLTRVGFGEHTDPQILTVLR--SNGVGGLQVSN-SD-------G-----MWVSVSP- : 224
AtGA2ox6    : ----------------------SGVGQIGFGEHSDPQILTVLR--SNDVDGLEICS-RD-------G-----LWIPIPS- : 234
AtGA2ox7    : -------------------------EVFGLVPHTDTSFLTILS--QDQIGGLELE--NN-------G-----QWISVKP- : 246
AtGA2ox8    : -------------------------EVYGLMPHTDSDFLTILY--QDQVGGLQLIK-DN-------------RWIAVKP- : 245
BdGA2ox1    : HDGQCKLKGTACRAKAGNGGNPTGGGRIGFGEHSDPQILSLLR--ANDVDGLQVLL-PD--------VNGKEVWIQVPA- : 273
BdGA2ox10   : ----------------QQLQHGGNGRVTGFGEHTDPQIISVLR--SNATSGLEIAL-RD-------G-----TWVSVPP- : 260
BdGA2ox2    : -------------------------SSVGFGEHSDPQILSVLR--ANDVDGLQVLL-PD-------GRGQD-TWVQVPA- : 234
BdGA2ox3    : ---------------------GLGCSATGFGEHTDPQLVSVLR--SNGTSGLQIAL-QDG------G-----QWVSVPS- : 233
BdGA2ox4    : -------------------GLPPNCSVTGFGEHTDPQLVSILH--SNATAGLQIAL-HGD------GDAQ--KWVSVPP- : 240
BdGA2ox5    : --------------------GGHGHGVLGLCGHTDSDFLTILR-QDDHVGGLQLLL-DDKDGGR--------RWRTVRP- : 274
BdGA2ox6    : -------------------------DTFGLVPHTDSDFLTILC--QDQVGGLQLMK-DS-------------RWVAVKP- : 259
BdGA2ox7    : --------------------AGKNQLLTGFGEHTDPQIISVLR--SNGTSGLEIAS-PRD------G-----AWASVPP- : 264
BdGA2ox8a   : -----------------DEDDDGCVGITGFGEHTDPQLISLLR--SNRTAGYQILL-QEADEA---------RWVNVAP- : 253
BdGA2ox8b   : ----------------------CVAGMTGFGEHTDPQIISLLR--SNRTAGLQIKLQGPD------A-----PWVNVAP- : 241
BdGA2ox9    : -------------------------DAFGLVPHTDSDFLTVLC--QDQVGGLQLMK-GS-------------RWLAVKP- : 266
HvGA2ox1    : HDDQCKLKGIA-RTKAGNGANPGAGGRIGFGEHSDPQLLSLLR--ANDVDGLQVLL-PD--------VNGKDAWIQVPA- : 270
HvGA2ox10   : --------------------QHHGGRVTGFGEHTDPQIISVLR--SNATSGLEIAL-RD-------G-----DWVSVPP- : 254
HvGA2ox2    : -------------------GGVKAASSVGFGEHTDPQILSVLR--ANDVDGLQVLL-PD-------GRGED-AWVQVPA- : 246
HvGA2ox3    : ---------------------GLGCSATGFGEHTDPQLISVLR--SNGTSGLQIAL-QS-------G-----HWVSVPS- : 233
HvGA2ox4    : -------------------GLPPNCSVTGFGEHTDPQLVSILH--SNGTAGLQVAL-HD-------G-----RWVSVPP- : 234
HvGA2ox6    : -------------------------DTFGLVPHTDSDFLTILC--QDQVGGLQLM--KD-------A-----RWVAVKP- : 255
HvGA2ox7    : -----------------------GRLLMGFGEHTDPQIISVLR--SNGTSGLEICA-RD-------G-----EWTSVPP- : 248
HvGA2ox8    : --------------------AAGQRGVTGFGEHTDPQIISVLR--SNRTAGFQIML-PD-------G-----RWVPVAP- : 233
HvGA2ox9    : -------------------------DAFGLVPHTDSDFLTVLC--QDQVGGLQLMK-GS-------------RWVAVKP- : 257
OsGA2ox1    : HDDQCNIKSLV-STKASNGGNLMAGGRIGFGEHSDPQILSLLR--ANDVEGLQVFV-PDHEGK---E-----MWVQVPS- : 276
OsGA2ox10   : --------------------QQGHGRLTGFGEHTDPQIISVLR--SNDTSGLEISL-RD-------G-----SWASVPP- : 251
OsGA2ox2    : ------------AGDHKSGGGPAPTAAIGFGEHTDPQILSVLR--ANDADGLQLLLPDA-------AAAGDSVWVPVPP- : 261
OsGA2ox3    : ---------------------GLGCSVTGFGEHTDPQLVSVLR--SNGTSGLQIAL-RD-------G-----QWVSVPS- : 233
OsGA2ox4    : ------------------QNLMPNCSPTGFGEHTDPQLISILH--SNSTSGLQVAL-HHD------ADAGDHQWVTVPP- : 244
OsGA2ox5    : -------------------------GGFGLCPHTDSDLLTIVHQQQDTVGGLQLLK-G--------G-----RWVAVKP- : 245
OsGA2ox6    : -------------------------DTFGLVPHTDSDFLTVLC--QDQVGGLHLMK-DS-------------RWVAVRP- : 263
OsGA2ox7    : -------------------GGGGGPNLTGFGEHTDPQIISVLR--SNGAPGLEISL-RD-------G-----AWASVPHD : 241
OsGA2ox8    : ------------------GRDRDECGVTGFGEHTDPQIISVLR--SNCTAGLQILL-RGDYSSP--A-----RWVPVPP- : 246
OsGA2ox9    : ------------------------DDAFGLVPHTDSDFLTVLC--QDHVGGLQLMK-GS-------------RWVAVKP- : 269
TaGA2ox-A1  : DHDDQCKLKGVARTKAGSGGNPAAGGRIGFGEHSDPQLLSLLR--ANDVDGLQVLL-PD--------INGKDTWIQVPA- : 268
TaGA2ox-A10 : -------------LLQQQQQHGGGGRVTGFGEHTDPQIISVLR--SNATSGLEIAL-RD-------G-----AWVSVPP- : 258
TaGA2ox-A11 : -------------------------DFFGMVPHTDSDFLTILC--QDQVGGLQLI--KD-------S-----HWVAVKP- : 261
TaGA2ox-A3  : ---------------------GLGCSATGFGEHTDPQLISVLR--SNGTSGLQIAL-QN-------G-----QWVSVPS- : 233
TaGA2ox-A4  : -------------------GLPPNCSVTGFGEHTDPQLVSILH--SNGTPGLQVAL-HD-------G-----RWVSVPP- : 235
TaGA2ox-A6  : -------------------------DTFGLVPHTDSDFLTILC--QDQVGGLQLMK-DS-------------RWVAVKP- : 260
TaGA2ox-A7  : -----------------------GRLLTGFGEHTDPQIISLLR--SNGTSGLEICA-RD-------G-----SWAAVPP- : 247
TaGA2ox-A8  : ----------------------GQRGVTGFGEHTDPQIISVLR--SNRIGGLQIML-PD-------G-----RWVPVAP- : 230
TaGA2ox-A9  : -------------------------DAFGLVPHTDSDFLTVLC--QDQVGGLQLMK-GS-------------QWVAVKP- : 257
TaGA2ox-B1  : DHDDQCKLKGVARTKAGNGGNPAAGGRIGFGEHSDPQLLSLLR--ANDVDGLQVLL-PD--------INGKDVWIQVPA- : 268
TaGA2ox-B10 : ---------------LLQQQHGGGGRVTGFGEHTDPQIISVLR--SNATSGLEIAL-RD-------G-----AWVSVPP- : 256
TaGA2ox-B11 : -------------------------DSFGMVPHTDSDFLTILC--QDQVGGLQLI--KD-------S-----RWVAVKP- : 260
TaGA2ox-B12 : -------------------------DTLGMVPHTDSDFLTVLC--QDQVGGLEIMK-DS-------------HWVAVKP- : 255
TaGA2ox-B13 : -------------------------DTFGMMPHTDSDFLTVLC--QDQVGGLEIMK-DS-------------HWVAVKP- : 248
TaGA2ox-B2  : -------------------GGVKAASSVGFGEHSDPQILSVLR--ANDVDGLQVLL-PD-------GRGED-AWVQVPA- : 243
TaGA2ox-B3  : ---------------------GLGCSATGFGEHTDPQLISVLR--SNGTSGLQIAL-QN-------G-----QWVSVPS- : 233
TaGA2ox-B4  : -------------------GLPPNCSVTGFGEHTDPQLVSILH--SNGTPGLQVAL-HD-------G-----RWVSVPP- : 235
TaGA2ox-B6  : -------------------------DTFGLVPHTDSDFLTILC--QDQVGGLQLM--KD-------S-----RWVAVKP- : 260
TaGA2ox-B7  : -----------------------GRLLTGFGEHTDPQVISVLR--SNGTSGLEICA-RD-------G-----SWTAVPP- : 248
TaGA2ox-D1  : DHDDQCKLKGVARTKAGSGGNPAAGGRIGFGEHSDPQLLSLLR--ANDVDGLQVLL-PD--------INGKDTWIQVPA- : 268
TaGA2ox-D10 : ----------------LLQQHGGGGRVTGFGEHTDPQIISVLR--SNATSGLEIAL-RD-------G-----AWVSVPP- : 255
TaGA2ox-D11 : -------------------------DSLGMVPHTDSDFLTILC--QDQVGGLQLIK-DS-------------RWVAVKP- : 261
TaGA2ox-D2  : -------------------GGVKAASAVGFGEHSDPQILSVLR--ANDVDGLQVLL-PD-------GRGED-AWVQVPA- : 244
TaGA2ox-D3  : ---------------------GLGCSATGFGEHTDPQLISVLR--SNGTSGLQIAL-QN-------G-----QWVSVPS- : 233
TaGA2ox-D4  : -------------------GLPPNCSVTGFGEHTDPQLVSILH--SNGTPGLQVAL-HD-------G-----RWVSVPP- : 235
TaGA2ox-D6  : -------------------------DTFGLVPHTDSDFLTILC--QDQVGGLQLM--KD-------S-----RWVAVKP- : 257
TaGA2ox-D7  : -----------------------GRLLTGFGEHTDPQVISVLR--SNGTSGLEICA-RD-------G-----SWAAVPP- : 262
TaGA2ox-D8  : --------------------GQRGRGVTGFGEHTDPQVISLLR--SNRT-GLQIML-PD-------G-----RWVPVAP- : 231
TaGA2ox-D9  : -------------------------DAFGLVPHTDSDFLTVLC--QDQVGGLQLMK-GS-------------RWVAVKP- : 257
OsGA3ox2    : -------------------------RALGLIAHTDSGFFTFVL--QSLVPGLQLFR-------RGPD-----RWVAVPA- : 259
```

```
                         *        420         *        440         *        460         *        480
AtGA2ox1     : -DHTSFFFNVGDSLQVMTNGRFKSVRHRV-LA--N-CKKSRVSMIYFAGPSLTQRI-APLTCLI-------DNEDERLYE : 295
AtGA2ox2     : -DHSSFFINVGDALQVMTNGRFKSVKHRV-LA--D-TRRSRISMIYFGGPHLSQKI-APLPCLV-------PEQDDWLYK : 305
AtGA2ox3     : -DHSSFFVLVGDTLQVMTNGRFKSVKHRV-VT--N-TKRSRISMIYFAGPPLSEKI-APLSCLV-------PKQDDCLYN : 300
AtGA2ox4     : -DPSAFCVNVGDLLQVMTNGRFISVRHRA-LT--Y-GEESRLSTAYFAGPPLQAKI-GPLSAMV------MTMNQPRLYQ : 292
AtGA2ox6     : -DPTCFFVLVGDCLQALTNGRFTSVRHRV-LA--NTAKKPRMSAMYFAAPPLEAKI-SPLPKMV-------SPENPRRYN : 302
AtGA2ox7     : -CLEALTVNIGDMFQALSNGVYQSVRHRV-IS--P-ANIERMSIAFFVCPYLETEI-DCF-------------GYPKKYR : 307
AtGA2ox8     : -NPKALIINIGDLFQAWSNGMYKSVEHRV-MT--N-PKVERFSTAYFMCPSYDAVIECS--------------SDRPAYR : 306
BdGA2ox1     : -DSSAFFVNVGDLLQALTNGKLLSVRHRV-IA--S-ACRPRLSTIYFAAPPLHARI-SALPETV-------TAGSPRRYR : 340
BdGA2ox10    : -DHTSFFVNV------LTNGRFRSVRHRV-MV--N-SVRSRVSVIFFGGPAPGKTL-APLPRMV----GE---GGSSRYR : 321
BdGA2ox2     : -DPAAFFINVGDLLQALTNGRLVSIRHRV-MA--S-TSKPRLSTIYFAAPALHALI-SALPETV-------TSDAPRRYR : 301
BdGA2ox3     : -DRDALFFVNVGDSLQVLTNGRFKSVKHRV-VA--N-SLKSRVSLIYFGGPPLTQRI-APLPQLL----GE---GEQSLYT : 300
BdGA2ox4     : -NRDAFFVNVGDSLQVLTNGRLKSVRHRV-VA--GSGRKSRVSMIYFGGPPVAQRI-APLPQLL----QAE--GQLPIYR : 309
BdGA2ox5     : -NPGALTVNVGDLLQAWTNDVYASVEHRV-VA--R-PDRERFSVAFFLCPSYDTLIRPPLPGKT---------TTSQVYR : 340
BdGA2ox6     : -HPDALIVNIGDLFQAWSNNRYKSVEHKV-VA--N-AKAERFSVAYFLCPSYDAPV-GTC-------------GEPSPYR : 320
BdGA2ox7     : -DADSFFINVGDVLQVLTNGRFRSVKHRV-VV--N-SERPRMSMIFFGGPPPGERL-APLRELL----GDD--GGRSRYR : 332
BdGA2ox8a    : -DPDSFFVNVGDTLQVLTNGRFRSVKHRVLVAPERGGKASRLSVIYFGGPAPAQRI-APLPELM-------REGERSLYR : 324
BdGA2ox8b    : -DPDSLFVNVGDCLQVLTNGRFRSVKHRV-VAPEG-AQASRLSVIYFGGPAPAQRI-APLPELM-------REGEQSLYR : 310
BdGA2ox9     : -IPNALIVNIGDLFQAWSNNRYRSVEHKV-VT--N-ATTERYSVAYFLCPSYDSPI-GAC-------------EEPSPYR : 327
HvGA2ox1     : -DSSAYFVNVGDLLQALTNGRLVSIRHRV-IA--S-ACRPRLSTIYFAAPPLHARI-SALPEMV-------TASSPRRYR : 337
HvGA2ox10    : -DQTSFFVNVGDALQVLTNGRFRSVRHRV-MV--N-NIQPRVSVIFFGGPPPRETL-APLPQLV----GE---GGRSRYK : 321
HvGA2ox2     : -DPAAFFINVGDLLQALTNGKLVSIRHRV-MA--S-SSKPRLSAIYFAAPALHERI-SAFPETV-------TAAAPRRYR : 313
HvGA2ox3     : -DRDAFFVNVGDSLQVLTNRRFKSVKHRV-VA--N-SLKSRVSMIYFGGPALTQRI-APLPQLL----RE---GEQSLYK : 300
HvGA2ox4     : -NRDAFFVNVGDSLQVLTNGRLRSVRHRV-VA--GNGLKSRVSMIYFAGPPLAQRI-APLQQLL----AGT--QSLPLYR : 303
HvGA2ox6     : -HPDALIVNVGDLFQAWSNNRYKSVEHKV-VA--N-YKAERFSVAYFLCPSYDSPV-GTC-------------SEPSPYR : 316
HvGA2ox7     : -DPDAFFVNVADALQVLTNGRFSSVKHRV-VV--S-SERPRVSMIFFGGPPMGERL-APLRQLL----GD---GGRSKYR : 315
HvGA2ox8     : -DTDSLFVNVGDSLQVLTNGRFRSVKHRV-VAPTE-GQQPRLSVIYFGGPAPTQRI-APLPELM-------REGEQSLYR : 302
HvGA2ox9     : -IPNALIVNIGDLFQAWSNNRYKSVEHKV-IT--N-ATTERYSLAYFLCPSYDSPI-GAC-------------EEPSPYR : 318
OsGA2ox1     : -DPSAIFVNVGDVLQALTNGRLISIRHRV-IA--T-ACRPRLSTIYFASPPLHARI-SALPETI----TA---SSPRRYR : 343
OsGA2ox10    : -DRKSFFVNVGDVLQVLTNGRFRSVRHRV-MV--S-SPRPRVSVIFFAGPPPRERL-APLPWLV----AED--GGRRRYR : 319
OsGA2ox2     : -DPSAFFVNVGDLLQALTNGRLVSIRHRV-VV--G-TGKPRLSTIYFAAPPLHARI-SALPETV-------AAGAPRRYR : 328
OsGA2ox3     : -DRDSFFVNVGDSLQVLTNGRFKSVKHRV-VA--N-SLKSRVSFIYFGGPPLAQRI-APLPQLL-------GEGEQSLYK : 300
OsGA2ox4     : -DPASFLVIVGDSLQVMTNGRMRSVRHRV-VA--N-KLKSRVSMIYFGGPPLEQRI-APLRQLLVAGVGNGEEEEQSRYE : 318
OsGA2ox5     : -SPSTLIVNVGDLLQAWSNDVYKSVEHRV-MA--N-ATLERFSMAFFLCPSYHTLI-IPSSSHVH--------DDDAHYR : 311
OsGA2ox6     : -RPDALVVNIGDLFQAWSNNRYKSVEHKV-VA--N-AKTDRLSVAYFLCPSYDSLV-GTC-------------GEPSPYR : 324
OsGA2ox7     : GDGDSFFVNVGDTLQVLTNGRFRSVKHRV-VV--N-SEKSRVSMVFFGGPPPGERL-APLPALL----GD---GGRSRYR : 309
OsGA2ox8     : -DPDSFFVNVGDSLQVLTNGRFRSVKHRV-LA--PEGEESRLSVIYFGGPAASQRI-APLEQVM----RE---GEQSLYR : 314
OsGA2ox9     : -IPGALIVNIGDLFQAWSNNRYKSVEHRV-MT--N-ATTERYSVAYFLCPSYDSPI-GTC-------------REPSPYK : 330
TaGA2ox-A1   : -DSSAYFVNVGDLLQALTNGRLVSVRHRV-IA--S-ACRPRLSTIYFAAPPLHARI-SALPEMV-------TADSPPRYR : 335
TaGA2ox-A10  : -DQTSFFVNVGDALQVLTNGRFRSVRHRV-MV--N-SVRPRVSVIFFGGPSPRETL-APLPQLV-------GEGGRSRYR : 325
TaGA2ox-A11  : -RADALIVNVGDLFQAWSNNRYKSVEHKV-VA--N-SKAERFSIAYFMCPLSDSPV-GTY-------------GEPSPYK : 322
TaGA2ox-A3   : -DRDAFFVNVGDSLQVLTNGRFKSVKHRV-VA--N-SLKSRVSMIYFGGPAMTQRI-APLPQLL----GA---GEQSLYK : 300
TaGA2ox-A4   : -NRDAFFVNVGDSLQVLTNGRLRSVRHRV-VA--GNGLKSRVSMIYFGGPPLAQRI-APLPQLL------AGTQSLPLYR : 304
TaGA2ox-A6   : -RPDALIVNIGDLFQAWSNNRYKSVEHKV-VA--N-AKAERLSVAYFLCPSYDSLV-GTC-------------GEPSPYR : 321
TaGA2ox-A7   : -DPDAFFVNVADALQVLTNGRFRSVRHRV-VV--S-SERPRVSMIFFGGPPMGERL-APLRQLL----GD---GGRSRYR : 314
TaGA2ox-A8   : -DPDSLFVNVGDSLQVLTNGRFQSVKHRV-VAPAE-GQQSRLSVIYFGGPAPAQRI-APLPELM-------REGERSLYR : 299
TaGA2ox-A9   : -IPGALIVNIGDLFQAWSNNRYKSVEHKV-VT--N-ATTERYSVAYFLCPSYDSPI-GAC-------------EEPSPYR : 318
TaGA2ox-B1   : -DSSAYFVNVGDLLQALTNGRLVSVRHRV-IT--S-ACRPRLSTIYFAAPPLHARI-SALPEMV-------TAGSPPRYR : 335
TaGA2ox-B10  : -DQTSFFVNVGDALQVLTNGRFRSVRHRV-MV--N-SVRPRVSVIFFGGPAPRETL-APLRQLV-------GEGGRSRYR : 323
TaGA2ox-B11  : -HADALIVNVGDLFQAWSNNRYKSVEHKV-VA--N-SKAERFSVAYFLCPSSDSSV-GTC-------------GEPSPYK : 321
TaGA2ox-B12  : -HAHALMVNVGDLFQAWSNNRYKSVEHRV-VA--N-SKAERFSVAYFMCPPCDSPV-GTC-------------AEPSPYK : 316
TaGA2ox-B13  : -HADALLVNVGDLFQAWSNNIYKSVEHKV-LA--N-SKAERFSIAYFMCPSHDSLI-GTC-------------GGPSPYR : 309
TaGA2ox-B2   : -DPAAFFINVGDLLQALTNGKLVSIRHRV-MA--S-TSKPRLSAIYFAAPALHECI-SALPETV-------TAAAPRRYR : 310
TaGA2ox-B3   : -DRDAFFVNVGDSLQVLTNGRFKSVKHRV-VA--N-SLKSRVSMIYFGGPAMTQRI-APLPQLL-------GAGEQSLYK : 300
TaGA2ox-B4   : -NRDAFFVNVGDSLQVLTNGRLRSVRHRV-VA--GNGLKSRVSMIYFGGPPLAQRI-APLPQLL---------AGTPLYR : 301
TaGA2ox-B6   : -RPDALIVNIGDLFQAWSNNRYKSVEHKV-VA--N-PKAERLSVAYFLCPSYDSPV-STC-------------GEPSAYR : 321
TaGA2ox-B7   : -DADSFFVNVGDALQVLTNGRFRSVRHRV-VV--S-SERPRVSTIFFGGPPFGERL-GPRRQRL-------GDGGRSRYR : 315
TaGA2ox-D1   : -DSSAYFINVGDLLQALTNGRLVSVRHRV-IA--S-ACRPRLSTIYFAAPPLHARI-SALPEMV-------TAGSPPRYR : 335
TaGA2ox-D10  : -DHTSFFVNVGDALQVLTNGRFRSVRHRV-MV--N-SARPRVSVIFFGGPPPRETL-APLPQLV-------GEGGRSRYR : 322
TaGA2ox-D11  : -HADALIVNVGDLFQAWSNNRYKSVEHKV-VA--N-SKAERFSVAYFLCPSSDSSV-GTC-------------GEPSPYK : 322
TaGA2ox-D2   : -DPAAFFINVGDLLQALTNGKLVSIRHRV-MA--S-TSKPRLSAIYFAAPALHESI-SALPETV-------TADAPRRYR : 311
TaGA2ox-D3   : -DRDAFFVNVGDSLQVLTNGRFKSVKHRV-VA--N-SLKSRVSMIYFGGPAMTQRI-APLPQLL-------GAGEQSLYK : 300
TaGA2ox-D4   : -NRDAFFVNVGDSLQVLTNGRLRSVRHRV-VA--GNGLKSRVSMIYFGGPPLAQRI-APLPQLL------AGTQSLPLYR : 304
TaGA2ox-D6   : -RSDALIVNIGDLFQAWSNNRYKSVEHKV-VA--N-AKAERLSVAYFLCPSYDSPV-GTC-------------GEPSPYR : 318
TaGA2ox-D7   : -DPDSFFVNVADALQVLTNGRFRSVRHRV-VV--S-SERPRVSMIFFGGPPFGQRL-APLRQLL----GD---GGRSRYR : 329
TaGA2ox-D8   : -DPDSLFVNVGDSLRVLTNGRFHSVKHRV-VAP-AEGQQSRLSVIYFGGPAPTQRI-APLPELM----RE---GEQSLYR : 300
TaGA2ox-D9   : -IPGALIVNIGDLFQAWSNNRYKSVEHKV-VT--N-ATTERYSVAYFLCPSYDSPI-GAC-------------EEPSPYR : 318
OsGA3ox2     : -VAGAFVVNVGDLFHILTNGRFHSVYHRA-VV--N-RDRDRVSLGYFLGPPPDAEV-APLPEAV-----P-A-GRSPAYR : 326
```

```
                          *        500         *        520         *        540         *
AtGA2ox1    : EFTWSEY---KNSTYNSRLSDNRLQQFERK------------------------------TIKNLLN--------- : 329
AtGA2ox2    : EFTWSQY---KSSAYKSKLGDYRLGLFEKQ----------------------------PLLNHKTLV--------- : 341
AtGA2ox3    : EFTWSQY---KLSAYKTKLGDYRLGLFEKR-----------------------------PPFSLSNV--------- : 335
AtGA2ox4    : TFTWGEY---KKRAYSLRLEDSRLDMFRTCKD-------------------------------------------- : 321
AtGA2ox6    : SFTWGDY---KKATYSLRLDVPRLEFFKTL---------------------------------------------- : 329
AtGA2ox7    : RFSFREYK--EQSEHDVKETGDKVGLSRFLI--------------------------------------------- : 336
AtGA2ox8    : NFSFREFR--QQVQEDVKKFGFKVGLPRFL---------------------------------NHVY--------- : 338
BdGA2ox1    : SFTWAEY---KTTMYSLRLSHSRLDLFCVH------------------------DDDDNSSDVSKEK--------- : 380
BdGA2ox10   : EFTWREY---KASAYRTKLAENRLCHFETTS--------------------------------------------- : 349
BdGA2ox2    : PFTWAEY---KKNMYTLSLSHNRLELYNAV----------------------NADAGEARPRSGIAS--------- : 343
BdGA2ox3    : EFTWSEY---KKAAYKSRLGDNRLAQFQK----------------------------------------------- : 326
BdGA2ox4    : EFTWGEY---KKAAYRSRLGDNRLAPFQMT-------------------LVELQHAHADAAGLHHRS--------- : 354
BdGA2ox5    : NFTFGEYR--SQVREDVRLTGRKVGLPRFR----------------------RHVSPSPASLTHQLS--------- : 383
BdGA2ox6    : SFTFGEYR--RKVQDDVKRTGKKIGLPNFL---------------------------KHPPIVDGLK--------- : 358
BdGA2ox7    : EFTWKEY---KSTGCRGRLAEDRLCLFEN----------------------------------------------- : 358
BdGA2ox8a   : DFTWGEY---KAAAYKTRLGDHRLGPFQVQ----------------------------LLPPAAAKA--------- : 360
BdGA2ox8b   : DFTWGEY---KAAAYKTRLGDNRLGPYELRNVADTNPTALITASKEPTAADHCCSNSSSSSAACVVVQPPHVAQVH : 383
BdGA2ox9    : TFTFGEYR--RRVQEDVKKTGKKVGLPGFLL--------------------------------------------- : 356
HvGA2ox1    : SFTWAEY---KTAMYSLRLSHSRLDLF--H------------------------VDDDESGNGSKGK--------- : 375
HvGA2ox10   : EFTWCEY---KASAYGTKLAANRLCHFETTN--------------------------------------------- : 349
HvGA2ox2    : PFTWAEY---KKTMYTLRLSHNRLDLFAVV---------------------------GDSGEDKPRI--------- : 350
HvGA2ox3    : DFTWGEY---KKAAYNSRLGDNRLAHFHR----------------------------------------------- : 326
HvGA2ox4    : DFTWGEY---KKAAYRSRLGDNRLAPFETP-------------------------LVAMPHAAHHRS--------- : 342
HvGA2ox6    : PFTFGEYR--RKVQDDVKRTGKKIGLPNFL---------------------------KHSPVHGLSS--------- : 354
HvGA2ox7    : EFTWKEY---KSSTHKGRLATDRLCSFEN----------------------------------------------- : 341
HvGA2ox8    : DFTWAEY---KKAAYKSRLGDHRLGPFELP-----------AAATAQESTTKADHYCSSNAAVQAPAAAPHVARVH : 364
HvGA2ox9    : TFTFGEYR--RRVQEDVKKTGKKIGLPNFLL--------------------------------------------- : 347
OsGA2ox1    : SFTWAEY---KTTMYSLRLSHSRLELFKID-------------------------DDDSDNASEGKA--------- : 382
OsGA2ox10   : EFTWREY---KASAYRTKLAENRLCHFETEAD-------------------------------------------- : 348
OsGA2ox2    : AFTWAEY---KRTMYTLRLSHNRLDLFHAG----------------------DGDGDAGVGDDDDHE--------- : 370
OsGA2ox3    : EFTWDEY---KKAAYKSRLGDNRLAQFEKK---------------------------------------------- : 327
OsGA2ox4    : EFTWGEY---KKAAYLSRLSDNRLAPFHRQ----------------------------PPPVANPLA--------- : 354
OsGA2ox5    : SFTFGEYR--KQIMEDVRSTGRKIGLHRFRTR-------------------------------------------- : 341
OsGA2ox6    : AFTFGEYR--KKVQEDVRTTGKKIGLPNFF-------------------------------KHSSVQ--------- : 358
OsGA2ox7    : EFTWKEY---KGSGCKGRLADDRLCRFEN----------------------------------------------- : 335
OsGA2ox8    : EFTWGEY---KKAAYKTRLGDNRLGPYELQ-------------------------HAAANDEAATKK--------- : 353
OsGA2ox9    : AFTFGEYR--RRVQEDVKKTGKKTGLSNFLV--------------------------------------------- : 359
TaGA2ox-A1  : SFTWAEY---KTAMYSLRLSHSRLELFHVD--------------------------DDESGNGSKGK--------- : 373
TaGA2ox-A10 : EFTWREY---KASAYRTKLAANRLCHFETTS--------------------------------------------- : 353
TaGA2ox-A11 : PFTFEEYR--RSVRDDVERTGKKIGLPNFL--------------------KRSTVHGLDDDIMDLSS--------- : 367
TaGA2ox-A3  : DFTWGEY---KKAAYNSRLGDNRLAHFHR----------------------------------------------- : 326
TaGA2ox-A4  : DFTWGEY---KKAAYRSRLGDNRLAPFEAP-------------------------PVATPHADRHRS--------- : 343
TaGA2ox-A6  : PFTFGEYR--RKVQDDVKRTGKKIGLPNFL-----------------------KQSPVDAMNHSLCT--------- : 363
TaGA2ox-A7  : DFTWKEY---KSSTHKGRLAADRLCSFEN----------------------------------------------- : 340
TaGA2ox-A8  : DFTWAEY---KKAAYKSRLGDHRLGPFELP---------------AAKETNSSDHHCSSNAVQPPARAPPHVATVY : 357
TaGA2ox-A9  : TFTFGEYR--RRVQEDVKKMGKKIGLPNFLL--------------------------------------------- : 347
TaGA2ox-B1  : SFTWAEY---KTAMYSLRLSHSRLDLFHVD--------------------------DDESGNGSKGK--------- : 373
TaGA2ox-B10 : EFTWREY---KASAYRTKLAANRLGHFETTS--------------------------------------------- : 351
TaGA2ox-B11 : PFTFGEYR--RSVQDDVKRTGKKIGLPNFL--------------------KRSTVHGLDDDTMDLSS--------- : 366
TaGA2ox-B12 : PFTFGEYR--RKVQEDVTRTGKKIGLSNFL---------------------------KGSTFDGLAE--------- : 354
TaGA2ox-B13 : PFTFEEYR--RKVQDDVRRTGKKIGLPNFL-------------------------------KRSTIS--------- : 343
TaGA2ox-B2  : PFTWAEY---KKTMYTLRLSHNRLDLFKVI---------------------LVGDVGEGAGEDRSRI--------- : 353
TaGA2ox-B3  : DFTWGEY---KKAAYNSRLGDNRLAQFHR----------------------------------------------- : 326
TaGA2ox-B4  : EFTWGEY---KKAAYRSRLGDNRLAPFETP-------------------------LVAMAHADHHRS--------- : 340
TaGA2ox-B6  : PFTFGEYR--RKVQDDVKRTGKKIGLPNFL-----------------------KQSPVDGMNHTLCS--------- : 363
TaGA2ox-B7  : EFTWKEY---KSSTHKGRLATDRLCSFEN----------------------------------------------- : 341
TaGA2ox-D1  : SFTWAEY---KTAMYSLRLSHSRLDLFHVD--------------------------DDESGNGSKGK--------- : 373
TaGA2ox-D10 : EFTWREY---KASAYRTKLAANRLCHFETTS--------------------------------------------- : 350
TaGA2ox-D11 : PFTFGEYR--RSVHDDVERTGKKIGLPNFL--------------------KRSTVHGLDDDTMDLSS--------- : 367
TaGA2ox-D2  : PFTWAEY---KKTMYTLRLSHNRLDLFKVV-----------------------GEGGGDSGEDKSRI--------- : 352
TaGA2ox-D3  : DFTWGEY---KKAAYNSRLGDNRLAQFHR----------------------------------------------- : 326
TaGA2ox-D4  : DFTWGEY---KKAAYRSRLGDNRLAPFEAP-------------------------PVATPDADRHRS--------- : 343
TaGA2ox-D6  : PFTFGEYR--RKVQDDVKRTGKKIGLPNFL-----------------------KQSPVDAMNHGLCF--------- : 360
TaGA2ox-D7  : EFTWKEY---KSSTHKGRLATDRLCSFEN----------------------------------------------- : 355
TaGA2ox-D8  : DFTWAEY---KKAAYKSRLGDNRLGPFELF-----------PSAAQDCSSNAVQPPAPAPPHVAPVH--------- : 353
TaGA2ox-D9  : TFTFGEYR--RRVQEDVKKTGKKIGLPNFLL--------------------------------------------- : 347
OsGA3ox2    : AVTWPEYMAVRKKAFATGGSALKMVSTDAA-----------------AAADEHDDVAAAADVHA------------ : 373
```

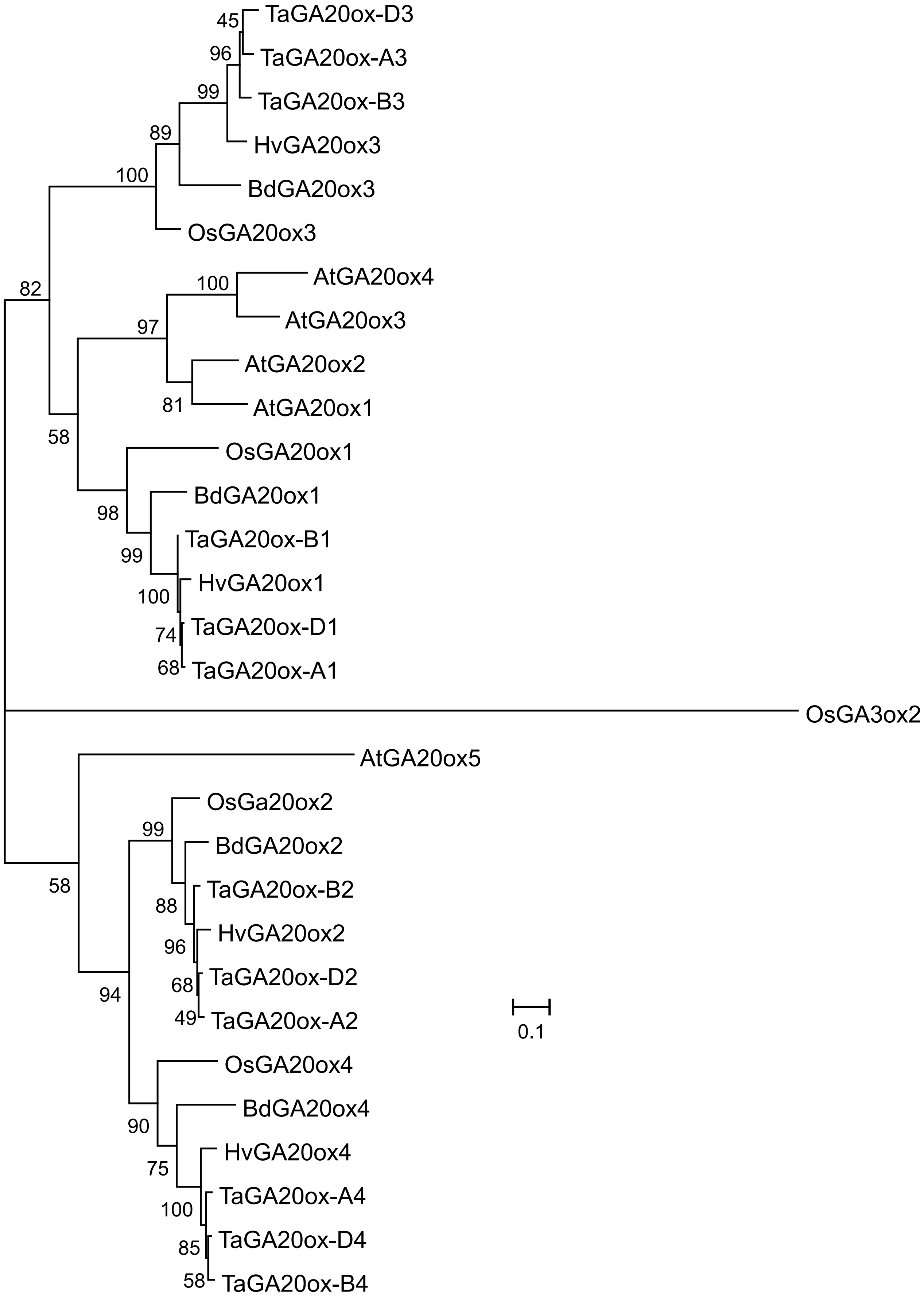

Figure S11. PHYML tree of GA20ox proteins sequences from Arabidopsis, rice, Brachypodium, wheat and barley using OsGA3ox2 as outgroup. Numbers show bootstrap support; the scale bar shows substitutions per site. Non-aligned residues were trimmed from the alignment prior to tree generation using PhyML within TOPALi as reported in Materials and Methods.

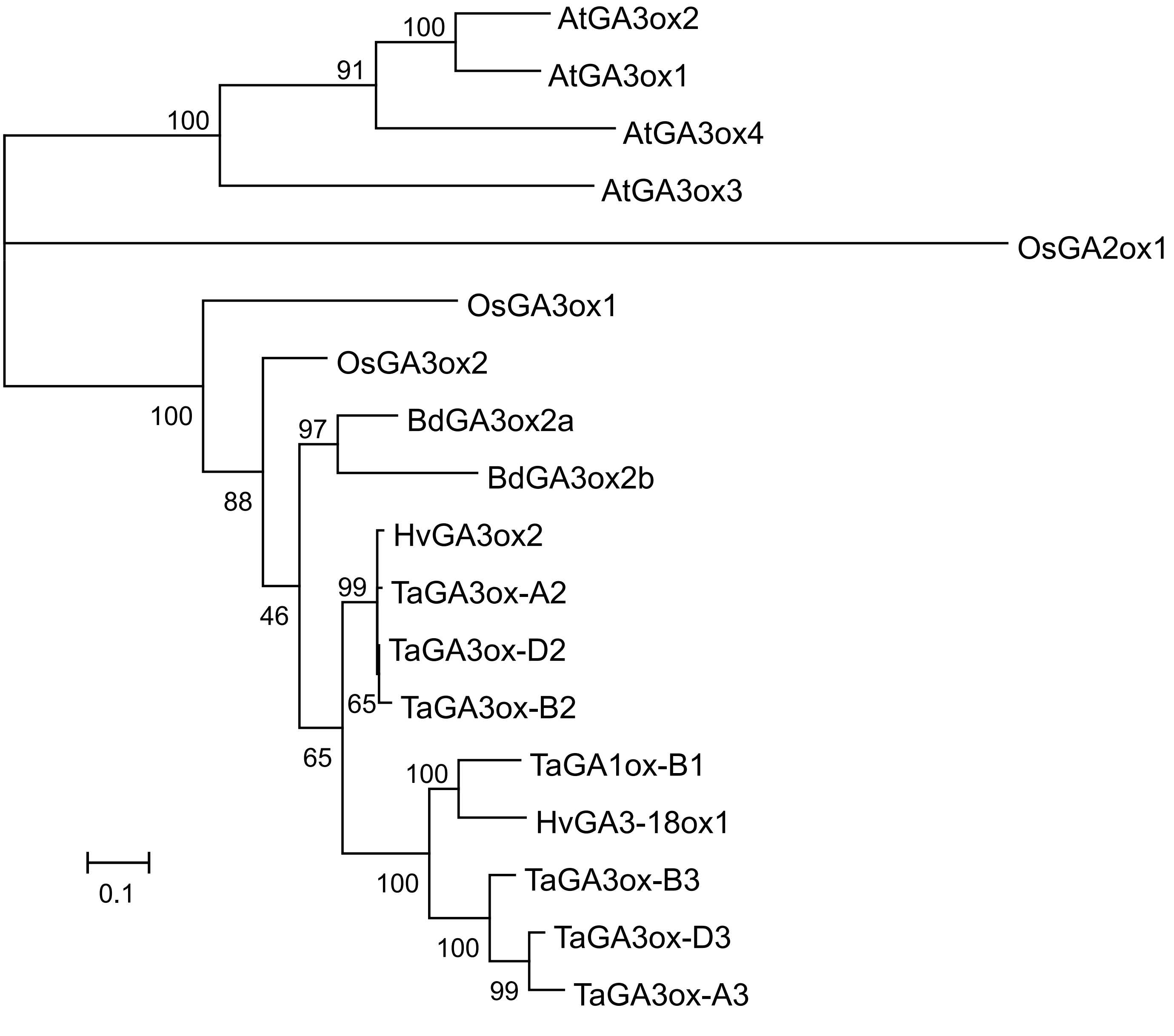

Figure S12. PHYML tree of GA3ox proteins sequences from Arabidopsis, rice, Brachypodium, wheat and barley using OsGA2ox1 as outgroup. Numbers show bootstrap support; the scale bar shows substitutions per site. Non-aligned residues were trimmed from the alignment prior to tree generation using PhyML within TOPALi as reported in Materials and Methods.

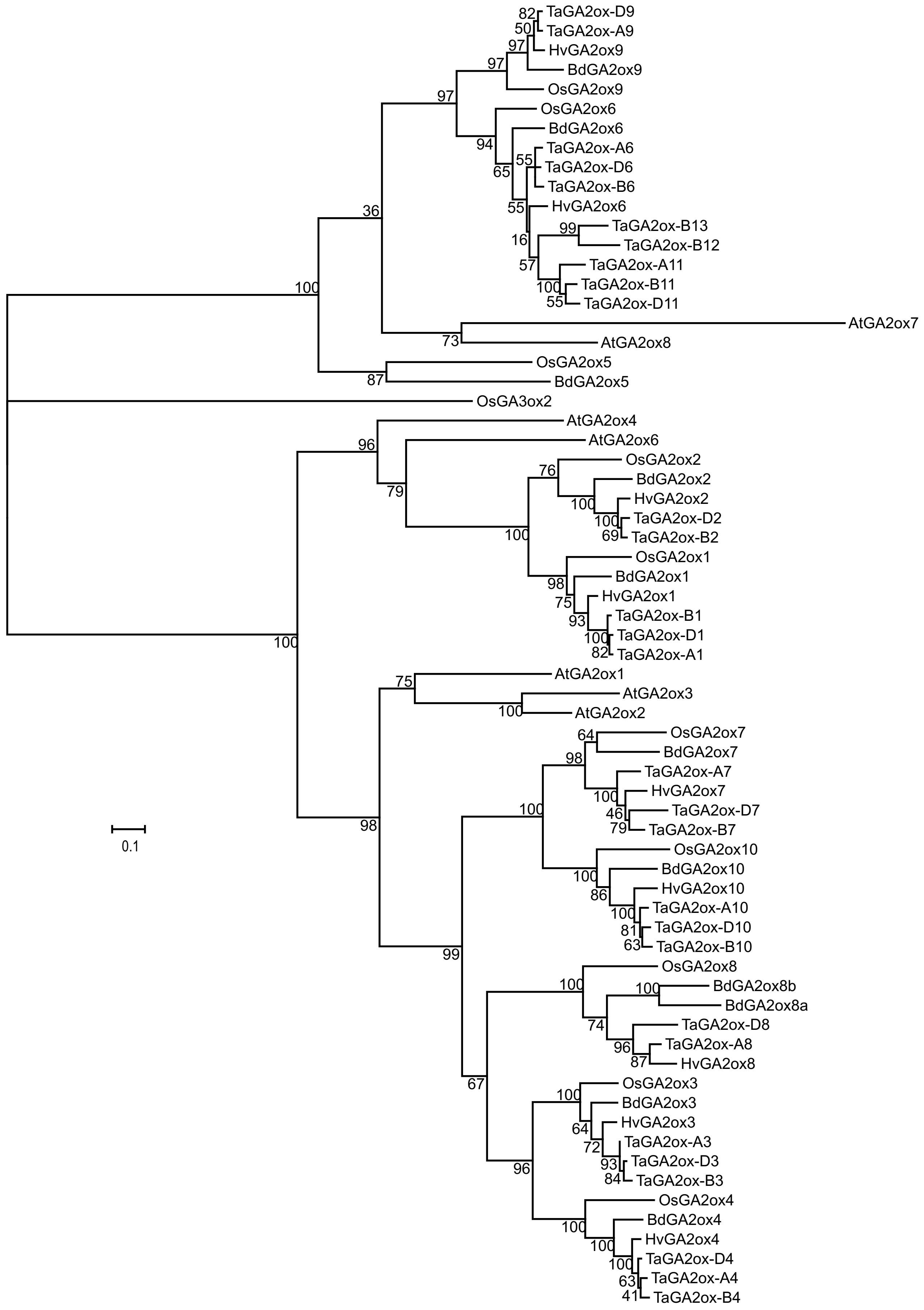

Figure S13. PHYML tree of GA2ox proteins sequences from Arabidopsis, rice, *Brachypodium,* wheat and barley using OsGA3ox2 as outgroup. Numbers show bootstrap support; the scale bar shows substitutions per site. Non-aligned residues were trimmed from the alignment prior to tree generation using PhyML within TOPALi as reported in Materials and Methods.
