# Supplementary material for: Heterologous expression and transcript analysis of gibberellin biosynthetic genes of grasses reveals novel functionality in the GA3ox family
Source: BMC Plant Biol. 2015 Jun 5;15:130. doi: 10.1186/s12870-015-0520-7 (PMC4455330; doi:10.1186/s12870-015-0520-7)
Supplement: Supplementary file 2 — Rice, Brachypodium, barley and wheat genes encoding GA 2-ODDs including accession numbers. [file 12870_2015_520_MOESM2_ESM.pdf]

Additional file 2: Cereal GA 2-ODD genes and accession numbers

Target	Rice gene	Brachypodium gene	T. aestivum survey sequence (Ensemble plants v.25)			T. durum transcript		T. urartu transcript	T. urartu genomic contig	Ae. tauschii genomic contig	Barley cds
			A	B	D	A	B	A	A	D	H
**GA20ox1**	Os03g63970	Bradi1g00950	FL (4AL)<br>Traes_4AL_FABDF4EDA	FL[7] (5BL)<br>Traes_5BL_D412D28CC	FL (5DL)<br>Traes_5DL_3E77D28A6	FL<br>UCW_Tt-k61_contig_4086	FL<br>UCW_Tt-k31_contig_24761	FL<br>UCW_Tu-k31_contig_19469	FL<br>AOTI011205483	Partial<br>AOCO010361971	FL (5H)<br>MLOC_16059.1
**GA20ox2**	Os01g66100	Bradi2g57030	-	FL (3B) TRAES3BF021500010CFD_t1	FL[1] (3D)<br>EMBL:LN828667	-	FL<br>UCW_Tt-k51_contig_72276	Partial<br>UCW_Tu-k31_contig_43195	Partial<br>AOTI010822325	FL<br>AOCO010414640	FL (3H)<br>MLOC_56462.1
**GA20ox3**	Os07g07420	Bradi1g56200	-	FL (3B)<br>TRAES3BF078000020CFD_t1	FL[1] (3D)<br>EMBL:LN828668	FL<br>UCW_Tt-k31_contig_77943	FL<br>UCW_Tt-k31_contig_77942	-	Partial<br>AOTI010920090	Partial<br>AOCO010472349	FL (3H)<br>MLOC_66389.1
**GA20ox4**	Os05g34854	Bradi2g24980	FL[1] (1AL)<br>EMBL:LN828665	FL[1,2] (1BL)<br>EMBL:LN828666	FL[1,2] (1DL)<br>EMBL:LN828669	FL<br>UCW_Tt-k45_contig_52969	-	Partial<br>UCW_Tu-k45_contig_45615	Partial<br>AOTI010446710	Partial<br>AOCO010054278	FL (1H)<br>AK373555
**GA3ox1**	Os05g08540	-	-	-	-	-	-	-	-	-	-
**GA3ox2**	Os01g08220	Bradi2g04840(a)<br>Bradi4g23570(b)	Partial[1,3] (3A)<br>Traes_3AS_3A79F81AF	FL (3B)<br>TRAES3BF085400020CFD_t1	FL[1] (3D)<br>EMBL:LN828690	FL<br>UCW_Tt-k25_contig_19860	-	FL<br>UCW_Tu-k25_contig_19670	Partial<br>AOTI010990317	Partial<br>AOCO010773132	FL (3H)<br>AB189152
**GA3ox3**	-	-	FL[1] (2AL)<br>EMBL:LN828688	FL[1] (2BL)<br>EMBL:LN828689	Partial (2DL)<br>Traes_2DL_66F9CEA3C	FL<br>UCW_Tt-k35_contig_80532	Partial<br>UCW_Tt-k55_contig_83042	-	Partial<br>AOTI010197447	-	-
**GA1ox1**	-	-	-	FL (2BL)<br>Traes_2BL_9E115B19F	-	-	FL<br>UCW_Tt-k35_contig_80660		-	-	FL (2H) HvGA3,18ox1<br>MLOC_128
**GA2ox1**	Os05g06670	Bradi2g34840	FL[1] (1AS)<br>EMBL:LN828670	FL (1BS)<br>Traes_1BS_0EDD50331.1	FL (1DS)<br>Traes_1DS_125A395C1	Partial<br>UCW_Tt-k21_contig_42061	-	Partial<br>UCW_Tu-k21_contig_19294	Partial<br>AOTI011122791	Partial<br>AOCO010626498	FL[6] (1H)<br>-
**GA2ox2**	Os01g22910	Bradi2g12440	-	FL[1] (7BL)<br>EMBL:LN828677	FL[1] (7D)<br>EMBL:LN828681	-	-	-	-	Partial<br>AOCO010286040	FL[6] (7H)<br>-
**GA2ox3**	Os01g55240	Bradi2g50280	Partial[5] (3AL)<br>Traes_3AL_14A36F545	FL (3B)<br>TRAES3BF090300180CFD_t1	FL[1] (3D)<br>EMBL:LN828682	FL<br>UCW_Tt-k51_contig_27371	-	FL<br>UCW_Tu-k35_contig_25670	Partial<br>AOTI010091872	Partial<br>AOCO010496281	FL (3H)<br>AK364775
**GA2ox4**	Os05g43880	Bradi2g19900	Partial (1AL)<br>Traes_1AL_5ED72B143	FL[1] (1B)<br>EMBL:LN828678	FL[1] (5BL)<br>EMBL:LN828683	Partial<br>UCW_Tt-k41_contig_81164	FL<br>UCW_Tt-k31_contig_24170	FL<br>UCW_Tu-k35_contig_45252	FL<br>AOTI011226444	Partial<br>AOCO010507273	Partial (1H)<br>MLOC_13981.1
**GA2ox5**	Os07g01340	Bradi1g59570	-	-	-	-	-	-	-	-	-
**GA2ox6**	Os04g44150	Bradi5g16040	FL[1] (2AL)<br>EMBL:LN828673	FL (2BL)<br>Traes_2BL_1B1358201	FL[1] (2DL)<br>EMBL:LN828684	FL<br>UCW_Tt-k31_contig_60185	FL<br>UCW_Tt-k31_contig_60185	Partial<br>UCW_Tu-k31_contig_28625	Partial<br>AOTI010554200	Partial<br>AOCO010171523	FL (2H)<br>MLOC_1557.1
**GA2ox7**	Os01g11150	Bradi2g06670	-	FL (3B)<br>TRAES3BF118400050CFD_t1	FL[1] (3DS)<br>EMBL:LN828685	Partial<br>UCW_Tt-k25_contig_44496	FL<br>UCW_Tt-k51_contig_19754	Partial<br>UCW_Tu-k21_contig_25357	Partial<br>AOTI010095549	Partial<br>AOCO010565863	FL (3H)<br>AK373885
**GA2ox8**	Os05g48700	Bradi2g16730(a)<br>Bradi2g16750(b)	FL[1] (1AL)<br>EMBL:LN828674	-	FL[1,2] (5BL)<br>EMBL:LN828686	Partial<br>UCW_Tt-k31_contig_41182	Partial<br>UCW_Tt-k41_contig_42235	Partial<br>UCW_Tu-k31_contig_39145	Partial	Partial<br>AOCO010629416	FL[6] (1H)<br>-
**GA2ox9**	Os02g41954	Bradi3g49390	FL (6AL)<br>Traes_6AL_6926D08B4	Partial (6BL)<br>Traes_6BL_B8497D305	FL[1,2] (6DL)<br>EMBL:LN828687	FL<br>UCW_Tt-k21_contig_25899	-	FL<br>UCW_Tu-k55_contig_21546	FL<br>AOTI010464169	Partial	FL (6H)<br>MLOC_6996.1
**GA2ox10**	Os05g11810	Bradi2g32580	FL[1] (1AS)<br>EMBL:LN828671	FL[1] (1BS)<br>Traes_1BS_2C29ED3EF	FL (1DS)<br>EMBL:LN828679	-	FL<br>UCW_Tt-k25_contig_50873	FL<br>UCW_Tu-k45_contig_25977	Partial	Partial	FL (1H)<br>AK357218
**GA2ox11**	-	-	FL[1] (4AS)<br>EMBL:LN828672	FL[1] (4BL)<br>EMBL:LN828675	FL[1] (4DL)<br>EMBL:LN828680	-	-	-	FL<br>AOTI010991515	FL<br>AOCO010008031	-
**GA2ox12**	-	-	-	FL (4BL)<br>Traes_4BL_57623F302	-	-	-	-	FL? (repeated section)<br>AOTI010699551	-	-
**GA2ox13**	-	-	-	FL[1] (4BL)<br>EMBL:LN828676	-	-	-	-	-	Pseudogene<br>AOCO010487378	-

Notes

FL: Full length genomic or CDS sequence

IWGSC cDNA sequences (Traes…) are available from Ensemble Plants (http://plants.ensembl.org/Triticum_aestivum/Info/Index)

Transcript sequences from T. durum cv. Kronos are available at http://maswheat.ucdavis.edu/Transcriptome/index.htm

Transcript sequences from T. urartu are available at NCBI (http://www.ncbi.nlm.nih.gov/bioproject/191053)

Barley cds sequences are available at http://pgsb.helmholtz-muenchen.de/plant/barley/index.jsp

[1]Reassembly of IWGSC chromosome arm reads

[2]Missing data in intron

[3]FL cDNA from cv. Maris Huntsman

[4]7bp insertion in exon 2

[5]FL cDNA from cv. Avalon

[6]CDS absent from barley high-confidence gene list

[7]Intron sequence (2 bp) in IWGSC transcript
