# Supplementary material for: Heterologous expression and transcript analysis of gibberellin biosynthetic genes of grasses reveals novel functionality in the GA3ox family
Source: BMC Plant Biol. 2015 Jun 5;15:130. doi: 10.1186/s12870-015-0520-7 (PMC4455330; doi:10.1186/s12870-015-0520-7)
Supplement: Supplementary file 3 — Homoeolog-specific expression of GA biosynthetic and signalling genes across five tissues of wheat at three developmental stages. [file 12870_2015_520_MOESM3_ESM.pdf]

Additional file 3 - Homoeologue-specific GA gene transcript levels in wheat tissues. Means of 2 reps +/- standard error; Z=Zadoks stage.

Gene	root_Z10	root_Z13	root_Z39	leaf_Z10	leaf_Z23	leaf_Z71	stem_Z30	stem_Z32	stem_Z65	spike_Z32	spike_Z39	spike_Z65	grain_Z71	grain_Z75	grain_Z85
**TaCPS-A1**	0.04 ± 0.03	0.21 ± 0.07	0.11 ± 0.06	0.28 ± 0.01	0.76 ± 0.14	0.15 ± 0.01	3.15 ± 0.01	1.28 ± 0.01	1.40 ± 0.01	0.93 ± 0.30	2.73 ± 0.33	10.06 ± 0.92	0.34 ± 0.06	4.15 ± 0.26	2.97 ± 0.02
**TaCPS-B1**	0.82 ± 0.09	1.33 ± 0.43	1.33 ± 0.39	0.66 ± 0.11	1.42 ± 0.07	1.18 ± 0.13	3.62 ± 0.04	1.88 ± 0.24	2.17 ± 0.03	2.23 ± 0.15	4.67 ± 1.05	7.05 ± 0.04	1.42 ± 0.33	0.45 ± 0.09	7.27 ± 0.11
**TaCPS-D1**	0.30 ± 0.08	0.82 ± 0.39	0.87 ± 0.47	0.68 ± 0.03	1.37 ± 0.08	1.61 ± 0.12	5.49 ± 0.11	1.68 ± 0.16	1.45 ± 0.06	3.08 ± 0.17	4.71 ± 0.62	8.17 ± 0.34	1.01 ± 0.15	0.46 ± 0.08	2.41 ± 0.08
**TaKS-B1**	0.62 ± 0.06	1.46 ± 0.38	1.71 ± 0.59	0.29 ± 0.03	0.64 ± 0.11	0.52 ± 0.02	1.05 ± 0.10	1.30 ± 0.04	0.87 ± 0.04	1.27 ± 0.35	1.71 ± 0.09	2.86 ± 0.19	1.31 ± 0.21	2.61 ± 0.24	0.22 ± 0.03
**TaKS-D1**	0.94 ± 0.00	1.66 ± 0.69	1.39 ± 0.63	0.47 ± 0.03	1.78 ± 0.19	2.42 ± 0.16	1.52 ± 0.06	1.99 ± 0.03	2.60 ± 0.03	2.63 ± 0.57	4.36 ± 0.21	5.74 ± 0.36	1.52 ± 0.16	1.63 ± 0.05	0.89 ± 0.06
**TaKO-A1**	1.47 ± 0.28	2.68 ± 0.19	2.39 ± 0.07	1.72 ± 0.31	1.19 ± 0.26	1.29 ± 0.08	1.83 ± 0.12	2.09 ± 0.24	3.03 ± 0.11	2.50 ± 0.38	1.86 ± 0.20	8.85 ± 1.41	3.74 ± 0.10	11.12 ± 0.72	9.33 ± 0.06
**TaKO-B2**	1.05 ± 0.05	1.92 ± 0.42	1.34 ± 0.12	1.20 ± 0.04	0.75 ± 0.13	0.00 ± 0.00	2.02 ± 0.10	1.89 ± 0.16	1.00 ± 0.11	2.19 ± 0.36	1.50 ± 0.22	4.42 ± 0.40	1.79 ± 0.09	5.19 ± 0.64	16.74 ± 1.99
**TaKO-D1**	1.09 ± 0.08	2.28 ± 0.70	1.72 ± 0.72	1.23 ± 0.01	0.38 ± 0.03	2.34 ± 0.07	0.90 ± 0.06	1.94 ± 0.02	1.67 ± 0.18	1.39 ± 0.11	4.03 ± 0.44	6.53 ± 0.81	2.07 ± 0.24	7.19 ± 0.85	76.80 ± 2.02
**TaKAO-A1**	0.78 ± 0.20	2.30 ± 1.29	1.48 ± 0.07	2.57 ± 0.29	1.87 ± 0.30	0.52 ± 0.08	0.98 ± 0.31	2.58 ± 0.01	4.33 ± 0.17	3.65 ± 0.26	1.43 ± 0.05	3.18 ± 0.44	0.92 ± 0.05	15.14 ± 0.44	3.68 ± 0.29
**TaKAO-D1**	1.67 ± 0.25	1.86 ± 0.18	1.98 ± 1.11	0.94 ± 0.23	0.72 ± 0.07	0.13 ± 0.00	0.20 ± 0.01	0.57 ± 0.04	6.08 ± 0.51	0.79 ± 0.09	0.28 ± 0.05	1.71 ± 0.11	0.31 ± 0.08	3.82 ± 0.09	0.25 ± 0.01
**TaKAO-A2**	0.66 ± 0.00	0.29 ± 0.27	0.21 ± 0.11	0.28 ± 0.03	0.24 ± 0.01	0.02 ± 0.02	0.58 ± 0.07	1.24 ± 0.26	1.06 ± 0.13	1.05 ± 0.10	0.49 ± 0.00	2.26 ± 0.14	0.60 ± 0.05	7.18 ± 0.36	0.89 ± 0.14
**TaGA13ox1A**	1.41 ± 0.04	1.41 ± 0.33	2.15 ± 0.43	2.49 ± 0.36	4.70 ± 0.40	32.58 ± 1.05	6.58 ± 0.71	16.32 ± 0.91	22.82 ± 0.08	0.61 ± 0.05	0.60 ± 0.13	4.12 ± 0.63	6.55 ± 0.53	3.12 ± 0.07	0.28 ± 0.01
**TaGA13ox1B**	4.49 ± 0.06	7.33 ± 3.76	7.62 ± 3.56	1.99 ± 0.35	1.93 ± 0.42	17.61 ± 0.01	1.97 ± 0.07	6.18 ± 0.63	7.51 ± 0.28	0.29 ± 0.10	0.42 ± 0.25	2.63 ± 0.44	2.15 ± 0.21	0.82 ± 0.23	2.65 ± 0.18
**TaGA13ox1D**	4.21 ± 0.28	3.96 ± 1.33	3.67 ± 0.93	0.78 ± 0.03	2.55 ± 0.08	14.58 ± 0.51	1.51 ± 0.17	6.42 ± 0.91	4.85 ± 0.07	0.37 ± 0.16	0.39 ± 0.17	2.66 ± 0.74	7.45 ± 0.18	3.30 ± 0.07	1.95 ± 0.12
**TaGA13ox2A**	0.05 ± 0.05	1.31 ± 0.92	0.68 ± 0.09	0.00 ± 0.00	0.00 ± 0.00	2.10 ± 0.12	0.03 ± 0.01	0.03 ± 0.01	0.22 ± 0.04	0.06 ± 0.02	1.92 ± 0.13	7.40 ± 0.39	2.03 ± 0.46	0.22 ± 0.08	0.09 ± 0.01
**TaGA13ox2B**	0.21 ± 0.06	1.77 ± 1.33	0.73 ± 0.06	0.00 ± 0.00	0.00 ± 0.00	1.14 ± 0.04	0.06 ± 0.01	0.06 ± 0.02	0.24 ± 0.03	0.41 ± 0.06	1.40 ± 0.11	9.64 ± 0.37	2.03 ± 0.53	0.40 ± 0.03	0.60 ± 0.05
**TaGA13ox2D**	0.03 ± 0.01	0.16 ± 0.11	0.13 ± 0.04	0.02 ± 0.02	0.00 ± 0.00	0.14 ± 0.00	0.04 ± 0.02	0.01 ± 0.01	0.18 ± 0.04	0.04 ± 0.00	0.40 ± 0.06	3.56 ± 0.40	0.64 ± 0.01	1.04 ± 0.14	0.54 ± 0.04
**TaGA20ox-A1**	0.14 ± 0.11	0.28 ± 0.02	0.39 ± 0.06	0.86 ± 0.08	0.64 ± 0.00	0.61 ± 0.02	0.54 ± 0.14	1.19 ± 0.04	0.58 ± 0.03	0.43 ± 0.13	0.67 ± 0.01	1.66 ± 0.10	0.20 ± 0.04	0.08 ± 0.03	0.16 ± 0.01
**TaGA20ox-B1**	0.67 ± 0.03	0.54 ± 0.17	0.41 ± 0.23	0.96 ± 0.18	0.86 ± 0.16	0.32 ± 0.08	0.44 ± 0.18	1.43 ± 0.29	0.65 ± 0.03	0.51 ± 0.11	0.52 ± 0.09	1.14 ± 0.38	0.51 ± 0.12	0.22 ± 0.05	0.00 ± 0.00
**TaGA20ox-D1**	0.13 ± 0.02	0.85 ± 0.45	0.59 ± 0.05	1.04 ± 0.22	0.77 ± 0.02	0.12 ± 0.01	1.00 ± 0.43	1.45 ± 0.07	0.25 ± 0.04	0.49 ± 0.23	0.79 ± 0.02	0.66 ± 0.01	0.04 ± 0.00	0.40 ± 0.04	0.06 ± 0.06
**TaGA20ox-A2**	0.07 ± 0.05	0.05 ± 0.05	0.09 ± 0.09	0.16 ± 0.07	0.71 ± 0.13	0.61 ± 0.16	0.35 ± 0.11	0.12 ± 0.06	0.55 ± 0.08	0.55 ± 0.21	0.54 ± 0.12	0.25 ± 0.06	0.10 ± 0.01	0.15 ± 0.05	0.04 ± 0.00
**TaGA20ox-B2**	0.01 ± 0.01	0.00 ± 0.00	0.00 ± 0.00	0.29 ± 0.14	1.26 ± 0.06	0.95 ± 0.17	0.48 ± 0.25	0.08 ± 0.06	1.70 ± 0.32	0.24 ± 0.01	0.73 ± 0.17	0.24 ± 0.05	0.18 ± 0.03	0.02 ± 0.02	0.15 ± 0.11
**TaGA20ox-D2**	0.03 ± 0.03	0.05 ± 0.02	0.13 ± 0.13	0.11 ± 0.02	1.28 ± 0.32	5.42 ± 0.23	0.40 ± 0.09	0.22 ± 0.11	0.77 ± 0.30	0.67 ± 0.02	0.90 ± 0.21	1.08 ± 0.18	0.43 ± 0.16	0.00 ± 0.00	0.02 ± 0.02
**TaGA20ox-A3**	0.02 ± 0.02	0.00 ± 0.00	0.00 ± 0.00	0.00 ± 0.00	0.00 ± 0.00	0.00 ± 0.00	0.00 ± 0.00	0.00 ± 0.00	0.00 ± 0.00	0.00 ± 0.00	0.00 ± 0.00	0.29 ± 0.06	0.12 ± 0.08	9.33 ± 0.06	0.03 ± 0.03
**TaGA20ox-B3**	0.00 ± 0.00	0.02 ± 0.02	0.00 ± 0.00	0.02 ± 0.02	0.00 ± 0.00	0.00 ± 0.00	0.00 ± 0.00	0.00 ± 0.00	0.00 ± 0.00	0.00 ± 0.00	0.02 ± 0.02	0.39 ± 0.13	0.10 ± 0.06	26.09 ± 0.09	0.12 ± 0.03
**TaGA20ox-D3**	0.05 ± 0.01	0.20 ± 0.16	0.28 ± 0.16	0.00 ± 0.00	0.00 ± 0.00	0.00 ± 0.00	0.02 ± 0.02	0.00 ± 0.00	0.00 ± 0.00	0.00 ± 0.00	0.00 ± 0.00	0.09 ± 0.05	0.10 ± 0.06	0.71 ± 0.10	0.02 ± 0.02
**TaGA20ox-A4**	0.14 ± 0.04	0.07 ± 0.07	0.00 ± 0.00	0.25 ± 0.11	0.11 ± 0.02	0.19 ± 0.07	0.00 ± 0.00	0.14 ± 0.03	0.00 ± 0.00	0.34 ± 0.09	0.54 ± 0.05	6.35 ± 0.39	0.11 ± 0.01	0.35 ± 0.04	0.85 ± 0.13
**TaGA20ox-B4**	0.03 ± 0.03	0.00 ± 0.00	0.00 ± 0.00	0.01 ± 0.01	0.00 ± 0.00	1.05 ± 0.06	0.00 ± 0.00	0.00 ± 0.00	0.12 ± 0.02	0.35 ± 0.08	0.41 ± 0.12	1.55 ± 0.16	0.11 ± 0.01	0.20 ± 0.00	0.09 ± 0.05
**TaGA20ox-D4**	0.59 ± 0.15	0.05 ± 0.05	0.09 ± 0.09	0.19 ± 0.04	0.09 ± 0.04	0.12 ± 0.08	0.00 ± 0.00	0.00 ± 0.00	0.28 ± 0.05	0.28 ± 0.12	0.42 ± 0.12	1.86 ± 0.56	0.42 ± 0.12	0.43 ± 0.07	0.34 ± 0.03
**TaGA3ox-A2**	0.69 ± 0.12	1.41 ± 0.37	1.28 ± 0.85	0.29 ± 0.10	0.06 ± 0.03	0.00 ± 0.00	1.03 ± 0.63	0.61 ± 0.02	0.07 ± 0.00	0.33 ± 0.04	0.84 ± 0.05	3.40 ± 0.92	0.04 ± 0.00	0.11 ± 0.00	0.38 ± 0.10
**TaGA3ox-B2**	0.21 ± 0.02	0.51 ± 0.25	0.39 ± 0.17	0.63 ± 0.10	0.14 ± 0.04	0.00 ± 0.00	4.69 ± 1.48	3.02 ± 0.49	0.07 ± 0.07	0.95 ± 0.13	1.12 ± 0.22	3.41 ± 0.68	0.41 ± 0.01	0.00 ± 0.00	0.13 ± 0.03
**TaGA3ox-D2**	0.63 ± 0.07	0.46 ± 0.14	0.81 ± 0.56	0.65 ± 0.08	0.10 ± 0.06	0.00 ± 0.00	2.22 ± 1.17	0.63 ± 0.05	0.13 ± 0.02	0.46 ± 0.03	0.72 ± 0.14	2.87 ± 0.45	0.08 ± 0.04	0.08 ± 0.03	0.35 ± 0.01
**TaGA3ox-A3**	0.00 ± 0.00	0.00 ± 0.00	0.00 ± 0.00	0.00 ± 0.00	0.00 ± 0.00	0.00 ± 0.00	0.00 ± 0.00	0.00 ± 0.00	0.00 ± 0.00	0.03 ± 0.03	0.00 ± 0.00	0.00 ± 0.00	0.00 ± 0.00	13.97 ± 0.37	0.00 ± 0.00
**TaGA3ox-B3**	0.02 ± 0.02	0.14 ± 0.08	0.15 ± 0.05	0.00 ± 0.00	0.00 ± 0.00	0.00 ± 0.00	0.00 ± 0.00	0.00 ± 0.00	0.00 ± 0.00	0.00 ± 0.00	0.00 ± 0.00	0.00 ± 0.00	0.00 ± 0.00	27.11 ± 2.09	0.00 ± 0.00
**TaGA3ox-D3**	0.00 ± 0.00	0.00 ± 0.00	0.00 ± 0.00	0.00 ± 0.00	0.00 ± 0.00	0.00 ± 0.00	0.00 ± 0.00	0.00 ± 0.00	0.00 ± 0.00	0.00 ± 0.00	0.00 ± 0.00	0.00 ± 0.00	0.00 ± 0.00	0.30 ± 0.09	0.00 ± 0.00
**TaGA1ox-B1**	0.02 ± 0.02	1.04 ± 0.64	0.84 ± 0.36	0.00 ± 0.00	0.00 ± 0.00	0.00 ± 0.00	0.00 ± 0.00	0.00 ± 0.00	0.00 ± 0.00	0.00 ± 0.00	0.00 ± 0.00	0.09 ± 0.00	0.00 ± 0.00	23.60 ± 0.10	0.02 ± 0.02
**TaGA2ox-A1**	0.06 ± 0.03	0.48 ± 0.30	0.39 ± 0.25	0.07 ± 0.00	0.08 ± 0.01	0.07 ± 0.03	0.29 ± 0.00	0.10 ± 0.02	0.07 ± 0.00	0.16 ± 0.01	0.10 ± 0.02	0.18 ± 0.09	0.05 ± 0.02	0.16 ± 0.11	0.13 ± 0.01
**TaGA2ox-B1**	0.09 ± 0.02	0.06 ± 0.03	0.10 ± 0.01	0.03 ± 0.00	0.10 ± 0.03	0.00 ± 0.00	0.36 ± 0.04	0.05 ± 0.02	0.04 ± 0.00	0.05 ± 0.05	0.05 ± 0.01	0.03 ± 0.00	0.02 ± 0.02	0.03 ± 0.03	0.00 ± 0.00
**TaGA2ox-D1**	0.05 ± 0.02	0.19 ± 0.08	0.20 ± 0.05	0.03 ± 0.00	0.03 ± 0.00	0.06 ± 0.03	0.23 ± 0.03	0.05 ± 0.05	0.00 ± 0.00	0.02 ± 0.02	0.05 ± 0.01	0.02 ± 0.02	0.00 ± 0.00	0.00 ± 0.00	0.02 ± 0.02
**TaGA2ox-D2**	0.00 ± 0.00	0.00 ± 0.00	0.00 ± 0.00	0.00 ± 0.00	0.00 ± 0.00	0.00 ± 0.00	0.00 ± 0.00	0.00 ± 0.00	0.00 ± 0.00	0.00 ± 0.00	0.00 ± 0.00	0.00 ± 0.00	0.02 ± 0.02	0.00 ± 0.00	0.00 ± 0.00
**TaGA2ox-A3**	6.07 ± 0.16	3.36 ± 0.79	3.66 ± 1.34	1.18 ± 0.18	0.36 ± 0.15	0.07 ± 0.03	1.33 ± 0.08	3.89 ± 0.12	1.84 ± 0.65	0.53 ± 0.26	0.94 ± 0.02	2.07 ± 0.12	0.53 ± 0.11	0.25 ± 0.06	0.10 ± 0.01
**TaGA2ox-B3**	8.55 ± 0.28	7.25 ± 2.37	8.74 ± 6.05	0.58 ± 0.24	0.27 ± 0.02	0.12 ± 0.08	1.27 ± 0.12	1.80 ± 0.33	0.27 ± 0.03	0.35 ± 0.04	0.26 ± 0.12	1.36 ± 0.22	0.86 ± 0.08	0.70 ± 0.08	0.08 ± 0.03
**TaGA2ox-D3**	5.42 ± 0.45	4.53 ± 0.47	4.90 ± 2.55	0.74 ± 0.04	0.13 ± 0.13	0.05 ± 0.01	1.48 ± 0.07	1.49 ± 0.28	0.80 ± 0.07	0.73 ± 0.12	0.41 ± 0.05	2.42 ± 0.12	0.70 ± 0.21	0.23 ± 0.16	0.39 ± 0.01
**TaGA2ox-A4**	0.36 ± 0.02	5.58 ± 4.02	7.10 ± 6.71	0.90 ± 0.03	3.91 ± 0.79	0.18 ± 0.06	1.20 ± 0.32	5.03 ± 0.35	0.31 ± 0.11	0.19 ± 0.09	0.27 ± 0.27	0.05 ± 0.02	0.04 ± 0.00	0.00 ± 0.00	0.00 ± 0.00
**TaGA2ox-B4**	0.02 ± 0.02	0.11 ± 0.11	0.06 ± 0.06	1.14 ± 0.47	3.14 ± 0.84	0.04 ± 0.01	0.57 ± 0.01	1.37 ± 0.18	0.20 ± 0.04	0.02 ± 0.02	0.04 ± 0.00	0.04 ± 0.00	0.27 ± 0.01	0.00 ± 0.00	0.00 ± 0.00
**TaGA2ox-D4(5BL)**	0.03 ± 0.03	0.31 ± 0.22	0.65 ± 0.57	0.49 ± 0.28	2.11 ± 0.41	0.12 ± 0.03	1.43 ± 0.23	3.62 ± 0.31	0.47 ± 0.05	0.55 ± 0.18	0.39 ± 0.19	0.22 ± 0.04	0.61 ± 0.12	0.00 ± 0.00	0.00 ± 0.00
**TaGA2ox-A6**	0.36 ± 0.07	0.07 ± 0.01	0.03 ± 0.03	0.05 ± 0.05	0.04 ± 0.04	0.00 ± 0.00	0.03 ± 0.00	0.19 ± 0.07	0.02 ± 0.02	0.11 ± 0.04	0.07 ± 0.01	2.17 ± 0.33	0.02 ± 0.02	0.06 ± 0.06	0.51 ± 0.11
**TaGA2ox-B6**	0.58 ± 0.04	0.10 ± 0.01	0.18 ± 0.14	0.19 ± 0.01	0.20 ± 0.02	0.00 ± 0.00	0.04 ± 0.04	0.22 ± 0.14	0.00 ± 0.00	0.02 ± 0.02	0.00 ± 0.00	1.31 ± 0.26	0.08 ± 0.04	0.11 ± 0.05	0.56 ± 0.02
**TaGA2ox-D6**	0.85 ± 0.19	0.24 ± 0.08	0.25 ± 0.14	0.18 ± 0.08	0.07 ± 0.07	0.00 ± 0.00	0.00 ± 0.00	0.21 ± 0.04	0.00 ± 0.00	0.03 ± 0.03	0.00 ± 0.00	2.29 ± 0.53	0.26 ± 0.06	0.08 ± 0.03	0.84 ± 0.00
**TaGA2ox-A7**	0.02 ± 0.02	0.14 ± 0.14	0.02 ± 0.02	0.04 ± 0.04	0.36 ± 0.13	0.09 ± 0.01	0.03 ± 0.03	0.17 ± 0.04	0.59 ± 0.11	0.73 ± 0.22	0.24 ± 0.04	1.50 ± 0.22	5.64 ± 1.20	4.99 ± 0.03	0.05 ± 0.05
**TaGA2ox-B7**	0.06 ± 0.06	0.15 ± 0.11	0.10 ± 0.06	0.19 ± 0.12	0.91 ± 0.17	0.29 ± 0.09	0.17 ± 0.05	0.41 ± 0.02	0.61 ± 0.17	0.91 ± 0.48	0.49 ± 0.21	1.29 ± 0.21	2.97 ± 0.90	12.08 ± 0.42	0.70 ± 0.06
**TaGA2ox-D7**	0.03 ± 0.03	0.48 ± 0.44	0.22 ± 0.06	0.73 ± 0.13	2.09 ± 0.31	0.30 ± 0.01	0.17 ± 0.09	0.70 ± 0.03	0.28 ± 0.00	0.48 ± 0.19	0.45 ± 0.02	3.39 ± 0.96	3.71 ± 0.68	11.75 ± 0.45	2.20 ± 0.13
**TaGA2ox-A8**	0.00 ± 0.00	0.14 ± 0.14	0.04 ± 0.00	0.00 ± 0.00	0.03 ± 0.00	0.00 ± 0.00	0.49 ± 0.12	0.24 ± 0.04	0.00 ± 0.00	0.22 ± 0.02	0.14 ± 0.05	0.30 ± 0.06	0.02 ± 0.02	0.14 ± 0.03	0.00 ± 0.00
**TaGA2ox-B8**	0.11 ± 0.01	0.19 ± 0.11	0.05 ± 0.01	0.02 ± 0.02	0.10 ± 0.01	0.00 ± 0.00	0.56 ± 0.11	0.73 ± 0.13	0.00 ± 0.00	0.42 ± 0.03	0.13 ± 0.10	0.80 ± 0.12	0.02 ± 0.02	0.48 ± 0.07	0.12 ± 0.03
**TaGA2ox-D8(5BL)**	0.00 ± 0.00	0.00 ± 0.00	0.00 ± 0.00	0.00 ± 0.00	0.02 ± 0.02	0.00 ± 0.00	0.05 ± 0.05	0.00 ± 0.00	0.00 ± 0.00	0.02 ± 0.02	0.05 ± 0.05	3.79 ± 0.75	0.05 ± 0.01	0.00 ± 0.00	0.00 ± 0.00
**TaGA2ox-A9**	3.25 ± 0.71	2.06 ± 1.20	2.27 ± 1.92	0.04 ± 0.00	0.69 ± 0.09	0.73 ± 0.13	1.04 ± 0.10	3.54 ± 0.05	0.04 ± 0.00	4.76 ± 1.97	0.96 ± 0.47	0.00 ± 0.00	1.10 ± 0.26	0.74 ± 0.16	0.32 ± 0.16
**TaGA2ox-D9**	9.77 ± 0.81	2.66 ± 1.20	3.39 ± 2.50	0.21 ± 0.17	2.42 ± 0.31	1.61 ± 0.12	1.42 ± 0.05	13.61 ± 0.07	0.08 ± 0.04	15.91 ± 5.34	5.94 ± 3.14	0.35 ± 0.02	6.06 ± 0.83	1.36 ± 0.21	0.08 ± 0.08
**TaGA2ox-A10**	0.20 ± 0.13	0.82 ± 0.55	0.43 ± 0.35	1.33 ± 0.72	0.10 ± 0.06	0.00 ± 0.00	0.19 ± 0.00	0.58 ± 0.00	0.08 ± 0.00	0.37 ± 0.12	1.01 ± 0.14	0.45 ± 0.02	0.24 ± 0.01	0.06 ± 0.00	0.18 ± 0.03
**TaGA2ox-B10**	0.45 ± 0.06	0.27 ± 0.03	0.20 ± 0.12	1.57 ± 0.42	0.27 ± 0.03	0.00 ± 0.00	0.57 ± 0.30	1.24 ± 0.01	0.11 ± 0.08	0.36 ± 0.00	0.73 ± 0.08	0.57 ± 0.27	0.75 ± 0.09	1.07 ± 0.00	0.04 ± 0.04
**TaGA2ox-D10**	1.34 ± 0.15	0.54 ± 0.25	0.78 ± 0.67	0.78 ± 0.16	0.23 ± 0.03	0.02 ± 0.02	0.14 ± 0.05	0.54 ± 0.04	0.13 ± 0.02	0.56 ± 0.07	0.28 ± 0.15	0.93 ± 0.05	0.28 ± 0.03	0.90 ± 0.07	0.07 ± 0.02
**TaGA2ox-A11**	0.00 ± 0.00	0.00 ± 0.00	0.02 ± 0.02	0.00 ± 0.00	0.00 ± 0.00	0.00 ± 0.00	0.00 ± 0.00	0.00 ± 0.00	0.00 ± 0.00	0.00 ± 0.00	0.00 ± 0.00	0.00 ± 0.00	0.00 ± 0.00	0.00 ± 0.00	0.00 ± 0.00
**TaGA2ox-B11**	0.00 ± 0.00	0.00 ± 0.00	0.00 ± 0.00	0.00 ± 0.00	0.00 ± 0.00	0.00 ± 0.00	0.00 ± 0.00	0.00 ± 0.00	0.00 ± 0.00	0.00 ± 0.00	0.00 ± 0.00	0.00 ± 0.00	0.00 ± 0.00	0.00 ± 0.00	0.05 ± 0.05
**TaGA2ox-D11**	0.00 ± 0.00	0.00 ± 0.00	0.00 ± 0.00	0.04 ± 0.04	0.06 ± 0.06	0.02 ± 0.02	0.00 ± 0.00	0.00 ± 0.00	0.00 ± 0.00	0.00 ± 0.00	0.00 ± 0.00	0.00 ± 0.00	0.00 ± 0.00	0.00 ± 0.00	0.00 ± 0.00
**TaGA2ox-B12**	0.00 ± 0.00	0.00 ± 0.00	0.01 ± 0.01	0.00 ± 0.00	0.04 ± 0.00	2.16 ± 0.27	0.00 ± 0.00	0.16 ± 0.04	0.36 ± 0.01	0.02 ± 0.02	0.02 ± 0.02	0.14 ± 0.07	0.22 ± 0.05	0.00 ± 0.00	0.00 ± 0.00
**TaGA2ox-B13**	0.00 ± 0.00	0.00 ± 0.00	0.02 ± 0.02	0.00 ± 0.00	0.00 ± 0.00	0.02 ± 0.02	0.00 ± 0.00	0.00 ± 0.00	0.00 ± 0.00	0.00 ± 0.00	0.02 ± 0.02	0.04 ± 0.04	0.00 ± 0.00	0.00 ± 0.00	0.00 ± 0.00
**TaGID-A1**	31.26 ± 4.04	57.78 ± 17.49	53.81 ± 15.21	4.74 ± 0.34	16.74 ± 1.04	54.36 ± 1.57	11.21 ± 2.26	36.51 ± 5.03	4.64 ± 0.27	23.84 ± 0.79	16.86 ± 3.47	25.77 ± 4.44	29.27 ± 3.61	14.47 ± 0.27	6.85 ± 0.67
**TaGID-B1**	22.44 ± 3.27	36.64 ± 11.15	33.61 ± 4.84	4.32 ± 0.17	10.20 ± 1.83	36.62 ± 1.25	9.47 ± 2.51	32.82 ± 1.85	7.50 ± 0.21	19.09 ± 2.37	13.60 ± 4.63	22.84 ± 5.00	28.56 ± 3.37	8.63 ± 1.14	10.13 ± 0.50
**TaGID-D1**	23.63 ± 4.95	47.40 ± 18.92	40.41 ± 4.90	3.28 ± 0.48	9.33 ± 1.41	35.02 ± 2.98	10.14 ± 1.85	33.83 ± 6.66	6.59 ± 0.20	15.33 ± 0.64	13.42 ± 1.89	26.44 ± 6.02	24.39 ± 3.50	14.46 ± 0.56	8.73 ± 0.95
**TaGID-A2**	1.34 ± 0.06	15.75 ± 10.65	9.86 ± 4.69	2.18 ± 0.89	2.75 ± 0.35	4.24 ± 0.65	3.58 ± 0.21	1.93 ± 0.92	5.59 ± 1.32	0.41 ± 0.06	3.55 ± 1.00	2.62 ± 0.47	1.62 ± 0.32	0.54 ± 0.22	0.07 ± 0.07
**TaGID-B2**	0.35 ± 0.26	0.58 ± 0.25	1.01 ± 0.69	0.19 ± 0.01	0.15 ± 0.06	0.34 ± 0.34	0.44 ± 0.20	0.28 ± 0.05	0.72 ± 0.29	0.31 ± 0.12	0.42 ± 0.21	0.43 ± 0.34	0.65 ± 0.37	0.61 ± 0.14	0.37 ± 0.09
**TaGID-D2**	0.98 ± 0.25	8.05 ± 3.75	10.82 ± 7.84	0.80 ± 0.02	1.36 ± 0.22	2.79 ± 0.76	1.75 ± 0.25	1.18 ± 0.18	7.59 ± 0.55	1.29 ± 0.45	4.39 ± 0.65	3.21 ± 0.41	2.88 ± 0.39	0.61 ± 0.14	0.53 ± 0.30
**TaRht-A1**	7.93 ± 0.21	20.31 ± 6.14	19.38 ± 8.86	10.02 ± 2.15	12.44 ± 0.62	15.20 ± 0.35	24.75 ± 4.79	34.68 ± 2.27	28.23 ± 0.01	18.59 ± 0.17	23.23 ± 0.03	31.23 ± 2.26	12.19 ± 1.65	5.43 ± 0.10	24.51 ± 0.14
**TaRht-B1**	4.05 ± 0.00	7.60 ± 0.58	9.24 ± 4.01	5.89 ± 0.60	7.00 ± 0.51	6.35 ± 0.16	15.89 ± 4.22	14.98 ± 0.45	11.87 ± 0.46	10.57 ± 0.19	11.56 ± 1.04	13.73 ± 0.93	6.52 ± 0.72	2.51 ± 0.03	29.37 ± 0.33
**TaRht-D1**	6.81 ± 0.58	15.58 ± 3.01	16.05 ± 8.01	7.61 ± 1.11	9.45 ± 0.14	11.86 ± 0.84	19.06 ± 5.00	18.60 ± 1.41	20.04 ± 0.05	13.88 ± 0.44	17.92 ± 0.03	23.25 ± 1.49	8.98 ± 1.23	3.01 ± 0.11	18.82 ± 0.90
