# Supplementary material for: Heterologous expression and transcript analysis of gibberellin biosynthetic genes of grasses reveals novel functionality in the GA3ox family
Source: BMC Plant Biol. 2015 Jun 5;15:130. doi: 10.1186/s12870-015-0520-7 (PMC4455330; doi:10.1186/s12870-015-0520-7)
Supplement: Supplementary file 4 — qRT-PCR primers used in this study and qRT-PCR methods. [file 12870_2015_520_MOESM4_ESM.pdf]

Additional file 4: qRT-PCR primers used in this study.

Target	F primer (5’- 3’)	R primer (5’- 3’)	Efficiency (%)
CPS	CGCAAAATGCTTCGAGTACA	GAGATTCCAAGACGCTCCAG	91.3
KS	CTCCATGCTTCCCTCAATGT	TGGCCAAGATTCCATTTCTC	96.3
KO	CAGAAAAACAGGATCGGCTC	ACCAGTGGGACAGGAGAATG	97.1
KAO	TCGTCAACATCTCCTTCGTG	TGAGGGTACACCTGAGGGTC	99.1
GA13ox1	GAGCCTACTCCGCAGATGTC	AGAACCTTAGGCCGGTCATT	94.6
GA13ox2	GAGCTCAAGGTGCTGCTCTC	CACCGGCGAGTGTAGGTAGT	93.4
GA20ox1	GACCATCCTCCACCAGGAC	TGGAGGCAGCTCTTGTACCT	92.4
GA20ox2	CCCTGGAAGGAGACCCTCT	GGGTGCTGGTGAAGTAGTCC	93.5
GA20ox3	ACCGTGTCCTTCAACTGCTC	CCCATGTCACGGTACTCCTC	98.0
GA20ox4	GTAAACATCGGCGACACCTT	GTGGAGGCAGCTCTTGTACC	93.0
GA3ox2	AGGTCGCCGCCGTCGAGTCC	CAGTTGAGGTGCATTGTGGC	91.8
GA3ox3	GTGATGCAGAGCCACGTC	TGAGGATCTGGAAGAGGTCA	97.6
GA1ox1	AGTAGGGCCGCTTAAGGAAG	GGTTGATGGAGACCATCTCG	92.5
GA2ox1	AATGTACTCGCTCCGCCTAA	ATTTTCCTTTGCTGCCATTG	91.8
GA2ox3	GACCCCGGGGACTACTTCT	TTGACGAGCTTGAAGAACCC	99.5
GA2ox4	AGCAGATCACGCTGCTGAG	GTTCACGACGCTGAAGAACC	96.2
GA2ox6	GTTCGACACCAAGGAGAAGG	GAGCTGAGCTTCCCGTAGTC	98.2
GA2ox7	CTTCGCGTCCTGGACATTG	ACTCCTCCAGAAGATCCCGT	98.8
GA2ox8	GCACAAGAGCAAGCTGGAG	CCGACGGAGAGAAGGATGTA	89.2
GA2ox9	TCTCAACGACTCCTACCGCT	ATGGAAGGCCTCAGACCAG	89.5
GA2ox10	TCGAGTATCTCCTGCTCGGT	ACGACCTCCGTGTACTCGTT	99.6
GID1	CACACATGGGTGCTCATCTC	GATGAAGTGGTCGAAGGAGG	93.3
GID2	GAGGACGTGATGTTCGAGGT	GAGCAGCTGTTCCGAGAAG	96.2
Rht-1	GAAGCAGATACCCTTGCTGG	GGGCAGGACTCGTAGAAGTG	94.6
ACTIN	ACCTTCAGTTGCCCAGCAAT	CAGAGTCGAGCACAATACCAGTTG	97.7

Expression analysis by qRT-PCR

RNA was extracted from shoot, root, spike and grain tissues from the tetraploid cv. ‘Kronos’ as described in Krasileva et al. (2013). Total RNA was extracted from ground tissues using the Spectrum™ Plant Total RNA kit (Sigma-Aldrich, St. Louis, MO) and 1 µg of the RNA equivalent of cDNA was synthesized using Quantitect Reverse Transcription Kit (Qiagen, Valencia, CA). qRT-PCR reactions were performed on an Applied Biosystems 7500 Fast Real-Time PCR System machine (Applied Biosystems, Foster City, CA). Each reaction was carried out in a 20 µl volume and consisted of 1x USB® VeriQuest™ SYBR® Green qPCR Master Mix (Affymetrix, Santa Clara, CA), 0.5 µM of F and R primers and 10 ng of cDNA. *ACTIN* was used as an endogenous control gene and all qRT-PCR primers used in this study and their efficiencies are above. All primers were checked for efficiency and specificity by analyzing their amplification in a four-fold dilution series and checking that each reaction yielded a single product by studying the derived dissociation curve. Transcript levels are reported as the proportional distribution of transcripts from each gene across the four tissues.
