# Supplementary material for: Heterologous expression and transcript analysis of gibberellin biosynthetic genes of grasses reveals novel functionality in the GA3ox family
Source: BMC Plant Biol. 2015 Jun 5;15:130. doi: 10.1186/s12870-015-0520-7 (PMC4455330; doi:10.1186/s12870-015-0520-7)
Supplement: Supplementary file 5 — Homoeolog-specific expression of GA biosynthetic and signalling genes in different layers of the wheat grain. [file 12870_2015_520_MOESM5_ESM.pdf]

Additional file 5: Homoeologue-specific GA gene transcript levels in grain tissues. FKPM values for genes from the GA pathway, ± standard error (n=3)

Genes	Endosperm	Inner Pericarp	Outer Pericarp
**TaCPS-A1**	2.80 ± 0.39	0.52 ± 0.18	0.65 ± 0.10
**TaCPS-B1**	0.53 ± 0.15	0.73 ± 0.07	1.28 ± 0.13
**TaCPS-D1**	0.83 ± 0.08	0.48 ± 0.08	1.08 ± 0.16
**TaKS-B1**	4.53 ± 0.45	2.74 ± 0.24	1.70 ± 0.28
**TaKS-D1**	1.28 ± 0.17	2.81 ± 0.24	2.83 ± 0.34
**TaKO-A1**	28.89 ± 1.38	17.42 ± 2.51	10.71 ± 1.25
**TaKO-B2**	22.87 ± 1.26	9.40 ± 0.67	2.68 ± 0.33
**TaKO-D1**	12.33 ± 0.79	7.29 ± 0.99	2.95 ± 0.31
**TaKAO-A1**	33.41 ± 2.27	18.38 ± 1.40	2.62 ± 0.24
**TaKAO-D1**	11.42 ± 1.37	5.63 ± 0.19	0.99 ± 0.21
**TaKAO-A2**	18.24 ± 0.93	10.38 ± 0.67	2.10 ± 0.32
**TaGA13ox-A1**	0.09 +/- 0.06	2.80 +/- 0.57	28.95 +/- 2.06
**TaGA13ox-B1**	0.00 +/- 0.00	0.38 +/- 0.06	12.08 +/- 0.96
**TaGA13ox-D1**	0.06 +/- 0.06	2.68 +/- 0.27	33.58 +/- 2.30
**TaGA13ox-A2**	0.04 +/- 0.04	2.14 +/- 0.17	10.35 +/- 0.99
**TaGA13ox-B2**	0.04 +/- 0.04	1.70 +/- 0.25	8.80 +/- 1.02
**TaGA13ox-D2**	0.27 +/- 0.02	0.43 +/- 0.10	4.43 +/- 0.68
**TaGA20ox-A1**	0.00 ± 0.00	0.76 ± 0.21	0.81 ± 0.20
**TaGA20ox-B1**	0.00 ± 0.00	0.00 ± 0.00	0.31 ± 0.15
**TaGA20ox-D1**	0.12 ± 0.08	0.18 ± 0.12	1.28 ± 0.23
**TaGA20ox-A2**	0.00 ± 0.00	0.00 ± 0.00	0.05 ± 0.05
**TaGA20ox-B2**	0.00 ± 0.00	0.06 ± 0.06	0.05 ± 0.05
**TaGA20ox-D2**	0.00 ± 0.00	0.11 ± 0.07	0.45 ± 0.18
**TaGA20ox-A3**	5.56 ± 0.83	3.46 ± 0.67	1.00 ± 0.17
**TaGA20ox-B3**	13.00 ± 0.70	6.79 ± 0.81	0.96 ± 0.26
**TaGA20ox-D3**	1.45 ± 0.54	0.44 ± 0.16	0.09 ± 0.06
**TaGA20ox-A4**	0.12 ± 0.07	1.78 ± 0.60	8.88 ± 1.30
**TaGA20ox-B4**	0.12 ± 0.07	0.14 ± 0.06	1.32 ± 0.26
**TaGA20ox-D4**	0.13 ± 0.08	0.38 ± 0.09	5.87 ± 0.65
**TaGA3ox-A2**	0.00 ± 0.00	0.10 ± 0.07	0.19 ± 0.09
**TaGA3ox-B2**	0.00 ± 0.00	0.00 ± 0.00	0.00 ± 0.00
**TaGA3ox-D2**	0.00 ± 0.00	0.00 ± 0.00	0.10 ± 0.06
**TaGA3ox-A3**	3.59 ± 0.18	1.30 ± 0.50	0.24 ± 0.08
**TaGA3ox-B3**	72.78 ± 2.00	36.34 ± 2.40	5.20 ± 0.75
**TaGA3ox-D3**	1.67 ± 0.16	0.46 ± 0.13	0.04 ± 0.04
**TaGA1ox-B1**	76.66 ± 3.35	34.42 ± 1.51	4.85 ± 0.35
**TaGA2ox-A1**	0.00 ± 0.00	0.06 ± 0.06	0.00 ± 0.00
**TaGA2ox-B1**	0.00 ± 0.00	0.07 ± 0.07	0.05 ± 0.05
**TaGA2ox-D1**	0.07 ± 0.07	0.26 ± 0.08	0.00 ± 0.00
**TaGA2ox-D2**	0.00 ± 0.00	0.00 ± 0.00	0.00 ± 0.00
**TaGA2ox-A3**	0.00 ± 0.00	0.14 ± 0.09	2.23 ± 0.56
**TaGA2ox-B3**	0.00 ± 0.00	0.06 ± 0.06	1.76 ± 0.34
**TaGA2ox-D3**	0.06 ± 0.06	0.06 ± 0.06	0.65 ± 0.28
**TaGA2ox-A4**	0.00 ± 0.00	0.00 ± 0.00	0.00 ± 0.00
**TaGA2ox-B4**	0.00 ± 0.00	0.00 ± 0.00	0.14 ± 0.09
**TaGA2ox-D4(5BL)**	0.00 ± 0.00	0.07 ± 0.07	0.27 ± 0.13
**TaGA2ox-A6**	0.00 ± 0.00	0.00 ± 0.00	0.00 ± 0.00
**TaGA2ox-B6**	0.00 ± 0.00	0.00 ± 0.00	0.00 ± 0.00
**TaGA2ox-D6**	0.00 ± 0.00	0.00 ± 0.00	0.00 ± 0.00
**TaGA2ox-A7**	0.25 ± 0.11	20.93 ± 1.67	13.12 ± 2.91
**TaGA2ox-B7**	1.30 ± 0.30	62.57 ± 6.48	23.39 ± 3.98
**TaGA2ox-D7**	1.65 ± 0.50	72.92 ± 2.97	17.71 ± 1.90
**TaGA2ox-A8**	0.00 ± 0.00	0.11 ± 0.07	0.00 ± 0.00
**TaGA2ox-B8**	0.00 ± 0.00	0.08 ± 0.08	0.00 ± 0.00
**TaGA2ox-D8(5BL)**	0.00 ± 0.00	0.06 ± 0.06	0.00 ± 0.00
**TaGA2ox-A9**	0.00 ± 0.00	0.00 ± 0.00	0.55 ± 0.29
**TaGA2ox-D9**	0.00 ± 0.00	0.06 ± 0.06	0.80 ± 0.29
**TaGA2ox-A10**	0.39 ± 0.22	0.34 ± 0.19	0.43 ± 0.14
**TaGA2ox-B10**	0.14 ± 0.09	0.19 ± 0.12	0.32 ± 0.16
**TaGA2ox-D10**	0.59 ± 0.20	0.16 ± 0.07	0.09 ± 0.06
**TaGA2ox-A11**	0.00 ± 0.00	0.00 ± 0.00	0.00 ± 0.00
**TaGA2ox-B11**	0.00 ± 0.00	0.00 ± 0.00	0.00 ± 0.00
**TaGA2ox-D11**	0.00 ± 0.00	0.00 ± 0.00	0.00 ± 0.00
**TaGA2ox-B12**	0.00 ± 0.00	0.00 ± 0.00	0.00 ± 0.00
**TaGA2ox-B13**	0.00 ± 0.00	0.00 ± 0.00	0.00 ± 0.00
**TaGID-A1**	0.72 ± 0.33	5.34 ± 0.65	21.13 ± 1.58
**TaGID-B1**	0.52 ± 0.18	2.82 ± 0.45	8.67 ± 0.95
**TaGID-D1**	0.77 ± 0.16	4.80 ± 0.20	21.89 ± 1.64
**TaGID-A2**	0.00 ± 0.00	3.82 ± 0.63	2.49 ± 0.36
**TaGID-B2**	1.16 ± 0.45	1.68 ± 0.70	1.69 ± 0.27
**TaGID-D2**	0.20 ± 0.20	4.72 ± 1.03	4.41 ± 0.75
**TaRht-A1**	0.65 ± 0.16	2.49 ± 0.26	14.96 ± 1.06
**TaRht-B1**	0.12 ± 0.09	0.86 ± 0.14	6.20 ± 0.53
**TaRht-D1**	0.41 ± 0.13	1.16 ± 0.25	4.72 ± 0.51
