# Supplementary material for: Heterologous expression and transcript analysis of gibberellin biosynthetic genes of grasses reveals novel functionality in the GA3ox family
Source: BMC Plant Biol. 2015 Jun 5;15:130. doi: 10.1186/s12870-015-0520-7 (PMC4455330; doi:10.1186/s12870-015-0520-7)
Supplement: Supplementary file 7 — Sequences in the IWGSC reference used for RNA-seq mapping substituted with full-length CDS sequences from this work. [file 12870_2015_520_MOESM7_ESM.pdf]

Additional file 6: Sequence substitution in the wheat transcriptome reference used for mapping RNA-seq data

Ensemble v_23 entry	Replaced with
Traes_7AL_DDB77B9FF.1	TaCPS-A1
Traes_7AL_7B5ACA69E.2	TaCPS-A1
Traes_7BL_CADA931D8.1	TaCPS-B1
Traes_7BL_70E7494E5.1	TaCPS-B1
Traes_7BL_6799422C4.1	TaCPS-B1
Traes_7BL_02F3E2C78.1	TaCPS-B1
Traes_7BL_4B1AFE613.1	TaCPS-B1
Traes_7DL_4C9302CF2.2	TaCPS-D1
Traes_7DL_02E7F7D6A.1	TaCPS-D1
Traes_2BL_295397D93.2	TaKS-B1
Traes_2DL_644383DE2.1	TaKS-D1
Traes_2DL_3AB49D941.1	TaKS-D1
Traes_2DL_3098F1277.1	TaKS-D1
Traes_2AL_57C9471E6.2	TaKS-D1
Traes_7AL_73FE321EC.1	TaKO-A1
Traes_7AL_12B79D25B.1	TaKO-A1
Traes_7BL_FCFFAF5EC.1	TaKO-B2
Traes_7BL_8449CBECD.1	TaKO-B2
Traes_7DL_7921C2DAB.1	TaKO-D1
Traes_4AL_795326026.1	TaKAO-A1
Traes_7AS_F627F2ED4.1	TaKAO-A2
Traes_7DS_F5B0CE6B8.1	TaKAO-D1
Traes_7DS_760FDD8CD.1	TaKAO-D1
Traes_7DS_257B34BF1.1	TaKAO-D1
Traes_4AS_C8CB45702.1	TaGA13ox-1A
Traes_4BL_AC29E521A.1	TaGA13ox-1B
Traes_4DL_C8977947C.1	TaGA13ox-1D
Traes_2AS_DC1F087DE.1	TaGA13ox-2A
Traes_2BS_0C7F47373.1	TaGA13ox-2B
Traes_2BS_82FCD24BE.1	TaGA13ox-2B
Traes_2DS_4AABBA9E6.1	TaGA13ox-2D
Traes_2DS_7E1450B03.1	TaGA13ox-2D
Traes_4AL_FABDF4EDA.1	TaGA20ox-A1
Traes_1AL_FB0466848.1	TaGA20ox-A4
Traes_1AL_3A716350F.2	TaGA20ox-A4
Traes_5BL_D412D28CC.2	TaGA20ox-B1
Traes_3B_A2E5CB642.1	TaGA20ox-B2
Traes_3B_763D7ABA2.1	TaGA20ox-B3
Traes_1BL_BCA1AABF7.1	TaGA20ox-B4
Traes_1BL_32506F819.1	TaGA20ox-B4
Traes_5DL_3E77D28A6.1	TaGA20ox-D1
Traes_1DL_9E65B5F06.1	TaGA20ox-D4
Traes_1AS_2EC304B15.1	TaGA2ox-A1
Traes_1AS_B90725283.1	TaGA2ox-A10
Traes_1AS_570581E09.2	TaGA2ox-A10
Traes_1AS_242A61BF9.1	TaGA2ox-A10
Traes_4AS_7DC625FF5.1	TaGA2ox-A11
Traes_3AL_14A36F545.1	TaGA2ox-A3
Traes_1AL_5ED72B143.1	TaGA2ox-A4
Traes_2AL_BA387175F.1	TaGA2ox-A6
Traes_1AL_C6975BBBD.1	TaGA2ox-A8
Traes_6AL_6926D08B4.1	TaGA2ox-A9
Traes_1BS_0EDD50331.1	TaGA2ox-B1
Traes_1BS_2C29ED3EF.1	TaGA2ox-B10
Traes_4BS_B52D1D505.1	TaGA2ox-B11
Traes_4BL_63EFE8C91.1	TaGA2ox-B11
Traes_4BL_57623F302.1	TaGA2ox-B12
Traes_3B_7ABEA6AAD.2	TaGA2ox-B3
Traes_3B_4585C2A52.1	TaGA2ox-B3
Traes_1BL_97C47ECEA.1	TaGA2ox-B4
Traes_2BL_1B1358201.1	TaGA2ox-B6
Traes_3B_0CC70372F.2	TaGA2ox-B7
Traes_1BL_AAA0842AB.1	TaGA2ox-B8
Traes_1BL_A1CF1385F.1	TaGA2ox-B8
Traes_1DS_125A395C1.1	TaGA2ox-D1
Traes_1DS_A44358D5B.2	TaGA2ox-D10
Traes_4DL_5D396447B.1	TaGA2ox-D11
Traes_3DL_857F47A93.1	TaGA2ox-D3
Traes_5BL_8123B1AD3.1	TaGA2ox-D4(5BL)
Traes_2DL_5E0E44CA3.1	TaGA2ox-D6
Traes_2DL_025DD4C90.1	TaGA2ox-D6
Traes_6DL_CB24C57DF.1	TaGA2ox-D9
Traes_6DL_0AF913208.1	TaGA2ox-D9
Traes_6BL_B8497D305.2	TaGA2ox-D9
Traes_3AS_3A79F81AF.1	TaGA3ox-A2
Traes_2AL_B8AB48108.1	TaGA3ox-A3
Traes_2AL_85471F53F.1	TaGA3ox-A3
Traes_2AL_65B19CC73.1	TaGA3ox-A3
Traes_3B_791A6E8DF.1	TaGA3ox-B2
Traes_3B_3047AACC7.1	TaGA3ox-B2
Traes_2BL_9E115B19F.1	TaGA1ox-B1
Traes_2BL_FF2BB4801.1	TaGA3ox-B3
Traes_2DL_66F9CEA3C.1	TaGA3ox-D3
Traes_3AS_794DAF12D.1	TaGID-A2
Traes_1BL_7517181F2.2	TaGID-B1
Traes_3B_F6CE72D8D.2	TaGID-B2
Traes_3DS_DF23FE973.1	TaGID-D2
Traes_4AL_77F4CC833.1	TaRht-A1
Traes_4BS_5E37B93A8.1	TaRht-B1
Traes_4BS_2EE4988CD.1	TaRht-B1
Traes_4DS_ADBA6E30A.1	TaRht-D1
